# Unsupervised embedding of trajectories captures the latent structure of scientific migration

**DOI:** 10.1073/pnas.2305414120

**Published:** 2023-12-22

**Authors:** Dakota Murray, Jisung Yoon, Sadamori Kojaku, Rodrigo Costas, Woo-Sung Jung, Staša Milojević, Yong-Yeol Ahn

**Affiliations:** ^a^Center for Complex Networks and Systems Research, Luddy School of Informatics, Computing, and Engineering, Indiana University, Bloomington, IN 47408; ^b^Network Science Institute at Northeastern University, Boston, MA 02115; ^c^Kellogg School of Management & Organizations at Northwestern University, Evanston, IL 60208; ^d^Northwestern Institute on Complex Systems, Evanston, IL 60208; ^e^Centre for Science and Technology Studies, Leiden University, 2300 AXLeiden, The Netherlands; ^f^Centre of Excellence in Scientometrics and Science, Technology and Innovation Policy, Stellenbosch University, Stellenbosch 7600, South Africa; ^g^Department of Physics, Pohang University of Science and Technology, Pohang 37673, South Korea; ^h^Department of Industrial and Management Engineering, Pohang University of Science and Technology, Pohang 37673, South Korea

**Keywords:** neural embedding, mobility, migration, word2vec, bibliometrics

## Abstract

Human migration and mobility drives major societal phenomena including epidemics, economies, innovation, and the diffusion of ideas. Although human mobility and migration have been heavily constrained by geographic distance throughout the history, advances, and globalization are making other factors such as language and culture increasingly more important. Advances in neural embedding models, originally designed for natural language, provide an opportunity to tame this complexity and open new avenues for the study of migration. Here, we demonstrate the ability of the model word2vec to encode nuanced relationships between discrete locations from migration trajectories, producing an accurate, dense, continuous, and meaningful vector-space representation. The resulting representation provides a functional distance between locations, as well as a “digital double” that can be distributed, re-used, and itself interrogated to understand the many dimensions of migration. We show that the unique power of word2vec to encode migration patterns stems from its mathematical equivalence with the gravity model of mobility. Focusing on the case of scientific migration, we apply word2vec to a database of three million migration trajectories of scientists derived from the affiliations listed on their publication records. Using techniques that leverage its semantic structure, we demonstrate that embeddings can learn the rich structure that underpins scientific migration, such as cultural, linguistic, and prestige relationships at multiple levels of granularity. Our results provide a theoretical foundation and methodological framework for using neural embeddings to represent and understand migration both within and beyond science.

## Significance

We show that the word embedding technique word2vec is mathematically equivalent to the gravity law of mobility, making it ideal for learning dense representations from migration data that can be distributed, re-used, and studied. By treating locations analogously to words and trajectories to sentences, word2vec embeds each location into a vector space, where the distance reflects rates of migration according to the gravity law. We demonstrate the power of word2vec by applying it to the case of scientists’ migrations, for which it encodes information about culture, geography, and prestige at multiple layers of granularity. Our results lay a theoretical and methodological foundation for the application of neural embeddings to the study of migration.

How far apart are two places? The question is surprisingly hard to answer when it involves human migration and mobility. Although geographic distance has historically constrained human movements, it is becoming less relevant in a world increasingly interconnected by rapid communications and travel. For instance, a person living in Australia is more likely to migrate to the United Kingdom, a far-away country with similar language and culture, than to a much closer country such as Indonesia (1). Similarly, a student in South Korea is more likely to attend a university in Canada than one in neighboring North Korea (2). Although geographic distance has been used as the most prominent basis for models of migration and mobility, such as the gravity (3) and radiation (4) models, the diminishing relevance of geography calls for alternative ways of conceptualizing “distance” (5–7).

Yet, functional distances are often low-resolution, computed at the level of countries rather than regions, cities, or organizations, and have focused on only a single facet of migration at a time. By contrast, real-world migration is multi-faceted, influenced simultaneously by geography, language, culture, history, and economic opportunity. Low-dimensional distance alone cannot represent the multitude of inter-related factors that drive migration. Although networks have been explored as a solution to representing many dimensions of migration, edges only encode simple, dyadic relationships between connected entities. Capturing the complexity of migration requires moving beyond simple functional distances and networks, to learning high-dimensional landscapes of migration that incorporate many facets of migration into a single fine-grained and continuous representation. Such a representation can be used not only to measure distances at multiple scales but also to act as a convenient “digital double,” an entire functional topology that can be distributed, incorporated into future analyses, and itself interrogated to reveal fundamental insights into patterns of global migration.

Here, we demonstrate that the word2vec model (Skip-Gram Negative Sampling) (8) is equivalent to the gravity law of mobility, a fundamental framework used to model migration across many domains. We then empirically test the resulting representation by its ability to derive the functional distances between locations from migration trajectories. After validating its accurate representation of real-world data, we apply a variety of techniques that leverage the unique and powerful semantic structure of the embedding space to study scientific migration. Doing so demonstrates word2vec’s capacity to encode rich information related to geography, culture, language, and even prestige, at multiple scales of analysis.

While the word2vec model shown here can be applied across domains of migration, here we demonstrate its applicability by applying it to study scientific migration. Scientific migration is a central driver of the globalized scientific enterprise (9, 10) and it is strongly related to innovation (11, 12), impact (13, 14), collaboration (15), and the diffusion of knowledge (11, 16). Researchers migrate between organizations as they attain new roles throughout their careers, often motivated by the desire to expand their professional networks (17), to gain access to prestigious institutions (18), to gain entry into high-performing research groups (19), or to obtain resources for research (20). Their choice of destination is however constrained by many factors, including rigid prestige hierarchies that shape faculty hiring (21, 22), language (23), visa and immigration policies (24), and family considerations (19, 25). In spite of its importance, holistic understandings of global scientific migration have been limited by the sheer scope and complexity of the phenomenon (22, 26), being further confounded by the diminishing role of geography in shaping the landscape of scientific migration. Its known structural properties combined with the difficulty of its study at scale make scientific migration the ideal case study for application of word2vec.

**Fig. 1.**
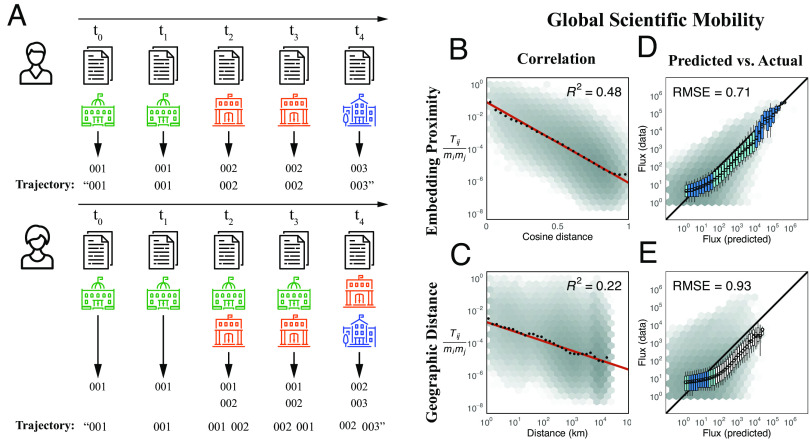
Neural embedding provides functional distance that improves predictive power of the gravity model of migration best across three distinct human trajectory datasets. (*A*) A unique identifier is assigned to each organization and they are assembled into an affiliation trajectory ordered by year of publication (*Top*). If an author lists multiple organization affiliations within the same year, we shuffle the order within that year in each training iteration (*Bottom*, see *SI Appendix*). (*B*) Embedding distance better explains the expected flux of global scientific migration than does geographic distance (*C*). The red line is the line of the best fit. Black dots are mean flux across binned distances. The 99% CIs are plotted for the mean flux in each bin. Correlation is calculated on the data in the log-log scale (P<0.0001 across all fits). The lightness of each hex bin indicates the frequency of organization pairs within it. (*D*) Predictions of flux between institutions made using embedding distance outperform those made using geographic distance (*E*). Box-plots show the distribution of actual flux for binned values of predicted flux. Box color corresponds to the degree to which the distribution overlaps with y=x. “RMSE” is the root-mean-squared error between the actual and predicted values. Embedding distance consistently produces powerful functional distance for U.S. flight itineraries and Korean accommodation reservations (*SI Appendix*).

Trajectories of scientific migration are constructed using more than three million name-disambiguated authors who were *mobile*—having more than one affiliation—between 2008 and 2019, as evidenced by their publications indexed in the Web of Science database (27) (*Materials and Methods*). As a scientist’s career progresses, they move between organizations or pick up additional (simultaneous) affiliations forming *affiliation trajectories* (Fig. 1*A*). Thus, the trajectories encode both migration and co-affiliation—the holding of multiple simultaneous affiliations involving the sharing of time and capital between locations—that is typical of scientific migration (13, 15) (*SI Appendix*). This particular intricacy of scientific migration further illustrates how word2vec can be applied to even the most complex domains. We also apply this technique to U.S. passenger flight itinerary records and Korean accommodation reservations (Detailed descriptions are available in *Materials and Methods*) in order to demonstrate its applicability to incredibly distinct domains of migration and mobility.

Here, we study the skip-gram negative sampling (SGNS), or word2vec neural-network architecture (*Materials and Methods*). This neural embedding model, originally designed as a language model (8), made breakthroughs by revealing novel insights into texts (28–33), networks (34–36) and trajectories (37–42). It works under the notion that a good representation should facilitate prediction, learning a mapping between words can predict a target word based on its context (surrounding words). The model is also computationally efficient, robust to noise, and can encode relations between entities as geometric relationships in the vector space (30, 33, 43–45). When applied to the trajectory data, each location is encoded into a vector space, and vectors relate to one another based on the likelihood of locations appearing adjacent to one another in the same trajectory. Also, word2vec can be interpreted as a kind of metric recovery, which recovers the underlying metric of the semantic manifold (45). Although more sophisticated embedding techniques (46, 47), some adapted toward migration data (47–51), have been developed, the standard word2vec remains a powerful model for representing migration data, owing to its simplicity, intuitiveness, and accessibility. Establishing a theoretical and methodological foundation for word2vec is essential for better understanding and application of other more sophisticated models.

The gravity model framework (3) is a widely used, fundamental migration model (52–55) that connects the expected flux, ${\hat{T}}_{\mathrm{ij}}$, between locations based on their populations and distance:

[1] $$\begin{array}{c} {\hat{T}}_{\mathrm{ij}}=Cm_{i}m_{j}f\left(r_{\mathrm{ij}}\right), \end{array}$$

where $m_{i}$ is the population of location i, $f(r_{\mathrm{ij}})$ is a decay function with respect to distance between locations, and C is a constant estimated from data (*Materials and Methods*). Here, we use the mean annual number of unique mobile and non-mobile authors who were affiliated with each organization. ${\hat{T}}_{\mathrm{ij}}$ or “expected flux” (4), as the expected frequency of the co-occurrence of location i and j in the trajectory in the gravity model.

The gravity model posits that the expected flow, ${\hat{T}}_{\mathrm{ij}}$, (${\hat{T}}_{\mathrm{ij}}={\hat{T}}_{\mathrm{ji}}$), is proportional to the locations’ population, ${\hat{T}}_{\mathrm{ij}}\propto m_{i}m_{j}$, and decays as a function of their distance, $f(r_{\mathrm{ij}})$. Traditionally, the decay function has been defined in terms of geographic distance, due to its intuitiveness and availability. Here, we also consider the embedding distance, calculated as the cosine distance between location vectors modeled by word2vec, to test the ability to encode migration data. The decay function $f(r_{\mathrm{ij}})$ defines the effect of distance, and different decay functions can model fundamentally different mechanisms (56) such as the cost functions for a given distance and the spatial granularity of the observation. For geographic distance, we define $f(r_{\mathrm{ij}})$ as the standard power-law function, and for the embedding distance, we use the exponential function, selected as the best performing for each case (see *SI Appendix*, Figs. S10 and S11 for more information).

## Results

### word2vec and the Gravity Model.

We first demonstrate the mathematical equivalence between the SGNS model and the gravity model. The word2vec model takes a location trajectory, denoted by ($a_{1},a_{2},\ldots ,a_{T}$), as input. A target location $a_{t}=i$ is considered to have a context location $a_{t^{\prime }}=j$ that appears in the previous or subsequent w locations in the trajectory, i.e., $j\in [a_{t-w},\ldots ,a_{t-1},a_{t+1},\ldots ,a_{t+w}]$. word2vec learns an embedding by estimating the probability that location i has context j:

[2] $$\begin{array}{c} P\left(j|i\right):=\frac{\;\text{exp}(u_{j}\cdot v_{i})}{Z_{i}}, \end{array}$$

where the denominator $Z_{i}=\sum_{j^{\prime }\in A}\exp\left(u_{j^{\prime }}\cdot v_{i}\right)$ is a normalization constant, and A is the set of all locations. Although word2vec generates two embedding vectors $v_{i}$ and $u_{i}$—referred to as the in-vector and out-vector, respectively—we follow convention to use the in-vector $v_{i}$ as an embedding of location i. Training word2vec is computationally expensive because of $Z_{i}$ that extends over all |A| locations.

Negative sampling is a widely used heuristic to efficiently train word2vec without explicitly calculating $Z_{i}$. Negative sampling was introduced as a simplified version of Noise Contrastive Estimation (NCE) (8, 57). We show that this simplification gives rise to a biased estimator, which subsequently lead to the equivalence between SGNS word2vec and the gravity model.

NCE is a generic estimator for probability model (57)

[3] $$\begin{array}{c} P_{m}\left(x\right)=\frac{f(x)}{\sum_{x^{\prime }\in X}f\left(x^{\prime }\right)}, \end{array}$$

where f is a positive real-valued likelihood function of data x, and X is the set of all data. Note that word2vec belongs to this class of probability models, with $x=u_{i}\cdot v_{j}$ and f(x)=exp(x). To train word2vec with NCE (8), one samples a center-context pair (i,j) from the given data and labels the pair as Y=1. Then, one replaces the context location j with a random location $j^{\prime }$ sampled from a noise distribution $p_{0}\left(j^{\prime }\right)$ and labels the pair as Y=0. NCE finds the embedding that can classify the center-context pairs using a logistic function (*SI Appendix*)

[4] $$\begin{array}{c} P^{\text{NCE}}\left(Y_{j}=1|j\right)=\frac{1}{1+\exp\left[-\ln f\left(u_{j}\cdot v_{i}\right)+\ln p_{0}\left(j\right)\right]}, \end{array}$$

by maximizing the log-likelihood

[5] $$\begin{array}{cc} J^{\text{NCE}}= & \;\sum_{i\in A}\sum_{j\in D}\left[Y_{j}\log P^{\text{NCE}}\left(Y_{j}=1|j\right)\right.\\ & +\left.\left(1-Y_{j}\right)\log P^{\text{NCE}}\left(Y_{j}=0|j\right)\right]. \end{array}$$

Note that NCE is an unbiased estimator that has asymptomatic convergence to the optimal embedding in terms of the original word2vec’s objective function, J (57, 58). Let us revisit negative sampling from the perspective of NCE. Negative sampling simplifies NCE by dropping $\ln p_{0}\left(j\right)$ in the logistic function, i.e.,

[6] $$\begin{array}{c} P^{\text{NS}}\left(Y_{j}=1;v_{i},u_{j}\right)=\frac{1}{1+\exp(-u_{j}\cdot v_{i})}. \end{array}$$

Despite its innocuous appearance, this simplification produces substantial biases. To see this, we rewrite $P^{\text{NS}}$ in the form of $P^{\text{NCE}}$ as

[7] $$\begin{array}{cc} & P^{\text{NS}}\left(Y_{j}=1|j\right)\\ & =\frac{1}{1+\exp\left[-\left(u_{j}\cdot v_{i}+\ln p_{0}\left(j\right)+c\right)+\ln p_{0}\left(j\right)+c\right]}, \end{array}$$

[8] $$\begin{array}{cc} & =\frac{1}{1+\exp\left[-\ln f\left(u_{j}\cdot v_{i}\right)+\ln p_{0}\left(j\right)+c\right]}, \end{array}$$

where we define the likelihood function f by

[9] $$\begin{array}{c} f\left(u_{j}\cdot v_{i}\right)=\exp\left(u_{j}\cdot v_{i}+\ln p_{0}\left(j\right)+c\right), \end{array}$$

which is the unbiased estimator for the probability model

[10] $$\begin{array}{cc} P_{m}^{\text{NS}}\left(u_{j}\cdot v_{i}\right) & =\frac{f(u_{j}\cdot v_{i})}{\sum_{j^{\prime }\in A}f\left(u_{j^{\prime }}\cdot v_{i}\right)}, \end{array}$$

[11] $$\begin{array}{cc} & =\frac{p_{0}\left(j\right)\exp\left(u_{j}\cdot v_{i}\right)}{\sum_{j^{\prime }\in A}p_{0}\left(j^{\prime }\right)\exp\left(u_{j^{\prime }}\cdot v_{i}\right)}, \end{array}$$

[12] $$\begin{array}{cc} & =\frac{P^{\gamma }\left(j\right)\exp\left(u_{j}\cdot v_{i}\right)}{\sum_{j^{\prime }\in A}P^{\gamma }\left(j^{\prime }\right)\exp\left(u_{j^{\prime }}\cdot v_{i}\right)}\\ & (∵p_{0}\left(\ell \right)\propto P^{\gamma }\left(\ell \right)), \end{array}$$

where $P^{\gamma }\left(\ell \right)$ is the γ power of the frequency of the location ℓ.

Taken together, the conditional probability that SGNS word2vec actually optimizes is

[13] $$\begin{array}{c} P^{\text{NS}}\left(j|i\right)=P_{m}^{\text{NS}}\left(u_{j}\cdot v_{i}\right)=\frac{P^{\gamma }\left(j\right)\exp\left(u_{j}\cdot v_{i}\right)}{Z_{i}^{\prime }}, \end{array}$$

where $Z_{i}^{\prime }=\sum_{j^{\prime }\in A}P^{\gamma }\left(j^{\prime }\right)\exp\left(u_{j^{\prime }}\cdot v_{i}\right)$. (Eq. **13**) clarifies the bias due to negative sampling, i.e., the noise distribution $p_{0}\left(j\right)=P^{\gamma }\left(j\right)$ appears in the numerator and, thus, is a part of the word2vec model.

Armed with this result, we can now show the equivalence between the SGNS word2vec model and the gravity model. We set the window length to w=1 to restrict word2vec to predict the first-order flux ${\hat{T}}_{\mathrm{ij}}$ between locations, as is the case for the gravity model. Parameter γ=1 is a special choice that ensures that, when the embedding dimension is sufficiently large, there exists optimal in-vectors and out-vectors such that $v_{i}=u_{i}$ (43). By setting γ=1, we have

[14] $$\begin{array}{c} {\hat{T}}_{\mathrm{ij}}\propto P\left(i\right)P^{\text{NS}}\left(j|i\right)\propto \frac{P\left(i\right)P\left(j\right)\exp\left(u_{j}\cdot u_{i}\right)}{Z_{i}}. \end{array}$$

The flow ${\hat{T}}_{\mathrm{ij}}$ is symmetric (i.e., ${\hat{T}}_{\mathrm{ij}}={\hat{T}}_{\mathrm{ji}}$) because the skip-gram model neglects whether the context j appears before or after the target i in the trajectory which produces

[15] $$\begin{array}{cc} T_{\mathrm{ij}}=T_{\mathrm{ji}} & ⟺\frac{P\left(j\right)f\left(u_{j}\cdot u_{i}\right)}{Z_{i}}P\left(i\right)=\frac{P\left(i\right)f\left(u_{i}\cdot u_{j}\right)}{Z_{j}}P\left(j\right)\\ & ⟺\frac{1}{Z_{i}}=\frac{1}{Z_{j}}\\ & ⟺Z_{i}=Z_{j}, \end{array}$$

Taken together, the word2vec model with the negative sampling predicts a flow in the same form as the gravity model:

[16] $$\begin{array}{c} {\hat{T}}_{\mathrm{ij}}=CP\left(i\right)P\left(j\right)\exp\left(v_{j}\cdot v_{i}\right). \end{array}$$

In other words, with large-enough dimensions and embedding optimally converges, word2vec with skip-gram negative sampling is mathematically equivalent to the gravity model, with the mass given by the location’s frequency P(i), and the distance measured by their dot similarities. While the gravity model describes migration flows from the given mass and locations, word2vec estimates the positions in the vector space that best explain the given migration flow.

We further demonstrate word2vec’s capacity to effectively represent gravity-like relationships with a synthetic benchmark. Namely, we train a word2vec model using synthetic migration trajectories that strictly adhere to the gravity model (see *SI Appendix* for details). We find that distances in the embedding space strongly correlate with distances in the synthetic space which was explicitly structured according to the gravity model (Pearson correlation of 0.943 and 0.801, depending on the distance metric used; *SI Appendix*, Fig. S4). Our results align with a previous study about word2vec’ metric discovery capacity (45).

### Embeddings Provide Functional Distance between Locations.

To ensure that word2vec learns an accurate representation of migration that encodes meaningful functional distances, we devise an empirical validation task. Here, we expect that an accurate representation of the migration data should provide a functional distance that better models the flux between institutions than does geographic distance and other representation methods. We test this notion using three datasets representing different domains of human migration and mobility, showing that word2vec consistently offers a better representation of actual migration flows than geographic distance, or alternative network and direct optimization approaches.

In the case of scientific migration, the embedding distance explains more than twice the expected flux ($R^{2}=0.48$, Fig. 1*B*) than does the geographic distance ($R^{2}=0.22$, Fig. 1*C*), and predictions made using the embedding distance outperform those using the geographic distance (Fig. 1 *D* and *E*). These patterns hold for the subsets of only domestic (within-country organization pairs, *SI Appendix*, Figs. S10 and S12*C*) and only international migration flows (across-country organization pairs, *SI Appendix*, Fig. S12*D*). We also find that the embedding distance outperforms a generalized version of the gravity model, which incorporates information on shared geography and language alongside geographic distance (*SI Appendix*).

Similarly, the embedding distance explains more than twice the expected flux between airports ($R^{2}=0.51$, *SI Appendix*, Fig. S6*A*) than does geographic distance ($R^{2}=0.22$, *SI Appendix*, Fig. S6*B*), which has traditionally been used to quantify distance for the gravity model. Also, the embedding distance produces better predictions of actual flux between airports than does the geographic distance *SI Appendix*, Fig. S6 *C* and *D*). In the case of Korean accommodation reservations, embedding distance better explains the expected flux ($R^{2}=0.57$, *SI Appendix*, Fig. S6*E*) than does geographic distance ($R^{2}=0.25$, *SI Appendix*, Fig. S6*F*), and predictions made using the embedding distance outperform those made with geographic distance (*SI Appendix*, Fig. S6 *G* and *H*).

The embedding distance also out-performs alternative diffusion-based network distance measures including the personalized Page Rank scores calculated from the underlying migration network (*SI Appendix*, Figs. S8, S14, and S15). The embedding distance derived from neural embedding also explains more of the flux and better predicts migration flows than simpler embedding baselines, such as distances derived from a singular-value decomposition and a Laplacian Eigenmap embedding (59) of the underlying location co-occurrence matrix, Levy’s symmetric word2vec (43), and even direct optimization of the gravity model (*SI Appendix*, Fig. S8 and Tables S3–S5).

In sum, our results demonstrate that, consistently and efficiently, the embedding distance better captures patterns of actual migration than does the geographic distance. The embedding distance also outperforms alternatives in terms of the common part of commuters measure (60) (*SI Appendix*, Fig. S16).

In practice, because of noise, limited amounts of data, and imperfect optimization, the equivalence may only approximately hold. Indeed, we find that the in- and out-vectors tend to be different and that the cosine similarity tends to better capture real-world migration than the inner product similarity. This result echoes other applications of word embedding, such as word analogy testing (61), in which cosine distance also outperformed the inner product similarity. Nevertheless, a model with the inner product similarity has the second-best performance after cosine similarity (*SI Appendix*, Tables S3–S5), and the embedding distance still outperforms all alternatives we considered.

### Embeddings Capture the Global Structure of Migration.

**Fig. 2.**
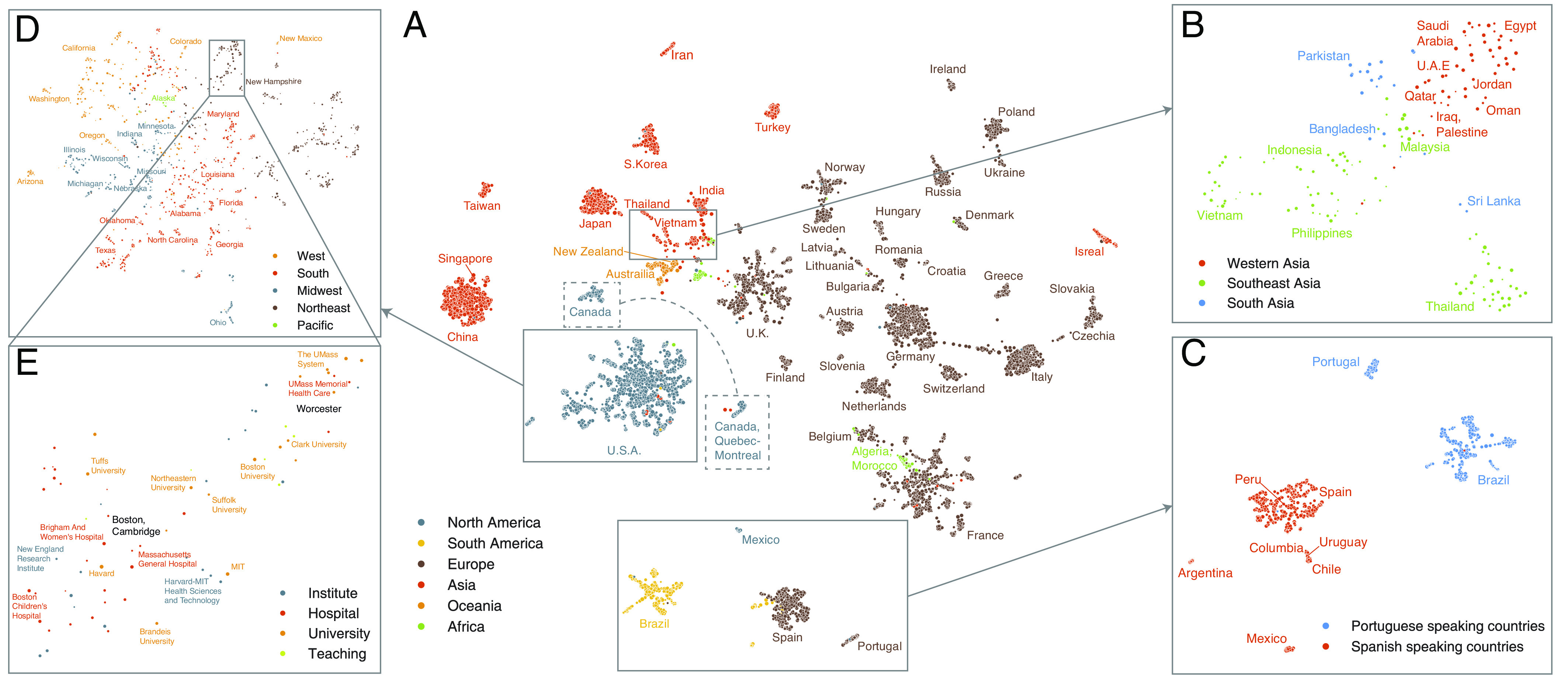
Projection of embedding space reveals complex multi-scale structure of organizations. (*A*) UMAP projection (62) of the embedding space reveals country-level clustering. Each point corresponds to an organization and its size indicates the average annual number of mobile and non-mobile authors affiliated with that organization from 2008 to 2019. Color indicates the region. The separation of organizations in Quebec and the rest of Canada is highlighted. (*B*) Zooming into (re-projecting) the area containing countries in Western, South, and Southeast Asia shows a geographic and cultural gradient of country clusters. (*C*) Similarly, zooming into the area containing organizations in Spain, Portugal, South, and Central America shows clustering by most widely spoken majority language group: Spanish and Portuguese. (*D*) Doing the same for organizations in the United States reveals geographic clustering based on state, roughly grouped by Census Bureau-designated regions, (*E*) Zooming in further on Massachusetts reveals clusters based on urban center (Boston, Worcester), organizational sector (hospitals vs. university), and university systems and prestige (UMass system vs. Harvard, MIT).

In the remainder of the paper, we focus on scientific migration as a case study to interrogate the geometric space generated by the neural embedding. In the process, we also study the multi-faceted relationships between scientific organizations. To explore the topological structure of the embedding, we use a topology-based dimensionality reduction method [UMAP (62)] to obtain a two-dimensional representation of the embedding space (Fig. 2*A*). By leveraging the unique characteristics of representation learning approach, we are able to show the relationships between individual organizations, rather than aggregates such as nations or cities, producing the largest and highest resolution “map” of scientific migration to date.

Globally, the geographic constraints are conspicuous; organizations tend to form clusters based on their national affiliations and national clusters tend to be near their geographic neighbors. At the same time, the embedding space also reflects a mix of geographic, historic, cultural, and linguistic relationships between regions much more clearly than alternative network representations (*SI Appendix*, Fig. S17) that have been common in studies of scientific migration (9, 63).

The embedding space also allows us to *zoom in* on subsets and re-project them to reveal local relationships. For example, re-projecting organizations located in Western, Southern, and Southeastern Asia with UMAP (Fig. 2*B*) reveals a gradient of countries between Egypt and the Philippines that largely corresponds to geography, but with some exceptions seemingly stemming from cultural and religious similarity. For example, Malaysia, with its official religion of Islam, is nearer to Middle Eastern countries in the embedding space than to many geographically closer South Asian countries. We validate this finding quantitatively with the cosine distance between nations (the centroids of organization vectors belonging to a given country). Malaysia is nearer to many Islamic countries such as Iraq (d=0.27), Pakistan (d=0.32), and Saudi Arabia (d=0.41) than neighboring but Buddhist Thailand (d=0.43) and neighboring Singapore (d=0.48).

Linguistic and historical ties also affect scientific migration. We observe that Spanish-speaking Latin American nations are positioned near Spain (Fig. 2*C*), rather than Portuguese-speaking Brazil (d=0.35 vs. d=0.54 for Mexico and d=0.39 vs. d=0.49 for Chile) reflecting linguistic and cultural ties. Similarly, North African countries that were once under French rule such as Morocco are closer to France (d=0.32) than to similarly geographically distant European countries such as Spain (d=0.39), Portugal (d=0.52), and Italy (d=0.52). Comparable patterns exist even within a single country. For example, organizations within Quebec in Canada are located nearer France (d=0.37) than the United States (d=0.51).

Mirroring the global pattern, organizations in the United States are largely arranged according to geography (Fig. 2*D*). Re-projecting organizations located in Massachusetts (Fig. 2*E*) reveals structure based on urban centers (Boston vs. Worcester), organization type (e.g., hospitals vs. universities), and university systems (University of Massachusetts system vs. Harvard and MIT). For example, even though UMass Boston is located in Boston, it clusters with other universities in the UMass System (d=0.29) rather than the other typically more highly ranked and research-focused organizations in Boston (d=0.39), implying a relative lack of migration between the two systems. Similar structures can be observed in other states such as among New York’s CUNY and SUNY systems (*SI Appendix*, Fig. S18), Pennsylvania’s state system (*SI Appendix*, Fig. S19), Texas’s Agricultural and Mechanical universities (*SI Appendix*, Fig. S20), and between the University of California and State University of California systems (*SI Appendix*, Fig. S21).

**Fig. 3.**
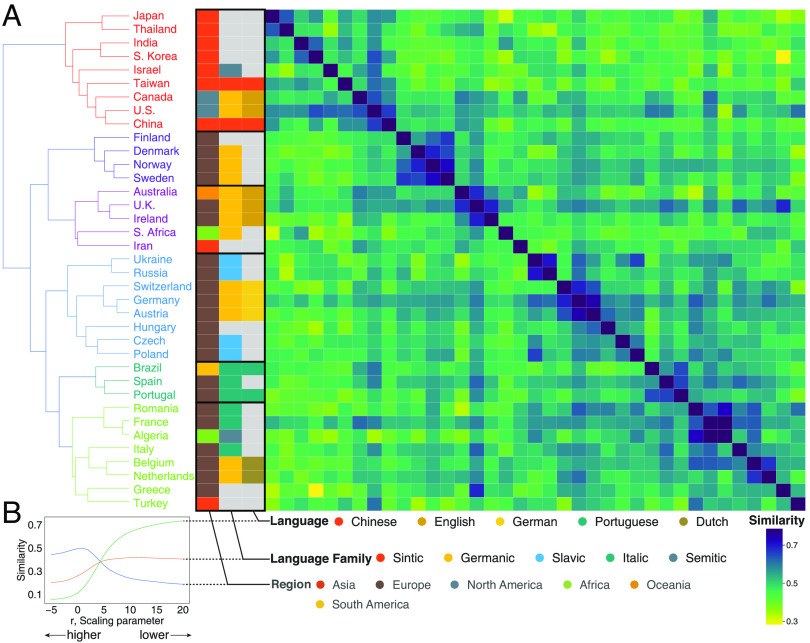
Geography, then language, conditions international migration. (*A*) Hierarchically clustered similarity matrix of country vectors aggregated as the mean of all organization vectors within countries with at least 25 organizations. Color of matrix cells corresponds to the cosine similarity between country vectors. Color of country names corresponds to their cluster. Color of three cell columns separated from the matrix corresponds to, from left to right, the region of the country, the language family (65), and the dominant language. (*B*) Element-centric cluster similarity (64) reveals the factors dictating hierarchical clustering (*Materials and Methods*). Region better explains the grouping of country vectors at higher levels of the clustering. Language family, and then the most widely spoken language, better explain the fine-grained grouping of countries.

Just as the embedding space makes it possible to *zoom in* on subsets of organizations, it is also possible to *zoom out* by aggregating organizational vectors. In doing so, we can examine the large-scale structure that governs scientific migration. We define the representative vector of each country as the average of their organizational vectors and, using their cosine similarities, perform hierarchical clustering of nations that have at least 25 organizations represented in the embedding space (Fig. 3*A*). The six identified clusters roughly correspond to countries in Asia and North America (orange), Northern Europe (dark blue), the British Commonwealth and Iran (purple), Central and Eastern Europe (light blue), South America and Iberia (dark green), and Western Europe and the Mediterranean (light green). The cluster structure shows that not only geography but also linguistic and cultural ties between countries are related to scientific migration.

We quantify the relative importance of geography (by region), and language (by the most widely spoken language of each country) using the element-centric clustering similarity (64), a method that can compare hierarchical clustering and disjoint clustering such as geography or language at the different levels of hierarchy by explicitly adjusting a scaling parameter r, acting like a zooming lens (*Materials and Methods*). If r is high, the similarity is based on the lower levels of the dendrogram, whereas when r is low, the similarity is based on higher levels. Fig. 3*B* demonstrates that regional relationships play a major role at higher levels of the clustering process (low r), and language (family) explains the clustering more at the lower levels (high r). This suggests that the embedding space captures the hierarchical structure of migration.

### Embeddings Capture Latent Prestige Hierarchy.

The embedding space can also encode more fine-grained relationships between entities. For example, prestige hierarchies, in which researchers tend to move to similar or less prestigious organizations (21, 22), are known to underpin the dynamics of scientific migration. Could the embedding space, to which no explicit prestige information is given, encode a prestige hierarchy? This question is tested by exploiting the geometric properties of the embedding space with SemAxis (44). Here, we use SemAxis to operationalize the abstract notion of academic prestige, defining an axis in the embedding space using known high- and low-ranked universities as poles. We use the Times Ranking of World Universities as an external proxy for prestige [we also use research impact from the Leiden Ranking (66); see *SI Appendix*], The high-rank pole is defined as the average vector of the top five U.S. universities according to the rankings, whereas the low-rank pole is defined using the five bottom-ranked (geographically matched by the U.S. census region) universities. We derive an embedding-based ranking for universities based on the geometrical spectrum from the high-ranked to low-ranked poles (*Materials and Methods*).

**Fig. 4.**
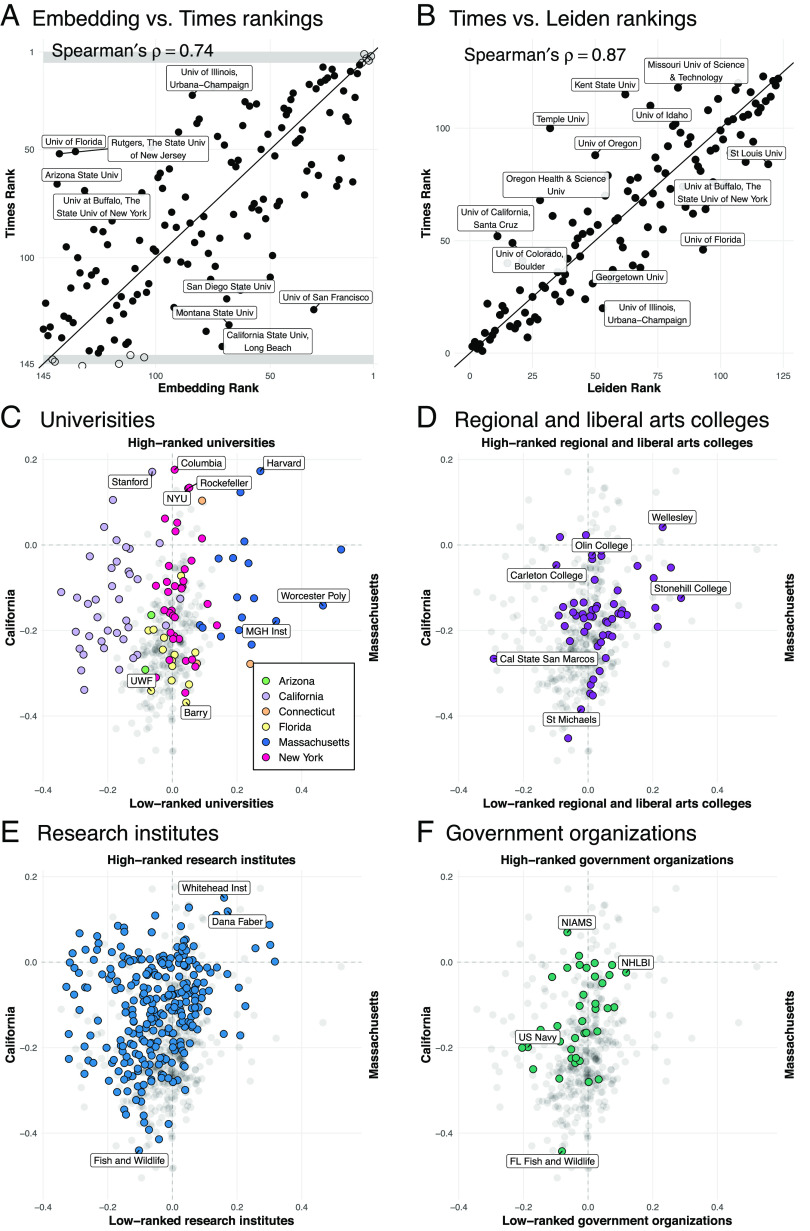
Embedding captures latent geography and prestige hierarchy. (*A*) Comparison between the ranking of organizations in the Times ranking and the embedding ranking derived using SemAxis. Un-filled points are those top and bottom five universities used to span the axis. Even when considering only a total of ten organization vectors, the estimate of the Spearman’s rank correlation between the embedding and Times ranking is ρ=0.73 (n=145, P<0.0001), which increases when more top-and-bottom ranked universities are included (*SI Appendix*, Fig. S22). (*B*) The Times ranking is correlated with Leiden Ranking of U.S. universities with Spearman’s ρ=0.87 and P<0.001. (*C*–*F*) Illustration of SemAxis projection along two axes; the *latent geographic axis*, from California to Massachusetts (left to right) and the *prestige axis*. Shown for U.S. Universities (*C*), Regional and liberal arts colleges (*D*), Research institutes (*E*), and Government organizations (*F*). Full organization names are listed in *SI Appendix*, Table S1.

The embedding space encodes the prestige hierarchy of U.S. universities that is coherent with real-world university rankings. The embedding-based ranking is strongly correlated with the Times ranking (Spearman’s ρ=0.73, Fig. 4*A*). They are also strongly correlated with the mean normalized citation score of university’s research output outlined in the Leiden rankings (66) (Spearman’s ρ=0.77, *SI Appendix*, Fig. S23*B*). For reference, the correlation between the Times and the Leiden rankings is 0.87 (Spearman’s ρ, Fig. 4*B*). The correlation between the embedding-based ranking and the Times ranking is robust regardless of the number of organizations used to define the axes (*SI Appendix*, Fig. S22), such that even using only the single top-ranked and bottom-ranked universities produces a ranking that is significantly correlated with the Times ranking (Spearman’s ρ=0.46, *SI Appendix*, Fig. S22). The correlation is also comparable to more direct measures such as node strength (sum of edge weights, Spearman’s ρ=0.73) and eigenvector centrality (Spearman’s ρ=0.76, see *SI Appendix*) from the migration network. The strongest outliers that were ranked more highly in the Times ranking than in the embedding-based ranking tend to be large state universities such as Arizona State University and the University of Florida. The institution higher in the embedding-based ranking tend to be relatively small universities near major urban areas such as the University of San Francisco and the University of Maryland Baltimore County, possibly reflecting exchanges of scholars with nearby highly ranked institutions at these locations. This analysis is not limited to the United States. Among the ten countries with the most universities represented in the Leiden rankings, all except for China have a Spearman’s ρ≥0.5 between their prestige axis and the relative rankings of their universities (*SI Appendix*, Table S6). In sum, our results suggest that the embedding space is capable of capturing information about academic prestige, even when the representation is learned using data without explicit information on the direction of migration [as in other formal models (21)], or prestige.

The axes can be visualized to examine the relative position of organizations along the prestige axis, and along a geographic axis between California and Massachusetts. Prestigious universities such as Columbia, Stanford, MIT, Harvard, and Rockefeller are positioned toward the top of the axis (Fig. 4*C*). Universities at the bottom of this axis tend to be regional universities with lower national profiles (yet still ranked by Times Higher Education) and with more emphasis on teaching, such as Barry University and California State University at Long Beach. The Massachusetts–California axis, roughly corresponding to East–West, further demonstrates the ability of these embeddings to capture latent geography. Distance along this axis strongly correlates with the longitudes of U.S. organizations (Spearman’s ρ=0.63).

By projecting other types of organizations onto the prestige axis, SemAxis offers a new way of representing a continuous spectrum of organizational prestige for which rankings are often low-resolution, incomplete, or entirely absent, such as for regional and liberal arts universities (Fig. 4*D*), research institutes (Fig. 4*E*), and government organizations (Fig. 4*F*). Their estimated prestige is speculative, though we find that it significantly correlates with their citation impact (*SI Appendix*, Fig. S27). Correlation with the geographic axis is strongest for universities (Spearman’s ρ=0.64), followed by research institutes (Spearman’s ρ=0.57) regional and liberal arts colleges (Spearman’s ρ=0.57), and government organizations (Spearman’s ρ=0.30); the relatively low geographic correlation for government organizations may stem from them having only one set of coordinates even if offices are spread across the country.

SemAxis rankings can also be applied toward investigating how prestige drives patterns of individuals’ migration (*SIAppendix*, Fig. S28). In line with past findings, we observe that transitions tend to occur between universities of similar or lower prestige (21). Additionally, we observe two clusters of internal migration at the top and bottom of the SemAxis hierarchy.

**Fig. 5.**
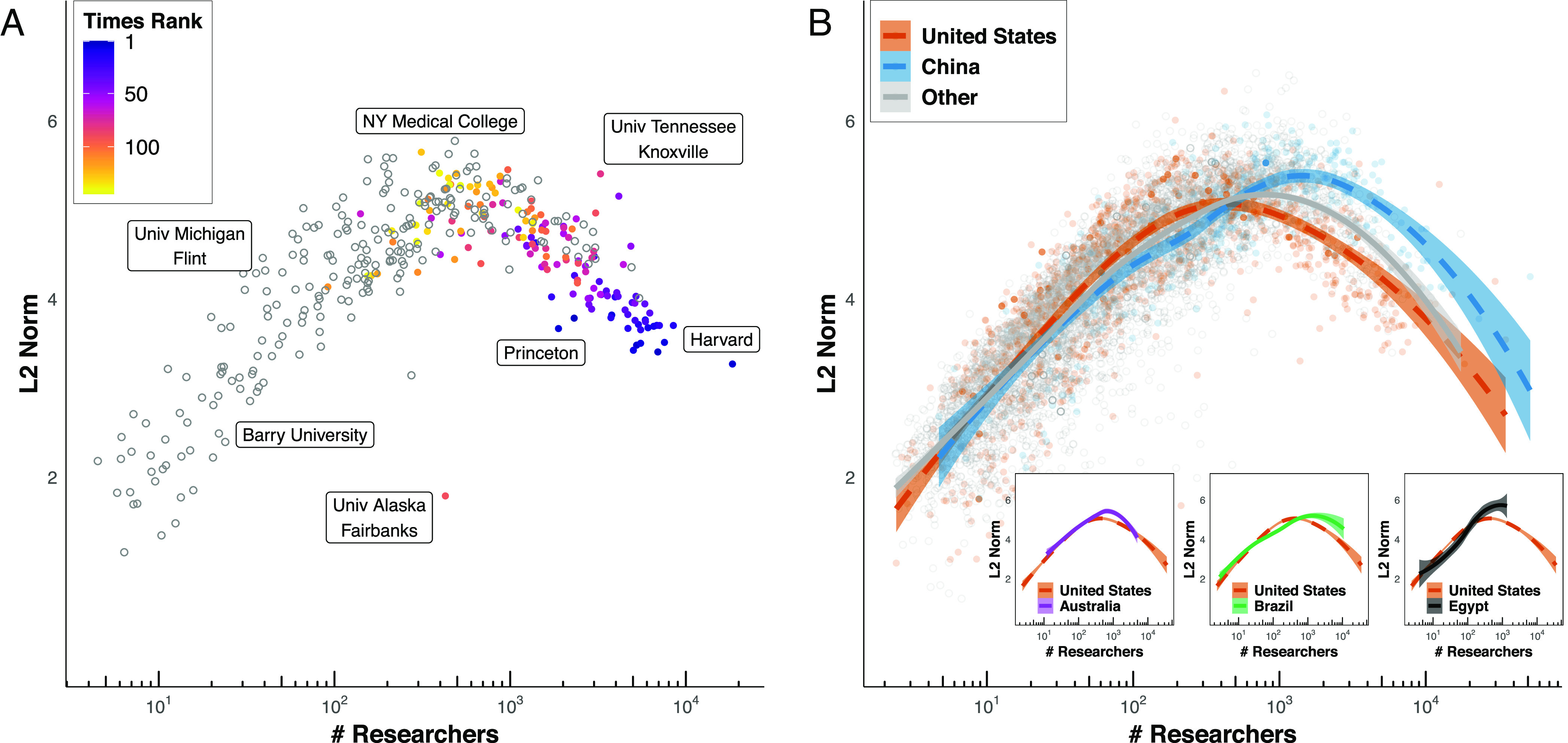
Size of organization embedding vectors captures prestige and size of organizations. (*A*) Size (L2 norm) of organization embedding vectors compared to the number of researchers for U.S. universities. Color indicates the rank of the university from the Times ranking, with 1 being the highest-ranked university. Uncolored points are universities not listed on the Times ranking. A concave shape emerges, wherein larger universities tend to be more distant from the origin (large L2 norm); however, the more prestigious universities tend to have smaller L2 norms. (*B*) We find a similar concave-curve pattern across many countries such as the United States, China, Australia, Brazil, and others (*Inset*, and *SI Appendix*, Fig. S30). Some countries exhibit variants of this pattern, such as Egypt, which is missing the right side of the curve. The loess regression lines are shown for each selected country, and for the aggregate of remaining countries, with ribbons mapping to the 99% CIs based on a normal distribution. Loess lines are also shown for organizations in Australia, Brazil, and Egypt (*Inset*).

We also find that the size (L2 norm) of the organization embedding vectors provides insights into the characteristics of organizations (Fig. 5). Up to a point (around 1,000 researchers), the size of U.S. organization’s vectors tends to increase proportionally to the number of researchers (both mobile and non-mobile) with published work; these organizations are primarily teaching-focused institutions, agencies, and hospitals that either are not ranked or have a low ranking. However, at around 1,000 researchers, the size of the vector decreases as the number of researchers increases. These organizations are primarily research-intensive and prestigious universities with higher rank, research outputs, R&D funding, and doctoral students (*SI Appendix*, Fig. S29). We report that this curve is almost universal across many countries. For instance, China’s curve closely mirrors that of the United States (Fig. 5*B*). Smaller but scientifically advanced countries such as Australia and other populous countries such as Brazil also exhibit curves similar to the United States (Fig. 5*B*, *Inset*). Other nations exhibit different curves which lack the portions with decreasing norm, probably indicating the lack of internationally prestigious institutions. Similar patterns can be found across many of the 30 countries with the most total researchers (*SI Appendix*, Fig. S30; see for more discussion).

A similar pattern has been observed in applications of neural embedding to natural language, where it was proposed that a word vector’s size represents its specificity, i.e., the word associated with the vector frequently co-appears with particular context words (67). If the word in question is universal, appearing frequently in many different contexts, it would not have a large norm due to a lack of strong association with a particular context. Under this view, an organization with a small norm, such as Harvard, appears in many contexts alongside many different organizations in affiliation trajectories—it is well connected. We conduct simple empirical and model-based investigations to verify the underlying dynamics of this curve pattern. However, in spite of theoretical support and an observed correlation between the vector size and the expected connectedness of the organization ($R^{2}=0.17$), these experiments do not support “specificity” as the sole mechanism of the observed concavity (*SI Appendix*). Another possibility is that the concave curve is a result of distortion caused by representing hierarchy in an Euclidean space (68), but this is also not supported by simulations. Instead, our findings emphasize that frequency and network connections constitute pivotal factors driving this pattern (*SI Appendix*, Figs. S32 and S33; see for more discussion). Further work is necessary to determine the exact causes of this curve pattern in so many countries, whether the same pattern can be found in other domains of human migration, and if they suggest common structures between both migration and language.

## Conclusion

Neural embedding approaches offer a novel, data-driven solution for efficiently learning an effective and robust representation of locations based on trajectory data, encoding the complex and multi-faceted nature of migration. We found that the unique strength of word2vec stems from its equivalence to a gravity model, making it a natural and theoretically grounded tool for modeling migration. By virtue of this equivalence, word2vec learns accurate representations of migration across disparate domains as we demonstrated here. Focusing on the case of scientific migration, we leverage the unique topological structure of the embedding space to reveal how it encodes nuanced aspects of migration, including global and regional geography, shared languages, and prestige hierarchies.

In revealing the correspondence between neural embeddings and the gravity model, the study of human migration can move beyond geographic and network-based models of migration, and instead leverage the high-order structure directly from individuals’ migration trajectories using these robust and efficient methods. This correspondence supplies a much-needed theoretical justification for the application of neural embedding techniques toward migration data and contributes to a better understanding of neural embedding techniques. Moreover, our study offers a complementary approach to past applications of neural networks toward migration and mobility data (49, 50). Whereas most location-based embeddings highlight their predictive capability, we instead illustrate how an embedding word2vec model creates accurate representations of migration data, a “digital double” that bundles many complex features of migration into a dense, continuous, and meaningful vector space representation. Using this representation, functional distances can be derived at multiple scales, but it can also be interrogated to reveal fundamental insights about migration. In addition to being intuitive, accessible, and theoretically grounded, the word2vec approach outlined here also has the advantage of learning complex and implicit features of migration directly from raw trajectory data, rather than exploiting a priori location features (51).

In conducting this analysis, we aim to offer a methodological framework for using word2vec to study scientific migration, and migration more broadly, such as animal migration, immigration trends, transit-network mobility, discretized cell-phone location data, and international trade. Once learned, functional distances between locations, such as countries, cities, or organizations, or the embedding model itself, can be published to facilitate re-use, and support reproducibility and transparency in cases when the underlying data are too sensitive to be made available. Moreover, this approach can be used to learn a functional distance even between entities for which no geographic analog exists, such as between occupational categories based on individuals’ career trajectories. In addition to providing a functional distance that supports modeling and predicting migration patterns, we also demonstrate, through a variety of unique and power techniques, how the semantic topology of the embedding space can be leveraged to facilitate interpretation and application of the complex features of migration. As we have shown, the embedding space allows the visualization of the complex structure of scientific migration at high resolution across multiple scales, providing a large and detailed map of the landscape of global scientific migration. Other operations such as comparing entities or calculating aggregates, which could be complex and computationally expensive for other methods, are here reduced to simple vector arithmetic. Embeddings also allow us to quantitatively explore abstract relationships between locations, such as academic prestige, and can potentially be generalized to other abstract axes. Investigation of the structure of the embedding space, such as the vector norm, reveals universal patterns based on the organization’s size and their vector norm that should be explored in future research.

This approach, and our study, also have several limitations. First, the skip-gram word2vec model assumes that migrations flows are symmetric, which is unlikely in real-world data. Breaking this assumption, however, also breaks the clear and simple mathematical equivalence between word2vec and the gravity model of migration. Future studies may consider directional embeddings to incorporate asymmetric nature of migration and mobility, such as the radiation model (4). Second, the neural embedding approach is most useful in cases of migration between discrete units such as between countries, cities, and businesses; this approach is less useful in the case of mobility between locations represented using geographic coordinates, such as that sourced from cell phone tracking. Third, neural embeddings are an inherently stochastic procedure, and so results may change across different iterations. However, in this study, we observe all results to be robust to stochasticity, likely emerging from the limited “vocabulary” of scientific mobility, airports, and accommodations (several thousand) and the relatively massive datasets used to learn representations (several million trajectories). Applications of word2vec to problem domains where the ratio of the vocabulary to data is smaller, however, should be implemented with caution to ensure that findings are not the result of random fluctuations. Fourth, the case of scientific migration presents domain-specific limitations. Reliance on bibliometric metadata means that we capture only long-term migration rather than the array of more frequent short-term mobility such as conference travel and temporary visits. The kinds of migration we do capture—migration and co-affiliation—although conceptually different, are treated identically by our model. Our data might further suffer from bias based on publication rates: researchers at prestigious organizations tend to have more publications, leading to these organizations appearing more frequently in affiliation trajectories. Fifth, for simplicity, we ignore the role of specialties. In line with the concept of a “persona” (69), it may be possible to create an interpretable embedding for each affiliation-subject pair, to understand the benefits associated with institutions that specialize in particular domains. Finally, our data are limited to the period between 2008 and 2019, and so may not reflect current patterns of migration that were shaped by the COVID-19 pandemic.

Migration and mobility are at the core of human nature and history, driving societal phenomena as diverse as epidemics (55, 70) and innovation (12–16). However, the paradigm of scientific migration may be changing. Traditional hubs of migration have experienced many politically motivated policy changes that affect scientific migration, such as travel restrictions in the United States and the United Kingdom (71), whereas other countries, such as China, have risen as major attractors of international talent (72). Unprecedented health crises such as the COVID-19 pandemic threaten to bring drastic global changes to migration by tightening borders and halting travel. By revealing the correspondence between neural embedding and the gravity model and revealing their utility and efficacy, our study provides a theoretical foundation and methodological framework for an approach that uses neural embeddings to study migration.

## Materials and Methods

### Scientific Migration Data.

We source co-affiliation trajectories of authors from the Web of Science database hosted by the Center for Science and Technology Studies at Leiden University. Trajectories are constructed from author affiliations listed on the byline of publications for an author. Given the limitations of author-name disambiguation, we limit our analyses to papers published after 2008, when the Web of Science began providing full names and institutional affiliations (73) that improved disambiguation (*SI Appendix*). This yields 33,934,672 author–affiliation combinations representing 12,963,792 authors. Each author–affiliation combination is associated with the publication year and an ID linking it to one of 8,661 disambiguated organizational affiliations (see *SI Appendix* for more detail). Trajectories are represented as the list of author–affiliation combinations, ordered by year of publication, and randomly ordered for combinations within the same year. The most fine-grained geographic unit in these data is the organization, such as a university, research institute, business, or government agency.

Here, authors are classified as mobile when they have at least two distinct organization IDs in their trajectory, meaning that they have published using two or more distinct affiliations between 2008 and 2019. Under this definition, mobile authors constitute 3,007,192 or 23.2% of all authors and 17,700,095 author–affiliation combinations. Mobile authors were associated with 2.5 distinct organizational affiliations on average. Rates of migration differ across countries. For example, France, Qatar, the USA, Iraq, and Luxembourg had the most mobile authors (*SI Appendix*, Fig. S2*C*). However, due to their size, the United States, accounted for nearly 40 % of all mobile authors worldwide (*SI Appendix*, Fig. S2*A*), with 10 countries accounting for 80 % of all migration (*SI Appendix*, Fig. S2*B*). The countries with the highest proportion of mobile scientists are France, Qatar, the United States, and Iraq, whereas those with the lowest are Jamaica, Serbia, Bosnia & Herzegovina, and North Macedonia (*SI Appendix*, Fig. S2*C*). In most cases, countries with a high degree of inter-organization migration also have a high degree of international migration, indicating that a high proportion of their total migration is international (*SI Appendix*, Fig. S2*D*). However, some countries such as France and the United States seem to have more domestic migration than international migration. While the number of publications has increased year-to-year, the migration and disciplinary makeup of the dataset have not notably changed across the period of study (*SI Appendix*, Fig. S1).

### U.S. Flight Itinerary Data.

We source U.S. airport itinerary data from the Origin and Destination Survey (DB1B), provided by the Bureau of Transportation Statistics at the United States Department of Transportation. DB1B is a sample of 10 percent of domestic airline tickets between 1993 and 2020, comprising 307,760,841 passenger itineraries between 828 U.S. airports. A trajectory is constructed for each passenger flight itinerary, forming an ordered sequence of unique identifiers of the origin and destination airports. Each itinerary is associated with a trajectory of airports including the origin, destination, and intermediary stops. We use population $m_{i}$ as the total number of unique passengers who passed through each airport.

### Korean Accommodation Reservation Data.

We source Korean accommodation reservation data from collaboration with Goodchoice Company Ltd. The data contain customer-level reservation trajectories spanning the period of August 2018 through July 2020 and comprising 1,038 unique accommodation locations in Seoul, South Korea. A trajectory is constructed for each customer, containing the ordered sequences of accommodations they reserved over time. We use the total number of unique customers who booked with each accommodation.

### Embedding.

We embed trajectories by treating them analogously to sentences and locations analogously to words. For U.S. airport itinerary data, trajectories are formed from the flight itineraries of individual passengers, in which airports correspond to unique identifiers. In the case of Korean accommodation reservations, trajectories comprise a sequence of accommodations reserved over a customer’s history. For scientific migration, an “affiliation trajectory” is constructed for each mobile author, which is built by concatenating together their ordered list of unique organization identifiers, as demonstrated in Fig. 1*A*, *Top*. In more complex cases, such as listing multiple affiliations on the same paper or publishing with different affiliations on multiple publications in the same year, the order is randomized within that year, as shown in Fig. 1 *A*, *Bottom*.

These trajectories are used as input to the standard skip-gram negative sampling word embedding, commonly known as word2vec (8). word2vec constructs dense and continuous vector representations of words and phrases, in which distance between words corresponds to a notion of semantic distance. By embedding trajectories, we aim to learn a dense vector for every location, for which the distance between vectors relates to the tendency for two locations to occur in similar contexts. Suppose a trajectory, denoted by ($a_{1},a_{2},\ldots ,a_{T}$), where $a_{t}$ is the tth location in the trajectory. A location, $a_{t}$, is considered to have context locations, $a_{t-w},\ldots ,a_{t-1},a_{t+1},\ldots ,a_{t+w}$, that appear in the window surrounding $a_{t}$ up to a time lag of w, where w is the window size parameter truncated at t−w≥0 and t+w≤T. Then, the model learns probability $p(a_{t+\tau }|a_{t})$, where −w≤τ≤w and τ≠0, by maximizing its log likelihood given by

[17] $$\begin{array}{c} J=\frac{1}{T}\sum_{t=1}^{T}\sum_{-w\leq \tau \leq w,\tau \neq 0}\log p\left(a_{t+\tau }|a_{t}\right), \end{array}$$

where,

[18] $$\begin{array}{c} p\left(j|i\right)=\frac{\exp(u_{j}\cdot v_{i})}{Z_{i}}, \end{array}$$

where v and u are the “in-vector” and “out-vector,” respectively, $Z_{i}=\sum_{j^{\prime }\in A}\exp\left(u_{j^{\prime }}\cdot v_{i}\right)$ is a normalization constant, and A is the set of all locations. We follow the standard practice and only use the in-vector, v, which is known to be superior to the out-vector in link prediction benchmarks (28–33, 36).

We used the word2vec implementation in the python package gensim. The skip-gram negative sampling word2vec model has several tunable hyper-parameters, including the embedding dimension d, the size of the context window w, the minimum frequency threshold $f_{\min}$, initial learning rate α, shape of negative sampling distribution γ, the number of the negative samples should be drawn k, and the number of iterations. For main results regarding scientific migration, we used d=300 and w=1, which were the parameters that best explained the flux between locations, though results were robust across different settings (*SI Appendix*, Fig. S7). Although the original word2vec paper uses γ=0.75(8), here we set γ=1.0, though results are only trivially different at different values of γ (*SI Appendix*, Fig. S8). We used k=5, which is suggested default of word2vec. We also use the same setting for U.S. airport itinerary and Korean accommodation reservation data.

To mitigate the effect of less common locations, we set $f_{\min}=50$, limiting to locations appearing at least 50 times across the training trajectories, resulting in embeddings reflecting 744 unique airports for the U.S. airport itinerary data, 1,004 unique accommodations for Korean accommodation reservation data, and 6,580 unique organizations for the scientific migration data. We set α to its default value of 0.025 and iterate five times over all training trajectories. For scientific migration, across each training iteration, the order of organizations within a single year is randomized to remove unclear sequential order.

### Distance.

We calculate $T_{\mathrm{ij}}$ as the total number of co-occurrences between two locations i and j across the dataset. In scientific migration, $T_{\mathrm{ij}}=10$ indicates that the number of co-occurrences between organizations i and j between 2008 and 2019 is 10, as evidenced from their publications. Here, we treat $T_{\mathrm{ij}}=T_{\mathrm{ji}}$ for the sake of simplicity and, in the case of scientific migration, because directionality cannot easily be derived from bibliometric records, or may not be particularly informative (*SI Appendix*).

We calculate two main forms of distance between locations. The geographic distance, $g_{\mathrm{ij}}$, is the pairwise geographic distance between locations. Geographic distance is calculated as the great circle distance, in kilometers, between pairs of locations. In the case of U.S. flight itinerary and scientific migration, we impute distance to 1 km when their distance is less than one kilometer. In the case of Korean accommodation reservation data, because this data represents trajectories of intra-city mobility that occurs at a much smaller scale international migration, we impute distance to 0.01 km when their distance is less than 0.01 km. The embedding distance with the cosine distance, $d_{\mathrm{ij}}$, is calculated as $d_{\mathrm{ij}}=1-\frac{v_{i}\cdot v_{j}}{\left|\right|v_{i}\left|||\right|v_{j}\left|\right|}$, where $v_{i}$ and $v_{j}$ are the embedding vectors for locations i and j, respectively. Note that $d_{\mathrm{ij}}$ is not a formal metric because it does not satisfy the triangle inequality. Nevertheless, cosine distance is often shown to be useful in practice (6, 7, 74). We compare the performance of this cosine-based embedding distance against those derived using inner product similarity and Euclidean distance.

We compare the performance of the embedding distance to many baselines. These include distances derived from simpler embedding approaches, such as Singular Value Decomposition (SVD) and a Laplacian Eigenmap embedding performed on the underlying location co-occurrence matrix. We also use network-based distances, calculating vectors using a Personalized Page Rank approach and measuring the distance between them using cosine distance and Jensen–Shannon divergence (*SI Appendix*). Finally, we compare the embedding distance against embeddings calculated through direct matrix factorization, following the approach that word2vec implicitly approximates (43).

### Gravity Law.

We model co-occurrences $T_{\mathrm{ij}}$ for locations i and j (referred to as flux), using the gravity law of mobility (3). The gravity law of mobility, which was inspired by Newton’s law of gravity, postulates that attraction between two locations is a function of their population and the distance between them. This formulation and variants have proven useful for modeling and predicting many kinds of migration and mobility (52–55). In the gravity law of mobility, the *expected flux*, ${\hat{T}}_{\mathrm{ij}}$ between two locations i and j is defined as,

[19] $$\begin{array}{c} {\hat{T}}_{\mathrm{ij}}=Cm_{i}m_{j}f\left(r_{\mathrm{ij}}\right), \end{array}$$

where $m_{i}$ and $m_{j}$ are the population of locations, defined as the total number of passengers who passed through each airport for U.S. airport itineraries, the total number of customers who booked with each accommodation for Korean accommodation reservations, and the yearly average count of unique authors, both mobile and non-mobile, affiliated with each organization for scientific migration. $f(r_{\mathrm{ij}})$ is a decay function of distance $r_{\mathrm{ij}}$ between locations i and j. Here, we used the most basic gravity model which assumes symmetry of the flow ${\hat{T}}_{\mathrm{ij}}={\hat{T}}_{\mathrm{ji}}$ and distance $r_{\mathrm{ij}}=r_{\mathrm{ji}}$, while there are four proposed variants (75). There are two popular forms for the $f(r_{\mathrm{ij}})$: One is a power law function in the form $f\left(r_{\mathrm{ij}}\right)=r_{\mathrm{ij}}^{-\alpha }\;\left(\alpha >0\right)$, and the other is an exponential function in the form $f\left(r_{\mathrm{ij}}\right)=e^{-\beta r_{\mathrm{ij}}}\;\left(\beta >0\right)$ (76). The parameters for $f(r_{\mathrm{ij}})$ and C are fit to given data using a log-linear regression (4, 52–55).

We consider separate variants of $f(r_{\mathrm{ij}})$ for the geographic distance, $g_{\mathrm{ij}}$, and the embedding distance, $d_{\mathrm{ij}}$, and report the best-fit model of each distance. For the geographic distance, we use the power-law function of the gravity law, $f\left(g_{\mathrm{ij}}\right)=g_{\mathrm{ij}}^{-\alpha }$ (Eq. **20**). For the embedding distance, we use the exponential function, with $f\left(d_{\mathrm{ij}}\right)=e^{-\beta d_{\mathrm{ij}}}$ (Eq. **21**).

[20] $$\begin{array}{c} \ln\frac{T_{\mathrm{ij}}}{m_{i}m_{j}}=\ln C-\alpha \ln g_{\mathrm{ij}}, \end{array}$$

[21] $$\begin{array}{c} \ln\frac{T_{\mathrm{ij}}}{m_{i}m_{j}}=\ln C-\beta d_{\mathrm{ij}}, \end{array}$$

where $T_{\mathrm{ij}}$ is the actual flow from the data. The gravity law of mobility is sensitive to $T_{\mathrm{ij}}=0$, or zero movement between locations. In our dataset, non-zero flows account for only 4.2% of all possible pairs of the 6,580 organizations for scientific migration, 76.4% of all possible pairs of the 744 airports for U.S. airport itinerary data, and 62.5% of all possible pairs of the 1,004 accommodations for Korean accommodation reservation data. This value is comparable to other common applications of the gravity law, such as phone calls, commuting, and migration (4). We follow standard practice and exclude zero flows from our analysis.

### Element-Centric Clustering Similarity.

Element-centric clustering similarity (64) is a similarity measure that can produce disjoint, overlapping, and hierarchically structured clusterings. Element-centric clustering similarity captures cluster-induced relationships between elements through a cluster affiliation graph where one vertex set is the original element $V=\{v_{1},...v_{N}\}$ and the other corresponds to the cluster $C=\{c_{1},...c_{M}\}$ as a bipartite graph B(V∪C,R). An undirected edge $a_{i\beta }\in R$ denotes element $v_{i}$ as a member of cluster $c_{\beta }$. For hierarchically structured clustering, each cluster $c_{\beta }$ is assigned a hierarchical level $l_{\beta }\in \left[0,1\right]$ by re-scaling the dendrogram according to the maximum path length from its roots. The weight of the cluster affiliation edge is given by the hierarchy weight function $a_{i\beta }=e^{rl_{\beta }}$ with scaling parameter r which determines the relative importance of membership at different levels of hierarchy. In this context, smaller r gives more importance to clusters that are closer to the root, prioritizing higher levels in the hierarchy. The lower levels are treated as a refinement of the higher level. Conversely, larger r places greater emphasis on the lower-level cluster structure, while viewing the higher levels of the hierarchy as an aggregation of the lower-level structure. When r=0, equal importance is assumed for every cluster.

The cluster affiliation graph is projected onto a cluster-induced element graph which is a weighted, directed graph summarizing the relationship induced by common cluster memberships. In the cluster-induced element graph, each edge between element $v_{i}$ and $v_{j}$ has weight $w_{\mathrm{ij}}=\sum_{\gamma }\frac{a_{i\gamma }a_{j\gamma }}{\sum_{\kappa }a_{i\kappa }\sum_{m}a_{m\gamma }}$. Given a cluster-induces element graph with weighted matrix W, the personalized PageRank vector $p_{i}$ is used as membership-aware similarity between element i and other elements in the graph. Then, the element-wise similarity of an element $v_{i}$ in two clusters A and B is calculated with $S_{i}\left(A,B\right)=1-L_{1}\left(p_{i}^{A},p_{j}^{B}\right)$, and the final element-centric similarity of two clustering A and B is found as the average of the element-wise similarities, $S\left(A,B\right)=\frac{1}{N}\sum_{i=1}^{N}S_{i}\left(A,B\right)$.

### SemAxis.

SemAxis and similar studies (30, 33, 44) demonstrated that “semantic axes” can be found from an embedding space by defining the “poles” and that the latent semantic relationship along the semantic axis can be extracted with simple arithmetic. In the case of natural language, the poles of the axis could be “good” and “bad,” “surprising” and “unsurprising,” or “masculine” and “feminine.” We can use SemAxis to leverage the semantic properties of the embedding vectors to operationalize abstract relationships between organizations.

Let $S^{+}=\left\{v_{1}^{+},v_{2}^{+}\cdots v_{n}^{+}\right\}$ and $S^{-}=\left\{v_{1}^{-},v_{2}^{-}\cdots v_{n}^{-}\right\}$ be the set of positive and negative pole organization vectors respectively. Then, the average vectors of each set can be calculated as $V^{+}=\frac{1}{n}\sum_{i=1}^{n}v_{i}^{+}$ and $V^{-}=\frac{1}{n}\sum_{i=1}^{n}v_{i}^{-}$. From these average vectors of each set of poles, the semantic axis is defined as $V_{\;\text{axis}}=V^{+}-V^{-}$. Then, a score of organization a is calculated as the cosine similarity of the organization’s vector with the axis,

[22] $$\begin{array}{c} \frac{v_{a}\cdot V_{\;\text{axis}}}{\left|\right|v_{a}\left|||\right|V_{\;\text{axis}}\left|\right|}, \end{array}$$

where a higher score for organization a indicates that a is more closely aligned to $V^{+}$ than $V^{-}$.

We define two axes to capture geography and academic prestige, respectively. The poles of the geographic axis are defined as the mean vector of all vectors corresponding to organizations in California and then the mean of all vectors of organizations in Massachusetts. For the prestige axis, we define a subset of top-ranked universities according to either the Times World University Ranking or based on the mean normalized research impact sourced from the Leiden Ranking. The other end of the prestige axis is the geographically matched (according to census region) set of universities ranked at the bottom of these rankings. For example, if 20 top-ranked universities are selected and six of them are in the Northeastern United States, then the bottom twenty will be chosen to also include six from the Northeastern United States. From the prestige axis, we derive a ranking of universities that we then compare to other formal university rankings using Spearman rank correlation.

## Supplementary Material

Appendix 01 (PDF)

## Data Availability

Anonymized data have been deposited in Figshare (https://doi.org/10.6084/m9.figshare.13072790.v1) (27).
