# Supplementary material for: Unsupervised embedding of trajectories captures the latent structure of scientific migration
Source: Proc Natl Acad Sci U S A. 2023 Dec 22;120(52):e2305414120. doi: 10.1073/pnas.2305414120 (PMC10756268; doi:10.1073/pnas.2305414120)
Supplement: Supplementary file 1 — Appendix 01 (PDF) [file pnas.2305414120.sapp.pdf]

Supporting Information: Unsupervised embedding of trajectories captures the latent structure of scientific migration

Dakota Murray, Jisung Yoon, Sadamori Kojaku, Rodrigo Costas, Woo-Sung Jung, Staša Milojević, Yong-Yeol Ahn

November 20, 2023

S1 Text Mobility and science.

As scholars move, they bring their knowledge, skills, and social connections with them—collectively the movements of researchers shape the structure and direction of the global scientific enterprise. For example, prestige-driven mobility between doctoral-granting and employing institutions is highly unequal[1,2], which affects the diffusion of ideas across academia[3]. By placing researchers in new social settings, mobility can lead to the formation of new collaborative relationships[4], which in turn spurs the further diffusion of knowledge and innovations[5–8]. Perhaps resulting from the selection effects of who gets to move, or the reconfiguring of social and epistemic networks, movement is associated with increased scientific impact[9–12]. At the national level, the understanding of mobility has progressed beyond simplistic narratives of brain drain and brain gain, and instead adopts a new perspective of *flows* of talent[13–15]. Under this flow model, a mobile researcher is viewed as contributing to both their origin and destination countries, a perspective that highlights the benefits of mobility to both the origin and destination countries. Perhaps because of these individual and national benefits, policy-makers have come to recognize the importance of global mobility[16,17]. Movement is a key mechanism that has clear impact on the composition and direction of the global scientific workforce and our collective scientific understanding. Understanding the structure and dynamics of mobility is thus essential for understanding global science.

S2 Text Modeling scientific mobility.

Modeling scientific mobility requires many considerations. First, the unit of analysis must be set. Most studies of mobility have focused on *country-level* mobility–the flows of researchers across nations[9,18–20]. Practically, country-level analyses benefit from higher reliability, such that idiosyncrasies and errors inherent to bibliographic databases are mitigated by this higher level of aggregation. Epistemically, country-level analysis is useful for national science governance which aims to understand the status of their country in the global landscape and make informed policy decisions. Analyses at lower units of analysis are far less common. *Regional*-level scientific mobility–the flow of researchers between regions or cities

within or across countries has been only minimally studied[21], possibly due to lack of reliable long-term data and lack of policy relevance to national-level lawmakers. *Organization*-level mobility has the potential to inform institutional policy and to understand the composition of mobility within a single country or region, especially as it relates to organization performance, prestige, and inequality[1–3,22]. However, affiliation disambiguation and noise in bibliographic data makes large-scale organization-level analysis challenging. Here, we learn neural-network embeddings of scientific mobility at the level of organizations using a curated bibliographic database. These embeddings are robust to noise, and so are capable of representing clear structure despite issues with organizational disambiguation. In doing so, embeddings also capture a more detailed understanding of mobility than has been previously studied.

Another consideration when analyzing scientific mobility is what kinds of mobility to study. Typical understandings of mobility are directional: movement is always *from* one place and *to* another. However, scientific mobility is more complicated. For example, scientists often hold multiple affiliations at a time[23], listing them as co-affiliations on a single paper, or even choosing a subset of affiliations to use across multiple simultaneous projects[24]. Even clearly-directional migration to another institution is complex–researchers may continue to publish with an old affiliation for projects that began before their move, and they may maintain social and organizational links to their old institution (e.g., collaborators, projects, graduate students) such that there is no clear breakage after migrating. There is also a whole range of short-term scientific mobility, such as visiting scholarships and short-term visits that are only visible through intensive efforts such as manual extraction from CVs[25–27]. Here, we focus on more long-term mobility that can be derived from bibliographic data. Due to the complexity of scientific mobility, we make the simplifying assumption that all scientific mobility is *symmetric* or without direction such that any move from an organization A to organization B is equivalent to a move from B to A. By assuming non-directional mobility, all mobility events are commensurate, meaning that they can be treated identically in our analysis–this allows us to represent the complexity of mobility without making decisions regarding the directionality of researchers' mobility or main affiliation. Moreover, this assumption has the practical advantage of matching the data format expected by the *word2vec* model, as well as the theoretical advantage of adhering to the symmetricity assumption of the gravity model of mobility.

S3 Text Building affiliation trajectories.

For each mobile researcher who has at least two distinct affiliations, we construct an affiliation trajectory based on the affiliations listed on their published papers indexed in the Web of Science database between 2008 and 2019. An author is considered mobile if they published with at least two distinct affiliations during the time period of study. Affiliation names were manually disambiguated, and each was mapped to a unique organization identifier. An affiliation trajectory for an individual researcher is a sequence of organizations in ascending order of year of publication. If a researcher published papers with affiliation A in year t, B in $t + 1$, C in $t + 2$ and A again in $t + 3$, then the affiliation trajectory is expressed as (A, B, C, A).

In the case that an individual lists multiple affiliations in a single year, affiliations listed

on publications published in that year are shuffled between each iteration of the *word2vec* training process (each epoch). For example, an author who published with affiliation A in t_0, and affiliations B and C in t_1 could appear as one of (A, B, C) or (A, C, B) in each training iteration. This effectively removes the effect of order within a year, as the order cannot be meaningfully established based on co-affiliations in a single paper, or on different affiliations listed on separate papers, for which the date of publication may not be representative of the actual completion of the project.

Other than restricting to only mobile researchers, we do not perform any filtering or reductions to affiliation trajectories. In the case that an author publishes with organization A four times in t_0, and affiliation B two times in t_1, their trajectory will be (A, A, A, A, B, B). Although mobile authors who publish more papers will have longer trajectories, *word2vec* will skip duplicate consecutive organization IDs, mitigating the impact of long repetitive trajectories.

S4 Text Demonstration on ability of *word2vec* to effectively represent gravity-like relationships with a simulation experiment

We conducted an additional experiment to test the ability of *word2vec* to recover the metric between locations, and to further demonstrate its equivalency with the gravity model. Specifically, we perform a synthetic benchmark to test whether *word2vec* can effectively recover gravity-like relationships in data that is known to contain them.

We start by generating synthetic mobility trajectories structured according to the gravity model. For this purpose, we first generate an artificial "geographic" space. 1,000 points are generated in a 2-dimensional space, with their coordinates distributed evenly between -1 and 1. These points act as the "locations" in our simulation. Each point is also assigned a "mass" randomly drawn from a power-law distribution $p(x) \propto x^{-k}$ (we use $k = 2$) that is meant to represent the "population" or "size" of each location.

With this simulated geography in place, we calculate the pairwise distance matrix $r_{ij}^{synthetic}$, which is then used to calculate a transition matrix between points, defined as $T_{ij} = M_i M_j f(r_{ij})$, with transition probabilities following the gravity law. The purpose of this transition matrix is to define the likelihood of movement between points (or *locations*) in our simulated geography based on their mass and pairwise distances. We conduct this experiment with two different transition matrices, one based on cosine distance where $f(r_{ij}) = e^{-\beta r_{ij}}$ and another with Euclidean distance where $f(r_{ij}) = r_{ij}^{-\alpha}$. For simplicity, we use $\beta = 2$ and $\alpha = 2$.

Finally, synthetic trajectories are generated based on the probabilities in the transition matrix. Specifically, we use a random walker based approach, where random walkers are initialized in locations with a probability proportional to their mass and transition between points according to the transition matrix. These synthetic trajectories are then used to train the skip-gram *word2vec* model. For parameters, we set window size to $w = 1$ and the embedding dimension to $d = 100$.

From the embedding trained with synthetic trajectories, we calculate the pairwise embedding distances between points, $r_{ij}^{embedding}$. To evaluate the ability of *word2vec* to recover the known gravity-like structure of the synthetic data, we compare the embedding distance to the distances between the 1,000 points in the synthetic space, $r_{ij}^{synthetic}$ using the Pearson correlation coefficient. This result is repeated 100 times, and the correlation coefficients are

aggregated across iterations.

We find that *word2vec* effectively recovers the gravity-like relationships in the synthetic data. Based on cosine distance, the Pearson correlation between the embedding and synthetic distances is 0.943, with a standard error of 0.01. Based on Euclidean distance, the correlation was 0.801, with a standard error of 0.01. To better illustrate this point, Fig. S4 shows the correlation for one representative iteration of this experiment.

In order to evaluate the robustness of the outcome across hyperparameter settings, we repeat our synthetic benchmark (using cosine distance) varying values for the mass exponent, k and distance exponent β. Fig. S5 presents the average Pearson correlation between the synthetic and embedding distance over ten iterations with for different values of k and β. This shows our findings to be robust. Neural embedding consistently recovers the distance from the synthetic trajectories across a broad range of hyperparameter values. A low correlation occurs only when the mass distribution is too heterogeneous ($k < 1.9$). In a heterogeneous mass distribution, most of the trajectories will concentrate on just a few high-mass locations, regardless of distance, which makes learning a distance challenging. Meanwhile, the exponent of the city population usually ranges between 2 and 3[28].

S5 Text Network-based personalized Page Rank distances.

We examine the gravity model on the Personalized Page Rank (PPR)[29] as a benchmark on the network. We construct the co-occurrence network of N organizations, in which each edge between organizations i and j represents a co-occurence of i and j in the same affiliation trajectory, with weight given by the sum of the co-occurences over all researchers. and edges are co-occurrence between two organizations. The Personalized Page Rank is a ranking algorithm for nodes based on a random walk process on networks. At each step, the walker visiting a node moves to a neighboring node chosen randomly with a probability proportional to the weight of the edge. Furthermore, with probability α, the walker is teleported back to the starting node. The rank of a node is determined by the probability that the walker visits the node in the stationary state. The stationary distribution of the random walker starting from node i, denoted by $p_i = (p_{ik})$, is given by

$$p_i = (1 - \alpha)W p_i + \alpha v_i \tag{1}$$

where v_i is a column vector of length N with entries that are all zero except the ith entry that equals one, $W = (W_{ij})$ is the weighted transition matrix of the unbiased random walker. We used $\alpha = 0.9$ here.

We can think of p_i as a representation vector of the organization i, and calculate the distance between organizations i and j, d_{ij}, with measuring distance between p_i and p_j to examine the gravity law. We consider two distance measures in this analysis. The first one is cosine distance which is used for our embedding method, $d_{ij} = 1 - \frac{\boldsymbol{p}_i \cdot \boldsymbol{p}_j}{\|\boldsymbol{p}_i\| \|\boldsymbol{p}_j\|}$. Also, if we think of p_i as a discrete probability distribution, then we can consider Jensen–Shannon divergence (JSD), which can be written as,

$$d_{ij} = JSD(p_i || p_j) = \frac{1}{2} D_{KL}(p_i || m) + \frac{1}{2} D_{KL}(p_j || m), \tag{2}$$

$$D_{KL}(p_i||m) = \sum^{x} p_{ix} \log \frac{p_{ix}}{m_x}, \tag{3}$$

where $m = \frac{1}{2}(p_i + p_j)$. We report the result with cosine distance ($R^2 = 0.14$, Fig. S14) and Jensen–Shannon divergence ($R^2 = 0.19$, Fig. S15). In both cases, the performance is under the performance of the model with geographical distance. Even though the length of the PPR vectors is much larger than the length of our embedding vectors, the result with the embedding distance outperforms both of them.

S6 Text Singular value decomposition distance.

We use the truncated singular value decomposition (SVD) on the underlying mobility co-occurrence matrix as a baseline embedding. In short, truncated SVD performs linear low-rank approximation of the matrix with given dimensions, d. First, we construct the co-occurrence matrix of N organizations, A_{ij} given by the co-occurrence of organizations i and j in the same affiliation trajectory. Then, we apply truncated singular value decomposition with $d = 300$ on the flow matrix A directly.

We calculate the distance between organizations in the SVD embedding space using cosine distance, finding that it explains slighly more of the flux between organizations than geographic distance ($R^2 = 0.247$, Table S3). When used as an input to the gravity model, this distance produces better predictions than geographic distance using both the exponential (RMSE = 0.859, Table S4) and power-law models (RMSE = 0.839, Table S4), performing slightly better with the power-law formulation.

S7 Text Laplacian Eigenmap distance.

We also consider Laplacian Eigenmap embeddings[30] as a baseline, which is one of the most fundamental approaches for graph embedding. First, we construct the co-occurrence matrix of N organizations, A_{ij} given by the co-occurrence of organizations i and j in the same affiliation trajectory and degree matrix D which is the diagonal matrix for which $D_{ii} = \sum_j A_{ij}$. Then we construct graph Lapalcian matrix $L = D - A$ and apply truncated singular value decomposition in the matrix L with $d = 300$.

We only report results based on the cosine distance between Laplacian embedding vectors, finding that it explains less of the total flux than geographic distance ($R^2 = 0.212$, Table S3). When used as an input to the gravity model, the Laplacian cosine distance produces marginally-better predictions than geographic distance using both the exponential (RMSE = 0.878, Table S4) and power-law models (RMSE = 0.87, Table S5), performing slightly better with the power-law formulation.

S8 Text Levy's symmetric SVD *word2vec* distance

We also compare the *word2vec* embedding distance against a baseline of direct matrix factorization approach, using the symmetric SVD *word2vec* method[31]. Based on notion that *word2vec* is equivalent to implicit matrix factorization, Levy proposed symmetric SVD *word2vec* embedding, which should directly compute the embedding that *word2vec* only attempts to efficiently approximate. First, we construct the matrix of N organizations

$$M_{ij} = \log\left(\frac{N(i,j)|D|}{N(i)N(j)}\right) - \log k, \tag{4}$$

where $N(i,j)$ is the number of times the location pair (i,j) appears given the window size w in the total corpus D, $N(i) = \sum_{j=N}^{i} N(i,j)$ as the number of items i occurred given the window size w in D, and k is the number of negative samples. We used $w = 1$ and $k = 5$ which is the same setting as in our main result. Then, we factorized matrix M with truncated singular value decomposition in the matrix with $d = 300$ into $U_d \Sigma_d V_d$, and used the embedding vector as $U_d \sqrt{\Sigma_d}$.

For this baseline, we report results using the cosine distance, Euclidean distance, and dot product between the embedding vectors. We find that the dot product performs by far the worst, worse than any other baseline considered $R^2 = 0.004$, Table S3). The cosine distance performs better, but worse than geographic distance ($R^2 = 0.212$, Table S3). The Euclidean distance performs best, explaining more of the flux than geographic distance, and only being below the embedding distance ($R^2 = 0.341$, Table S3). Focusing on the Euclidean distance, we find that using it as input to the gravity model results in better predictions than geographic distance using both the exponential model (RMSE = 0.803, Table S4) and power law models, (RMSE = 0.78, Table S5), though it performs slightly better with the power law formulation.

S9 Text Direct optimization of gravity model

Finally, we create an embedding by optimizing for the gravity law directly. Specifically, we construct a gravity matrix of N organizations, where each cell of the matrix is calculated as $T_{ij}/m_i m_j$, where T_{ij} is co-occurrence of organizations i and j in the same affiliation trajectory, and m_i and m_j are the organizations' populations, defined here as the mean annual number of unique mobile and non-mobile authors affiliated.

We then embed the gravity matrix using two approaches: a truncated singular value decomposition (SVD) and multidimensional scaling (MDS), which embeds each location in a N-dimensional space such that pairwise distances are preserved as well as possible. Typically, MDS uses Euclidean distance to measure distance between vectors.

For this analysis, we report results using the cosine distance between embedding vectors for the SVD embedding, and the logged Euclidean distance for the MDS embedding. We observe that the gravity-optimized SVD cosine distance has poor performance, more weakly correlated with actual flux than geographic distance ($R^2 = 0.122$, Table S3), and similarly poor prediction error when used as input to the gravity mode. In contrast, the cosine distance between MDS vectors has the second-highest correlation with actual flux, after only the embedding distance ($R^2 = 0.355$, Table S3), and third best for predicting actual flux using both the power-law version of the gravity model (RMSE = 0.795, Table S5) and the exponential form (RMSE = 0.904, Table S4). We note that the MDS embedding actually has the lowest error when organizations' populations are defined as the raw frequency when using the power-law model (RMSE = 0.691, Table S5). However, we note that the MDS distance is defined with a population of "all" mobile and non-mobile scholars, whereas the predictions made in Table S5 use the raw frequency, which likely confounds this result. MDS is also computationally-intensive, requiring upwards of 6 times more time to compute (on

the machine used in this analysis) than the more computationally efficient *word2vec* model.

S10 Text Why do neural embeddings outperform direct matrix factorization?

Why do neural-embedding approaches, which rely on stochastic gradient descent, outperform Levy's direct matrix factorization[31], especially given that *word2vec is* implicitly approximating factorization? We speculate that is stems, in part, from the sensitivity of matrix factorization to small flows between locations. Levy's matrix factorization embeds affiliations i and j such that their dot similarity is as close as possible to $\log(T_{ij}/P(i)P(j))$. If the flow T_{ij} is considerably small or zero, the dot similarity goes to $-\infty$, pushing i and j very far from other affiliations in the embedding space[31,32]. This is particularly problematic when the window size is small because most affiliation pairs would have no flow, which is indeed the case in our experiments. To circumvent this problem, previous studies[31,32] added a constant flow between the affiliation pairs with no flow. However, in addition to altering the underlying data, these small flows can still have a strong impact on the embedding.

It is also well known that singular value decomposition (SVD) is vulnerable to outliers[33–37]. The stochastic gradient descent algorithm, which is employed in SGNS *word2vec*, is more robust than SVD and can enhance generalization and effectiveness of *word2vec* model[38–40].

S11 Text Organization disambiguation and metadata.

Affiliations are mapped to one of 8,661 organizations and disambiguated following the procedure originally designed for the Leiden Rankings of World Universities[41]. Organizational records were associated with a full name, a type indicating the sector (e.g., University, Government, Industry), and an identifier for the country and city of the organization. Sixteen different sector types were included in the analysis, which we aggregated to four high-level codes: *University*, *Hospital*, *Government*, and *Other*. Each record was also associated with a latitude and longitude. However, for many organizations, these geographic coordinates were missing or incorrect. We manually updated the coordinates of 2,267 organizations by searching the institution name and city on Google Maps; in cases where a precise location of the organization could not be identified, we used the coordinates returned when searching the name of the city. The data was further enriched with country-level information, including region, most widely-spoken language, and its language family (e.g., the language family of *Spanish* is *Italic*). State/province-level information was added using the reverse geocoding service LocationIQ using each organization's latitude and longitude as input. Regional census classifications were added for states in the United States. For each organization, we calculated size as the average number of unique authors (mobile and non-mobile) who published with that organization across each year of our dataset; in the case that authors publish with multiple affiliations in a single year, they are counted towards each.

As a result of our disambiguation procedure, some affiliations are mapped to two organizations, one specific, and one more general. For example, any author affiliated with "Indiana University Bloomington" will also be listed as being affiliated with the "Indiana University System", a more general designation for all public universities in Indiana. However, a more general organization may not always occur alongside the more specific one. For example, a researcher affiliated with the smaller regional school "Indiana University South Bend" will

be listed as affiliated with only the "Indiana University System". We identify all specific organizations that always co-occur along with a more general one. For every career trajectory that includes one of these specific organizations, we remove all occurrences of the more general organization; trajectories containing only a general designation are not altered.

S12 Text Author name disambiguation.

Author-name disambiguation, the problem of associating names on papers with individuals authors, remains difficult for the use of bibliographic data[42]. Authors in our dataset have been disambiguated using a rule-based algorithm that makes use of author and paper metadata, such as physical addresses, co-authors, and journal, to score papers on the likelihood of belonging to an author cluster—a cluster of publications believed to have been authored by the same individual[43]. We limit our analysis to the period from 2008 to 2019, as in 2008 the Web of Science began indexing additional author-level metadata such as full names and email addresses. The disambiguation algorithm is conservative, favoring splitting clusters over merging. Past studies have validated this data and shown that the disambiguated authors are comparable to ground-truth records such as those from ORCID and useful for a wide range of bibliometric studies[9,24,44,45].

S13 Text Derivation of Eq. 5 - Noise Contrastive Estimation

The noise contrastive estimation (NCE)[46,47]. NCE is an unbiased estimator for a probability model P_m of the form:

$$P_m(x) = \frac{f(x)}{\sum_{x' \in \mathcal{X}} f(x')}, \tag{5}$$

where f is a non-negative likelihood function of data x, and $\mathcal{X}$ is the set of all data. This general form includes the *word2vec* model (Eq. 2), where $f(x) = \exp(x)$ and $x = \boldsymbol{u}_j \cdot \boldsymbol{v}_i$. NCE fits the probability model using a binary classification task in the same way as in negative sampling but using a Bayesian formalism for logistic regression. Specifically, before the training, we know that 1 in $1 + k$ words is sampled from the given data, which can be modeled as prior probabilities

$$P(Y_j = 1) = \frac{1}{k+1}, \quad P(Y_j = 0) = \frac{k}{k+1}. \tag{6}$$

Using the Bayes rule, the posterior probability for Y_j given word j is given by

$$P\left(Y_j|j\right) = \frac{P\left(j|Y_j\right) P(Y_j)}{P\left(j|Y_j = 0\right) P(Y_j = 0) + P\left(j|Y_j = 1\right) P(Y_j = 1)}. \tag{7}$$

Bearing in mind that word j is sampled from the given data if $Y_j = 1$ and from the noise distribution p_0 if $Y_j = 0$. Assuming that the given data is generated from the probability model to fit, the class-conditional probability, $P\left(j|Y_j\right)$, is given by

$$P(j|Y_j = 1) = P_m(\boldsymbol{u}_j \cdot \boldsymbol{v}_i), \quad P(j|Y_j = 0) = p_0(j). \tag{8}$$

Putting Eqs. (6), (7) and (8) together, the posterior probability for Y_j is given by

$$P\left(Y_j = 1|j\right) = \frac{P_m(\boldsymbol{u}_j \cdot \boldsymbol{v}_i)/(k+1)}{P_m(\boldsymbol{u}_j \cdot \boldsymbol{v}_i)/(k+1) + kp_0(j)/(k+1)} \tag{9}$$

$$= \frac{P_m(\boldsymbol{u}_j \cdot \boldsymbol{v}_i)}{P_m(\boldsymbol{u}_j \cdot \boldsymbol{v}_i) + kp_0(j)}, \tag{10}$$

which can be rewritten in form of sigmoid function:

$$P^{\mathrm{NCE}}\left(Y_j = 1|j\right) = = \frac{1}{1 + kp_0(j)/P_m(\boldsymbol{u}_j \cdot \boldsymbol{v}_i)} \tag{11}$$

$$= \frac{1}{1 + \exp\left[\ln kp_0(j) - \ln P_m(\boldsymbol{u}_j \cdot \boldsymbol{v}_i)\right]} \tag{12}$$

$$= \frac{1}{1 + \exp\left[-\ln f(\boldsymbol{u}_j \cdot \boldsymbol{v}_i) + \ln p_0(j) + c\right]}, \tag{13}$$

where $c = \ln k + \ln \sum_{x' \in \mathcal{X}} f(x')$ is a constant. NCE maximizes the log-likelihood

$$\mathcal{J}^{\mathrm{NCE}} = \sum_{i \in \mathcal{A}} \sum_{j \in \mathcal{D}} \left[Y_j \log P^{\mathrm{NCE}}(Y_j = 1|j) + (1 - Y_j) \log P^{\mathrm{NCE}}(Y_j = 0|j)\right]. \tag{14}$$

by calculating the gradients for embedding vectors $\boldsymbol{u}_j$, $\boldsymbol{v}_i$ and iteratively updating them. An important consequence of this framework is that NCE is an unbiased estimator that has convergence to the optimal embedding in terms of the original *word2vec's* objective function, $\mathcal{J}$ if we increase the number of words to sample and the training iterations[46,47].

S14 Text Reconstructing Times ranking with network measure.

The performance of the embedding ranking in reconstructing the Times ranking is comparable to that of network-derived measures such as degree strength (Spearman's $\rho = 0.73$, Fig. S24a) and eigenvector centrality (Spearman's $\rho = 0.76$, Fig. S24b). However, while both embedding- and network-based measures relate to university prestige, they are qualitatively and quantitatively different. The embedding-ranking of U.S. universities is less correlated with degree strength (Spearman's $\rho = 0.45$, Fig. S25a) and eigenvector centrality (Spearman's $\rho = 0.55$) than with the Times ranking itself (Spearman's $\rho = 0.73$, Fig. S25b). The embedding ranking over-ranks large research-intensive universities such as North Carolina State University, University of Florida, and Texas A&M University, whereas the network-derived ranking over-ranks smaller, more specialized universities such as Brandeis University, Yeshiva University, and University of San Francisco. This suggests that the embedding encodes information on prestige hierarchy at least as well as a network representation, with some noticeable qualitative differences.

S15 Text Speculation on variations of the concave-curve pattern.

The concave-curve pattern observed in Fig.5 repeats across many countries, with variations. We attempt to explain these variations through the lens of the "specificity" theory introduced in previous research[48]; that is, more "specific" terms appear in a limited contexts,

resulting in a longer vector, whereas more "universal" terms appear across many contexts, resulting in a short vector. We note that our own simulations (S16 Text) do not necessarily support this as the sole mechanism driving the concave-curve pattern, and so more work is needed to confirm and understand the factors underlying this pattern. Still, differences in the curve by country are consistent with the view that the length of a vector roughly corresponds to an organization's degree of specificity or universality.

To illustrate the country-level differences in average organizational vector length, the representative vector of Chinese organizations has a larger norm than that of the U.S. ($\bar{l} = 2.97$ vs $\bar{l} = 2.39$, Table S2), causing its curve to be shifted upwards with a larger peak vector norm; this may reflect a tendency for organizations in the U.S. to appear more frequently in different contexts than Chinese organizations. Other nations such as Poland, Iran, and Turkey show a linear relationship between an organization's number of researchers and the vector norm, indicating that their largest organizations belong to very specific contexts (Fig. S30). The organization-level distribution of vector norms reveals deeper heterogeneity. The distribution of the vector norms for the U.S. is relatively skewed, suggesting their large norm is driven by a small and tight community of organizations ($skew = -0.82$, Fig. S31). Germany and the U.K. have comparable representative vector norms to the U.S. ($\bar{l} = 2.6$ and $\bar{l} = 2.61$, respectively), with lower skewness ($skew = -0.63$ and $skew = -0.55$), suggesting more tight community of organizations. The vector norms of organizations in some countries are even more skewed, such as in Iran ($\bar{l} = 3.57$, $skew = -2.13$) and China ($\bar{l} = 2.97$, $skew = -1.08$), indicating the strong difference between their most- and least-connected organizations. For some countries, their organizations are positively-skewed, though seemingly for different reasons. For example, Austria has a balanced distribution of organization vector norms, suggesting a diverse range of organizations with most being well connected ($\bar{l} = 2.64$, $s = 0.18$); Russia, in contrast, has a number of organization vectors of moderate norms, but also several isolated organizations with large vector norms ($\bar{l} = 3.08$, $s = 0.67$).

S16 Text Experiment on the origin of the concave-curve pattern. We conduct a series of experiments to further investigate the concave-curve pattern between an organization's size (in total number of researchers) and the L2 norm of its embedding vector. We divide our explanation according to the two halves of the concave-curve: the "rising" portion, such that the vector L2 norm grows proportional to the size of an organization, and the "falling" portion, where the L2 norm begins to decline for larger organizations at a certain critical size.

We propose that the "rising" portion of the curve is directly related to the frequency with which an organization appears in the trajectory data. This phenomenon results from the *word2vec* training procedure. Before training begins, terms in the vocabulary (here, organization identifiers) are assigned random embedding vectors near the origin of the embedding space. During training, these vectors are iteratively updated based on information from each trajectory. Organizations that appear more often will be updated more frequently, stretching their embedding vector and thus their L2 norm. Organizations that appear rarely, however, will rarely be updated, and by the end of training will remain where they were instantiated near the origin of the embedding space, with short vectors. Thus, barring other circumstances, we should expect the size of an organization's L2 norm to correlate with their

frequency in the trajectories.

We support this argument with simulations based on synthetic trajectories which vary the prevalence of rare institutions. We start with the co-occurence network used in our manuscript, where nodes represent organizations and edges are weighted by the frequency of co-occurence of two organizations in the same trajectory. Synthetic trajectories are generated from this network by initializing random walkers at a node, and moving five steps, the average length in the real trajectory data, with each edge chosen with a probability proportional to its weight relative to all options. We generate two sets of synthetic data using this process, with the only difference being the initial placement of random walkers. The first set is generated by aligning the initial placement of walkers with that of individuals in the original data. The second set of synthetic data is generated by initializing random walkers as uniformly random across all organizations.

We anticipate that synthetic trajectories following the *first* process (random walkers initialized at each node proportional to their occurence in real trajectories) should produce the inverted "U" curvilinear pattern that we observe in our manuscript. Indeed, this is what we observe (Fig. S32a), implying that the process for generating synthetic data is producing trajectories that are an effective stand-in for real-world trajectory data. If our proposed mechanism for the rising trend is true, then we would expect the second set of synthetic trajectories (in which random walkers initialized at each node with uniform probability) to produce far more uniform L2 norms across organizations. Indeed, this is what we observe (Fig. S32b, note that the x-axis corresponds to size in the real data, ***not*** the synthetic data). Still, though, in Fig. S32b we observe a "falling" trend among larger institutions, which the existing mechanism does not account for, which we explore next.

Next we examine the "falling" portion of the curve. Previous research in natural language embedding [48] posits that groups of words confined to specific contexts often exhibit longer vectors, due to their continuously being updated in the same direction. Conversely, when a word is pervasive and appears frequently across diverse contexts, its vector norm is smaller, because each update pulls the vector in a different direction, leaving it near the origin of the embedding space. This phenomenon has also been observed in the field of chemistry [49] and has been recently explored in studies concerning the concept of specificity using L2-norm [50-52]. Alternative explanations are that the falling portion is a result of distortion caused by representing hierarchical structure in an Euclidean space, or that it is the result of some other process not outlined in previous literature.

We conduct a series of simulations in an attempt to obtain evidence in support or against these mechanisms. Synthetic trajectories are generated and embedded for a series of artificial networks, each network generated in an attempt to highlight a particular structural characteristic. The modeled networks are undirected, unweighted, and all consist of the same number of nodes as in the real co-occurence network of organizations used in our manuscript. Trajectories are generated on the network following the same random walker process as before, with starting positions sampled with a probability proportional to the node's degree. Below, we outline each generated network,

- Configuration model [53]: Random graph with the given degree sequence. We generate a configuration model network from the degree sequence of the mobility network. We define the size of each node as node degree.

- Regular tree: Regular tree is a representative model network with a strict hierarchical structure, characterized by m, the number of child nodes for each node, and d, the height of the tree. We generated the network with $m = 3$ and $d = 8$ consisting of $n = 9841$ nodes. Unlike the configuration model, the degree distribution of the tree network is nearly regular, since every node except the leaves and root have a comparable number of edges. In order to maintain the heterogeneity in the node sizes (i.e., number of scientists/authors in the nodes), we assign individual nodes the node sizes randomly sampled from the node size distribution for the original network.

- Barabási-Albert Model [54]: Model network with heterogeneous degree distributions using a preferential attachment mechanism. We set $m = 3$, the number of edges to attach from a new node to existing nodes. We define the size of each node as node degree.

- Perfectly nested network [55]: Simple nested model network without self-loop whose adjacency matrix is

$$A = \begin{bmatrix} 0 & 1 & 1 & .... & 1 \\ 1 & 0 & 1 & .... & 0 \\ . & . & . & .... & 0 \\ 1 & 1 & 0 & .... & 0 \\ 1 & 0 & 0 & .... & 0 \end{bmatrix}. \quad (15)$$

  In this network, a node with the largest degree connects to every node in the network, while a node with the smallest network only has a single connection. We define the size of each node as node degree.

The results derived from the model network are shown in Fig.S32. Remarkably similar concave shapes also manifest in the configuration model (Fig. S32a), the Barabási-Albert model (Fig. S32c), and the perfectly nested network (Fig. S32d). This strongly implies that the heterogeneous connection plays a crucial role in shaping these intriguing concave forms. In contrast, the regular tree (Fig. S32b), which solely embodies hierarchy, fails to yield such concave shapes. This observation underscores that the concave pattern doesn't emerge solely from distortion caused by embedding hierarchy into Euclidean spaces.

S17 Text Generalized gravity model.

Generalized gravity models use external information about each location to inform mobility predictions. Here, we test whether a generalized gravity model that includes information about shared city, region, nation, continent, and language alongside geographic distance offers better predictions than embedding distance. We specify the generalized model as follows,

$$\log \frac{T_{ij}}{M_i M_j} = \gamma \log d_{ij} + \sum_x \alpha^x * g^x_{ij} + \sum_x \beta^x * l^x_{ij} \quad (16)$$

where $g^x_{ij}, x \in$ [city, region, nation, and continent] is a dummy variable that is given a value of 1 when two organizations are located in the same geographic city, region, nation, or continent, respectively; otherwise, dummy variable is zero. Similarly, $l^x_{ij}, x \in$ [language, language family]

is a dummy variable that is set to 1 if two organizations are in countries that share the same language or language family, respectively.

Better results are obtained using the generalized gravity model (R^2 = 0.242, $RSME$ = 0.861, and CPC = 0.383) compared to standard gravity model using only geographical distance (R^2 = 0.219, $RSME$ = 0.874, and CPC = 0.373). However, the gravity model using embedding distance results in the best performance by a wide margin (R^2 = 0.482, $RSME$ = 0.711, and CPC = 0.459). These findings demonstrate how the embedding space can encode the complex and multi-faceted nature of mobility into a single robust vector-space representation, even without any external information provided.

Table S1: Full organization names

Short	Full	Short	Full
Stanford	Stanford Univ	Northwestern	Northwestern Univ
Columbia	Columbia Univ	Ball State	Ball State Univ
Harvard	Harvard Univ	IU Bloomington	Indiana Univ, Bloomington
UCLA	Univ of California, Los Angeles	Stevens Institute	Stevens Institute of Technology
Cal State Long Beach	California State Univ, Long Beach	NJIT	New Jersey Institute of Technology
Wright State	Wright State Univ	NYU	New York Univ
U Toledo	Univ of Toledo	SUNY Albany	Univ at Albany, The State Univ of New York
Boston U	Boston Univ	NY Medical College	New York Medical College
Suffolk	Suffolk Univ	Miami University	Miami Univ
CUNY	City Univ of New York (CUNY)	IU Pennsylvania	Indiana Univ of Pennsylvania
U Arizona	Univ of Arizona	Baylor	Baylor College of Medicine
OSU	Ohio State Univ	UT Health Center	Univ of Texas Health Science Center
MIT	Massachusetts Institute of Technology	Bard College	Bard College
Princeton	Princeton Univ	Stonehill College	Stonehill College
GCU	Grand Canyon Univ	Carleton College	Carleton College
Northcentral	Northcentral Univ	Hanover College	Hanover College
UCSF	Univ of California, San Francisco	Queens College	Queens College
Fielding	Fielding Graduate Univ	DePauw	DePauw College
Pepperdine	Pepperdine Univ	Naval Academy	United States Naval Academy
Argosy	Argosy Univ	Cal State San Marcos	California State Univ San Marcos
Yale	Yale Univ	Broad Inst	Broad Institute
U Hartford	Univ of Hartford	Forsyth Inst	Forsyth Institute
FAU	Florida Atlantic Univ	U Alaska Museum	Univ of Alaska Museum of the North
U Miami	Univ of Miami	Lawrence Berkeley	Lawrence Berkeley Natl Laboratory
UWF	The Univ of West Florida	Allen Institute	Allen Institute for Brain Science
FIT	Florida Institute of Technology	RTI International	RTI InterNatl
Purdue	Purdue Univ, West Lafayette	Fermilab	Fermilab
Notre Dame	Univ of Notre Dame	State of NY	State of New York
Indiana State	Indiana State Univ	Mayo Clinic	Mayo Clinic
Saint Mary's	Saint Mary's College	Fish and Wildlife	Fish and Wildlife Research Institute
Tufts	Tufts Univ	EPA	United States Environmental Protection Agency
Mattel	Mattel Children's Hospital	US Army	United States Army
Clark	Clark Univ	NSF	Natl Science Foundation
UMass Amherst	Univ of Massachusetts Amherst	US Navy	United States Navy
Montclair	Montclair State Univ	US Air Force	United States Air Force
Farleigh Dickinson	Fairleigh Dickinson Univ-Metro Campus	Ames Laboratory	Ames Laboratory
Rockefeller	Rockefeller Univ	Olin College	Oin College of Engineering
Adelphi	Adelphi Univ	Scrips Institute	Scrips Institute
Barnard	Barnard College	Idaho Natl Lab	Idaho Natl Laboratory
Saint John Fisher	Saint John Fisher College	Dana Faber	Dana Faber Cancer Institute
U Penn	Univ of Pennsylvania	Dept of Agriculture	United States Department of Agriculture
Villanova	Villanova Univ	DOE	United States Department of Energy
Widener	Widener Univ-Main Campus	NIAMS	Natl Institute of Arthritis, Skin Diseases
Robert Morris	Robert Morris Univ	JMI Labs	JMI Laboratories
U Cincinnati	Univ of Cincinnati	Whitehead Inst	Whitehead Institute of Biomedical Research
Case Western	Case Western Reserve Univ	Wellesley	Wellesley Univ
Ashland	Ashland Univ	UT Health, San Antonio	Univ of Texas Health Science Center, San Antonio
Texas A&M	Texas A&M Univ-Commerce	UNT	Univ of North Texas
Texas Southern	Texas Southern Univ	UT Southwestern Med	Univ of Texas Southwestern Medical Center
Baylor	Univ of Mary Hardin-Baylor	UT El Paso	Univ of Texas, El Paso
U Washington	Univ of Washington - Seattle	USF	Univ of South Florida, Tampa
Washington State	Washington State Univ	Florida A&M	Florida Agricultural and Mechanical Univ
Seattle Pacific	Seattle Pacific Univ	Barry	Barry Univ
Cal State Fresno	California State Univ-Fresno	UMass Dartmouth	Univ of Massachusetts Dartmouth
Northern Arizona	Northern Arizona Univ	Worcester Poly	Worcester Polytechnic Institute
IUPUI	Indiana Univ - Purdue Univ Indianapolis	Umass Boston	Univ of Massachusetts Boston
U Dayton	Univ of Dayton	MGH Inst	MGH Institute of Health Professions
U Conn	Univ of Connecticut	Joseph W. Jones Center	Joseph W. Jones Ecological Research Center
ASU	Arizona State Univ	Vaccine Research Center	Vaccine Research Center, San Diego
U Florida	Univ of Florida	LA Ag Center	Lousianna Agricultural Center
Northern Illinois	Northern Illinois Univ	FL Fish and Wildlife	Florida Fish and Wildlife Conservation Commission
Concordia Chicago	Concordia Univ-Chicago	NHLBI	Natl Heart, Lung, and Blood Institute
U Chicago	Univ of Chicago	NY Dept. of Health	New York Department of Health
SIU Edwardsville	Southern Illinois Univ, Edwardsville	St Michaels	Saint Michaels College
SIU Carbondale	Southern Illinois Univ, Carbondale		

Table S2: L2 Norm of country's representative vectors. Shown for top 30 countries with the most unique mobile and non-mobile researchers

**Country**	**L2 Norm**	**# Organizations**
United States	2.39	1281
Germany	2.6	485
United Kingdom	2.61	514
Austria	2.64	74
France	2.83	688
Belgium	2.84	84
Switzerland	2.85	66
Spain	2.94	322
China	2.97	497
India	2.99	114
Poland	3.02	145
Canada	3.02	147
Italy	3.04	386
Russia	3.08	187
Norway	3.1	122
Netherlands	3.11	136
Sweden	3.16	75
Brazil	3.16	286
Finland	3.17	66
Denmark	3.21	54
Czech Republic	3.23	97
Greece	3.24	62
Australia	3.24	90
Turkey	3.28	99
South Korea	3.28	156
Israel	3.32	71
Portugal	3.33	57
Japan	3.35	465
Iran	3.57	68
Taiwan	3.67	72

Table S3: Correlation between flux and distance over metrics, experimental parameters. Each cell corresponds to the correlation between the real-world flux between scientific organizations (measured with R^2) and baseline metrics, shown by subsets of mobility data and by definitions of organization population. The asterisk denotes the top-performing distance metric by column. Distance metrics are ordered from highest R^2 to lowest, based on global mobility with organization population defined using all mobile and non-mobile authors. "All" means that population is defined as the average yearly number of unique mobile and non-mobile scholars who published with the organizations' affiliation; population is defined in the same way for "Mobile only", except only using unique mobile researchers; "Raw freq" means that organization populations are defined as their frequency across all the trajectories, similar to word frequency in language embedding. Embedding distance, measured as the cosine distance between embedding vectors, explains more of the flux than baselines in nearly every case, except using raw frequency population and domestic and international mobility, where direct optimization of the gravity model works better, as well as Levy's factorization[31] for domestic and international only mobility.

	**All**			**Mobile only**			**Raw freq**		
	**All**	**Domestic**	**Inter.**	**All**	**Domestic**	**Inter.**	**All**	**Domestic**	**Inter.**
**Embedding cosine**	*0.481	*0.418	*0.435	*0.492	*0.456	*0.489	0.325	0.251	0.252
**Gravity MDS Euclidean**	0.355	0.165	0.161	0.328	0.112	0.115	*0.369	0.174	0.164
**Levy's Euclidean**	0.341	0.369	0.382	0.213	0.271	0.284	0.305	*0.323	*0.334
**Embedding dot**	0.341	0.313	0.316	0.254	0.265	0.267	0.218	0.181	0.177
**SVD cosine**	0.247	0.297	0.309	0.213	0.314	0.325	0.111	0.152	0.16
**Geographic**	0.219	0.174	0.197	0.188	0.157	0.176	0.04	0.019	0.03
**Laplacian cosine**	0.212	0.199	0.218	0.176	0.157	0.18	0.079	0.1	0.111
**Levy's cosine**	0.208	0.227	0.231	0.169	0.246	0.246	0.057	0.054	0.053
**PPR JSD**	0.194	0.276	0.276	0.218	0.335	0.335	0.012	0.077	0.069
**PPR cosine**	0.138	0.136	0.143	0.196	0.186	0.197	0.13	0.149	0.152
**Gravity SVD cosine**	0.122	0.118	0.122	0.118	0.133	0.138	0.056	0.047	0.05
**Levy's dot**	0.004	0.002	0.002	0.013	0.002	0.004	0.039	0.034	0.035

Table S4: Prediction error between actual and predicted mobility with exponential gravity model, by metrics and experimental parameters. Each cell corresponds to the prediction error (measured with root mean squared error) when using each distance as input to the exponential form of the gravity model of mobility to predict the flux between organizations, shown by subsets of mobility data, and by definitions of organization population. The asterisk denotes the top-performing distance metric by column (lowest prediction error). Distance metrics are ordered from lowest prediction error to highest, based on global mobility with organization population defined using all mobile and non-mobile authors. "All" means that population is defined as the average yearly number of unique mobile and non-mobile scholars who published with the organizations' affiliation; population is defined in the same way for "Mobile only", except only using unique mobile researchers; "Raw freq" means that organization populations are defined as their frequency across all the trajectories, similar to word frequency in language embedding. Embedding distance, measured as the cosine distance between embedding vectors, results in better predictions of mobility than baselines in nearly every case, however Levy's 'factorization[31] perform better in the case of international and domestic only mobility when using raw frequency populations.

	**All**			**Mobile only**			**Raw freq**		
	**All**	**Domestic**	**Inter.**	**All**	**Domestic**	**Inter.**	**All**	**Domestic**	**Inter.**
**Embedding cosine**	*0.713	*0.76	*0.737	*0.702	*0.749	*0.713	*0.715	0.764	0.748
**Levy's Euclidean**	0.803	0.791	0.771	0.874	0.867	0.844	0.725	*0.726	*0.705
**Embedding dot**	0.803	0.825	0.811	0.851	0.87	0.854	0.769	0.798	0.784
**SVD cosine**	0.859	0.835	0.815	0.874	0.841	0.82	0.82	0.812	0.792
**Laplacian cosine**	0.878	0.891	0.867	0.894	0.933	0.904	0.835	0.837	0.815
**Levy's cosine**	0.881	0.875	0.86	0.898	0.882	0.866	0.844	0.858	0.841
**PPR JSD**	0.888	7	0.835	0.871	0.828	0.814	0.865	0.847	0.834
**Gravity MDS Euclidean**	0.904	0.944	0.93	0.907	0.972	0.955	0.793	0.838	0.821
**PPR cosine**	0.918	0.925	0.908	0.884	0.916	0.894	0.812	0.814	0.796
**Geographic**	0.929	0.951	0.928	0.973	1.002	0.983	0.856	0.875	0.853
**Gravity SVD cosine**	0.933	0.939	0.923	0.92	0.946	0.927	0.853	0.865	0.847
**Levy's dot**	0.987	0.995	0.98	0.979	1.014	0.996	0.853	0.867	0.849

Table S5: Prediction error between actual and predicted mobility with power-law gravity model, by metrics and experimental parameters. Each cell corresponds to the prediction error (measured with root mean squared error) when using each distance as input to the power-law form of the gravity model of mobility to predict the flux between organizations, shown by subsets of mobility data, and by definitions of organization population. The asterisk denotes the top-performing distance metric by column (lowest prediction error). Distance metrics are ordered from lowest prediction error to highest, based on global mobility with organization population defined using all mobile and non-mobile authors. "All" means that population is defined as the average yearly number of unique mobile and non-mobile scholars who published with the organizations' affiliation; population is defined in the same way for "Mobile only", except only using unique mobile researchers; "Raw freq" means that organization populations are defined as their frequency across all the trajectories, similar to word frequency in language embedding. Embedding distance, measured as the cosine distance between embedding vectors, results in better predictions for global mobility with "All'; and "Mobile only" definitions of population, though direvt gravity-law opimization with MDS performs better when using raw frequencies to measure organization population, and Levy's factorization[31] perform better here with domestic and international only mobility.

	All			Mobile only			Raw freq		
	**All**	**Domestic**	**Inter.**	**All**	**Domestic**	**Inter.**	**All**	**Domestic**	**Inter.**
**Embedding cosine**	*0.743	0.784	0.76	*0.73	*0.784	*0.749	0.714	0.762	0.745
**Levy's Euclidean**	0.78	*0.776	*0.755	0.844	0.847	0.822	0.703	*0.711	*0.691
**Gravity MDS Euclidean**	0.795	0.91	0.898	0.808	0.957	0.939	*0.691	0.802	0.79
**Embedding dot**	0.822	0.844	0.831	0.864	0.879	0.863	0.783	0.809	0.795
**SVD cosine**	0.839	0.785	0.761	0.89	0.834	0.81	0.792	0.752	0.728
**Laplacian cosine**	0.87	0.837	0.801	0.937	0.919	0.886	0.804	0.772	0.735
**Geographic**	0.874	0.905	0.879	0.888	0.933	0.906	0.852	0.874	0.851
**PPR JSD**	0.889	0.85	0.838	0.871	0.834	0.819	0.865	0.847	0.834
**PPR cosine**	0.92	0.927	0.91	0.885	0.918	0.896	0.812	0.815	0.797
**Levy's cosine**	0.927	0.926	0.911	0.93	0.924	0.909	0.861	0.872	0.854
**Gravity SVD cosine**	0.965	0.965	0.95	0.955	0.958	0.941	0.873	0.883	0.866
**Levy's dot**	0.99	0.998	0.983	0.977	1.015	0.997	0.845	0.86	0.841

Table S6: SemAxis captures prestige across multiple countries. The Spearman's ρ between the prestige axis generated by SemAxis and the ranking of universities according to their fractional mean normalized citation score in the Leiden rankings[41]. Shown for the top ten countries by number of universities represented in the Leiden rankings. The prestige axis is generated for each country using their top and bottom 5 ranked universities. We note that the lowest value, $\rho = 0.458$ is for China, which may be a consequence of the difficulty in disambiguating the names of authors with Chinese names.

Country	Count of orgs	Spearman's ρ
USA	172	0.763
CHN	165	0.458
DEU	50	0.601
GBR	45	0.915
JPN	41	0.651
ITA	40	0.543
KOR	35	0.756
ESP	34	0.889
CAN	27	0.761
AUS	26	0.718

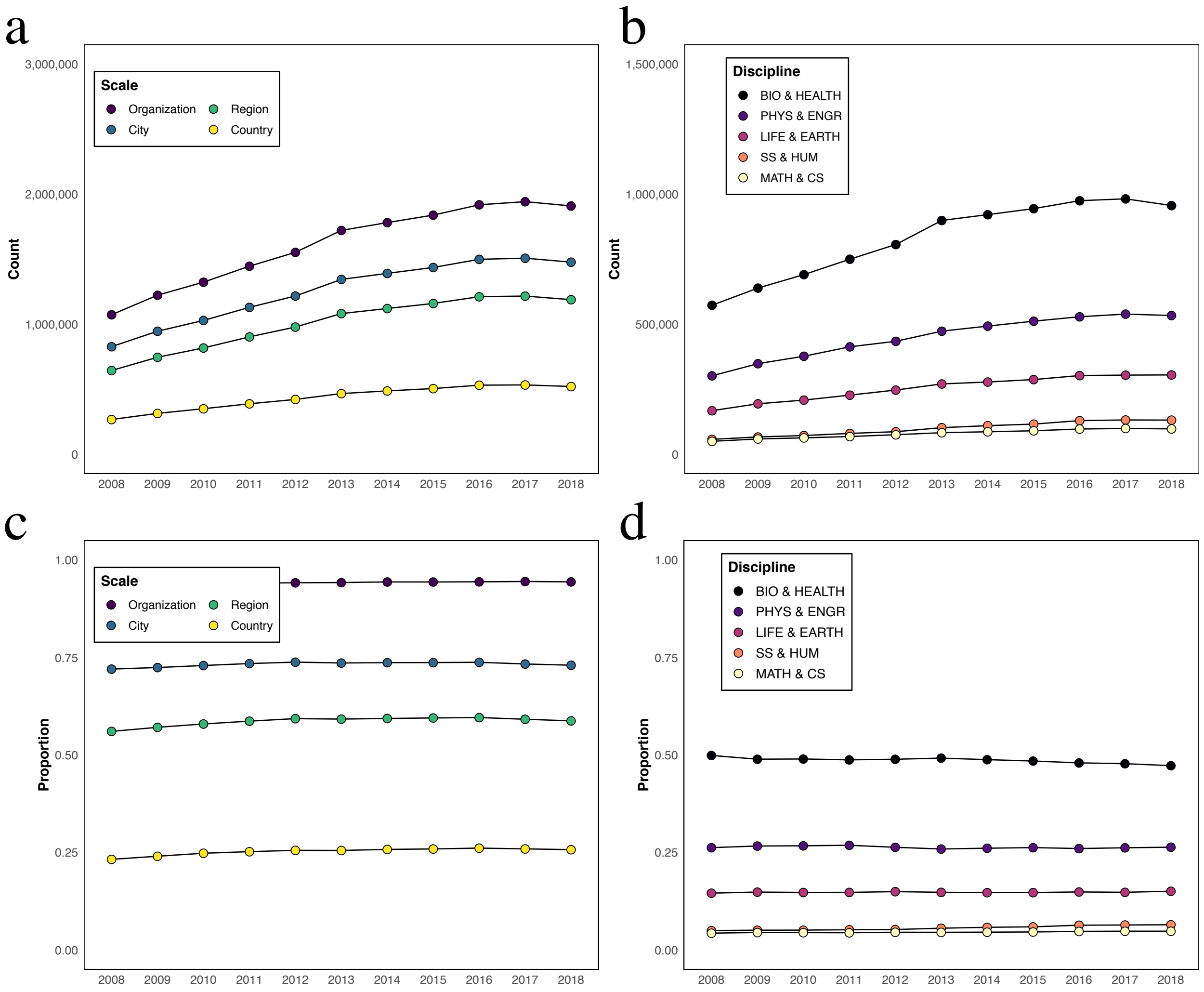

Figure S1: Publications over time. **a.** The number of papers published by mobile authors has been steadily increasing from 2008 to 2017, with a small decrease in 2018, which may be due to an artifact of the Web of Science indexing process. Lines correspond to publications by mobile authors, by authors with affiliations in at least two cities, at least two regions, and at least two countries. We did not find major changes in the publication patterns of mobile authors during this time period. **b.** Lines correspond to the proportion of publications classified as Biology and Health (black), Physics and Engineering (purple), Life and Earth Science (magenta), Social Science and Humanities (orange), and Math and Computer Science (yellow). The rate of publication in Biology and Health has leveled since about 2013, whereas the rate of publication in other fields has steadily increased. **c.** While the absolute count of publications has increased, the percentage of mobile scholars, and those with affiliations in at least least two cities, regions, or countries, as a proportion of all publications, has remained stable over time. **d.** The proportion of authors' publications across fields has largely remained steady. Biology and Health Science has comprised the majority of publications across nearly all years but has steadily declined in proportion. However, the proportion of Social Science and Humanities publications has been steadily increasing.

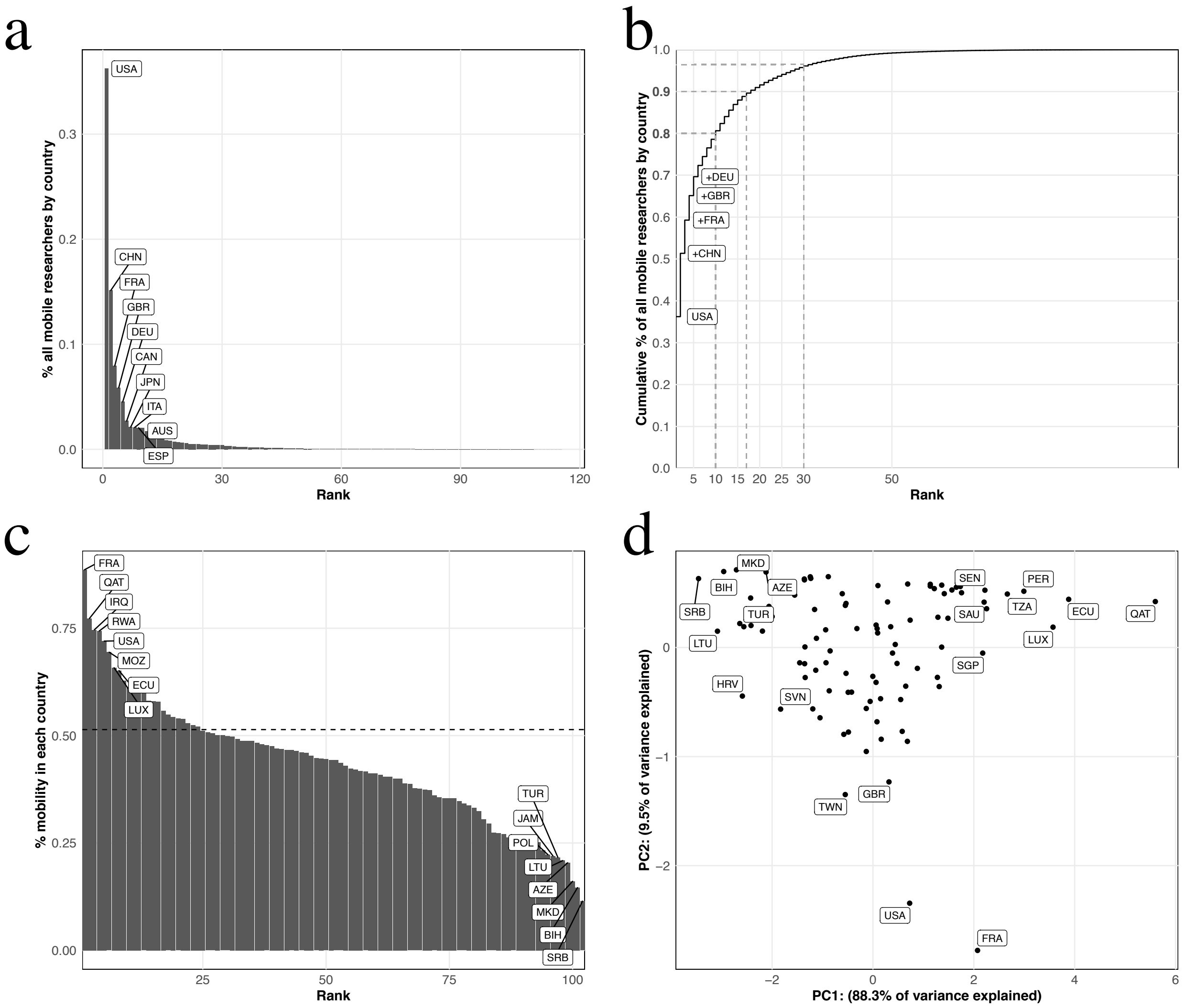

Figure S2: Extent and nature of mobility by country. **a.** The proportion of all mobile researchers contributed by each country. Over 30% of all mobile researchers have been affiliated with organizations in the U.S. during the period of study. **b.** Cumulative distribution of data shown in (**a**). The U.S., China, and France, the U.K., and Germany comprise about 70% of all mobile researchers. **c.** The proportion of each country's researchers who are mobile. The dashed line indicates the proportion of all researchers in the data who are mobile. France, followed by Qatar and the U.S. have the highest proportion of mobile researchers. **d.** First two principal components of four variables: proportion of researchers in each country mobile across organizations, proportion mobile across cities, proportion mobile across regions, and proportion mobile across countries. The countries are roughly sorted in order of the number of mobile researchers and the fraction of international mobile researchers in the first and second principal components, which are indicated by PC1 and PC2, respectively. PC1 explains 88.3% of the total variance, whereas PC2 explains 9.5% of the total variance.

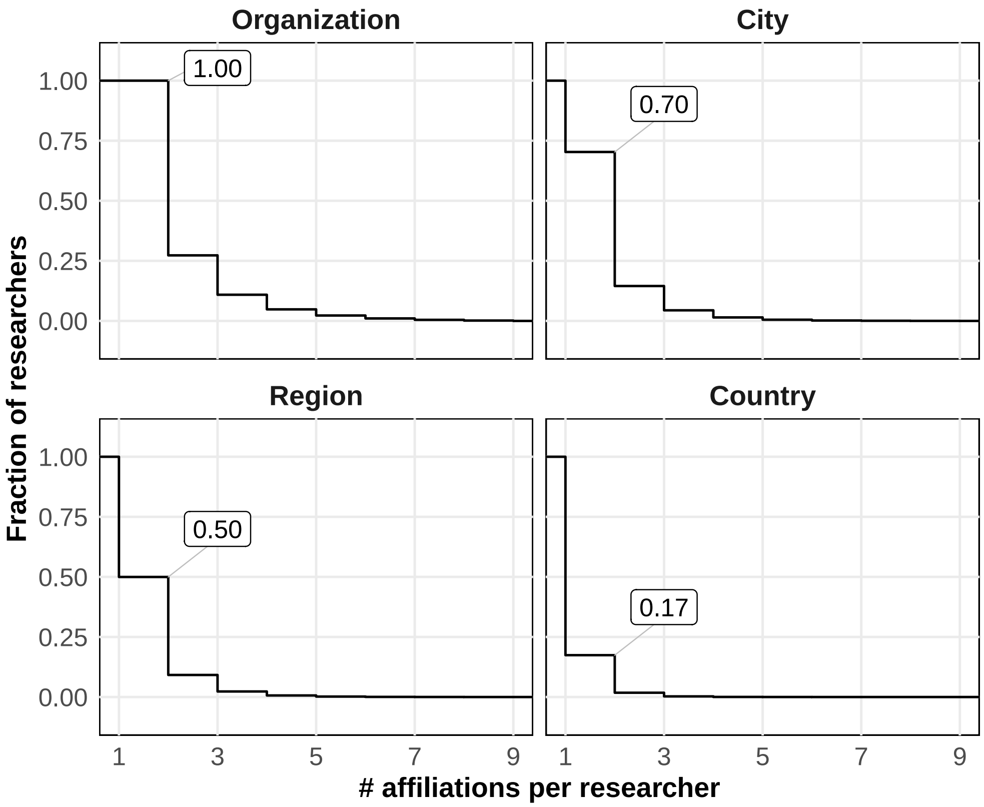

Figure S3: Reverse cumulative-distribution function of mobile researchers by geographic scale. a. Survival probability of mobile researchers with respect to the number of organizations in the their affiliation trajectory. All mobile authors were affiliated with at least two organizations (i.e., survival probability of one) and about 25.0% were affiliated with three or more. **b.** About 70% of mobile authors listed at least two cities represented in their career trajectories. **c.** 50% of mobile authors have two or more regions represented in their career trajectories. **d.** Only 17% of mobile authors had two or more countries represented in their career trajectories.

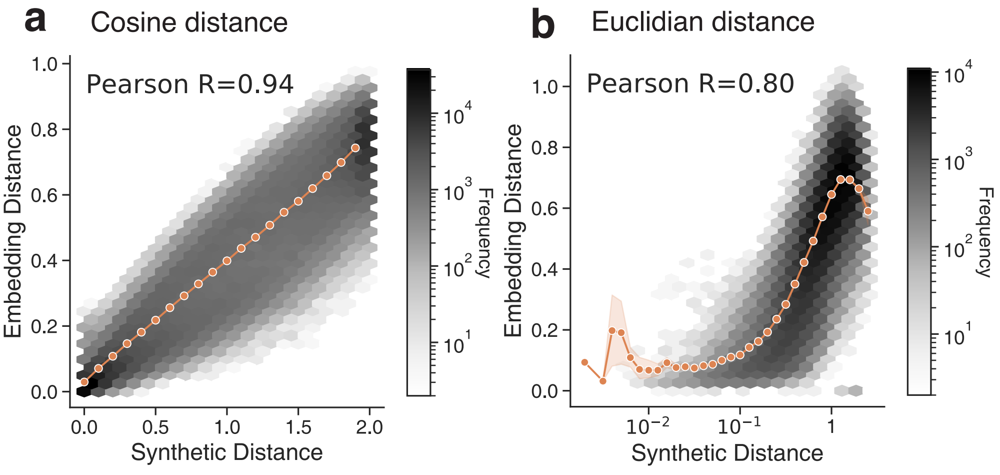

Figure S4: Neural embedding recovers the underlying distance from trajectories. a. The trajectories generated with cosine distance an **b.** The trajectories generated with Euclidean distance. Illustratively, we show the result of the representative iteration of the experiment whose correlation is close to the average. The embedding distance shows a good agreement with underlying distance. Orange dots represent the average embedding distance across binned synthetic distances with standard error.

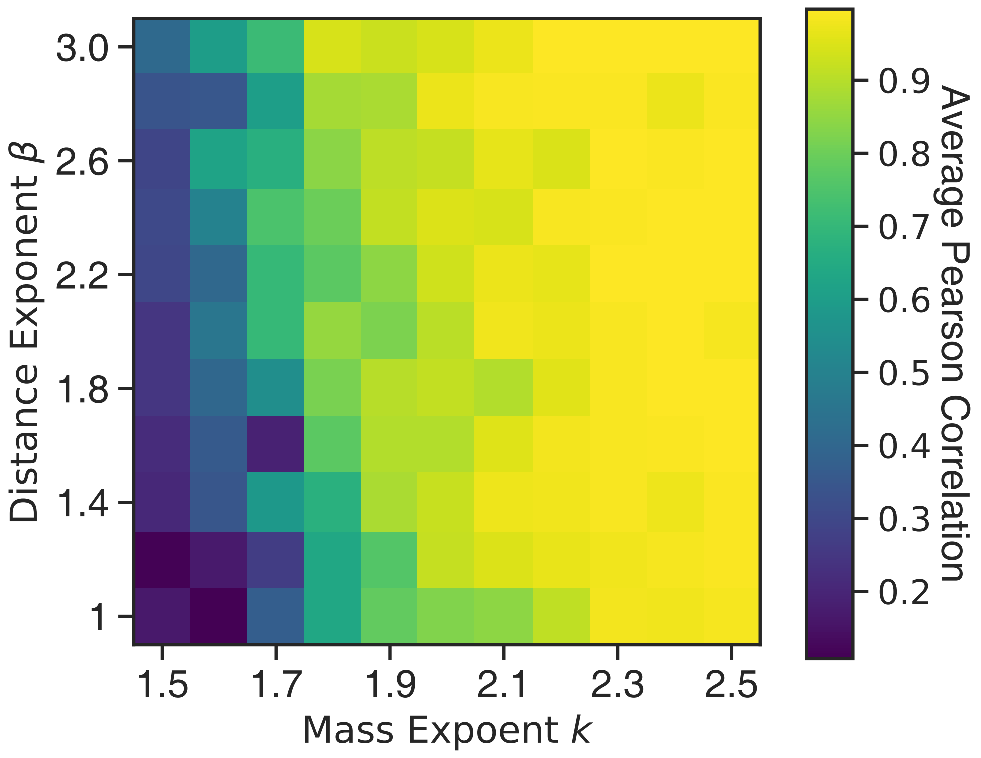

Figure S5: Neural embedding consistently recovers the underlying distance from trajectories across diverse hyperparameter settings. We repeat our synthetic benchmark varying mass exponent k and distance exponent β. The color of each cell represents the average Pearson correlation of the synthetic distance and embedding distances over ten repetitions with given k and β.

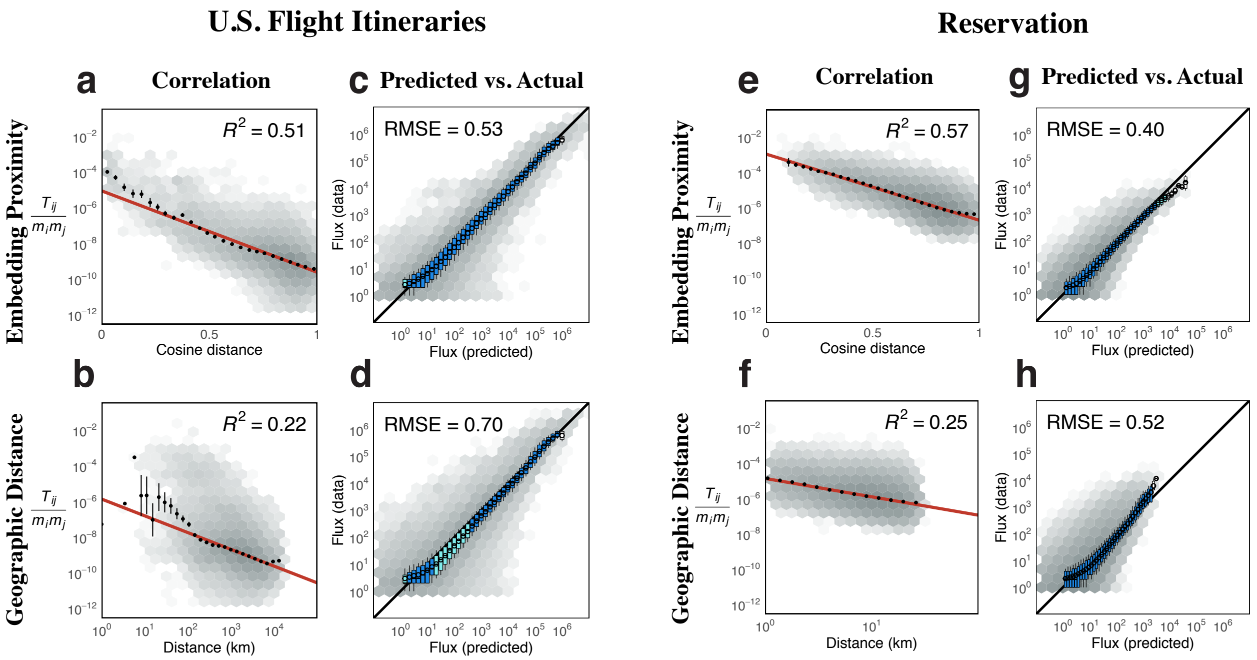

Figure S6: Neural embedding provides functional distance that improves predictive power of the gravity model of migration best in distnct human trajectory datasets a. Embedding distance better explains the expected flux of passengers in U.S. flight itineraries than does geographic distance (**b**). The red line is the line of the best fit. Black dots are mean flux across binned distances. 99% confidence intervals are plotted for the mean flux in each bin. Correlation is calculated on the data in the log-log scale ($p < 0.0001$ across all fits). The lightness of each hex bin indicates the frequency of organization pairs within it. **d.** Predictions of flux between airport pairs made using embedding distance outperform those made using geographic distance (**e**). Box-plots show the distribution of actual flux for binned values of predicted flux. Box color corresponds to the degree to which the distribution overlaps with $y = x$. "RMSE" is the root-mean-squared error between the actual and predicted values. Embedding distance consistently produces powerful functional distance for Korean accommodation reservations (**f-h**)

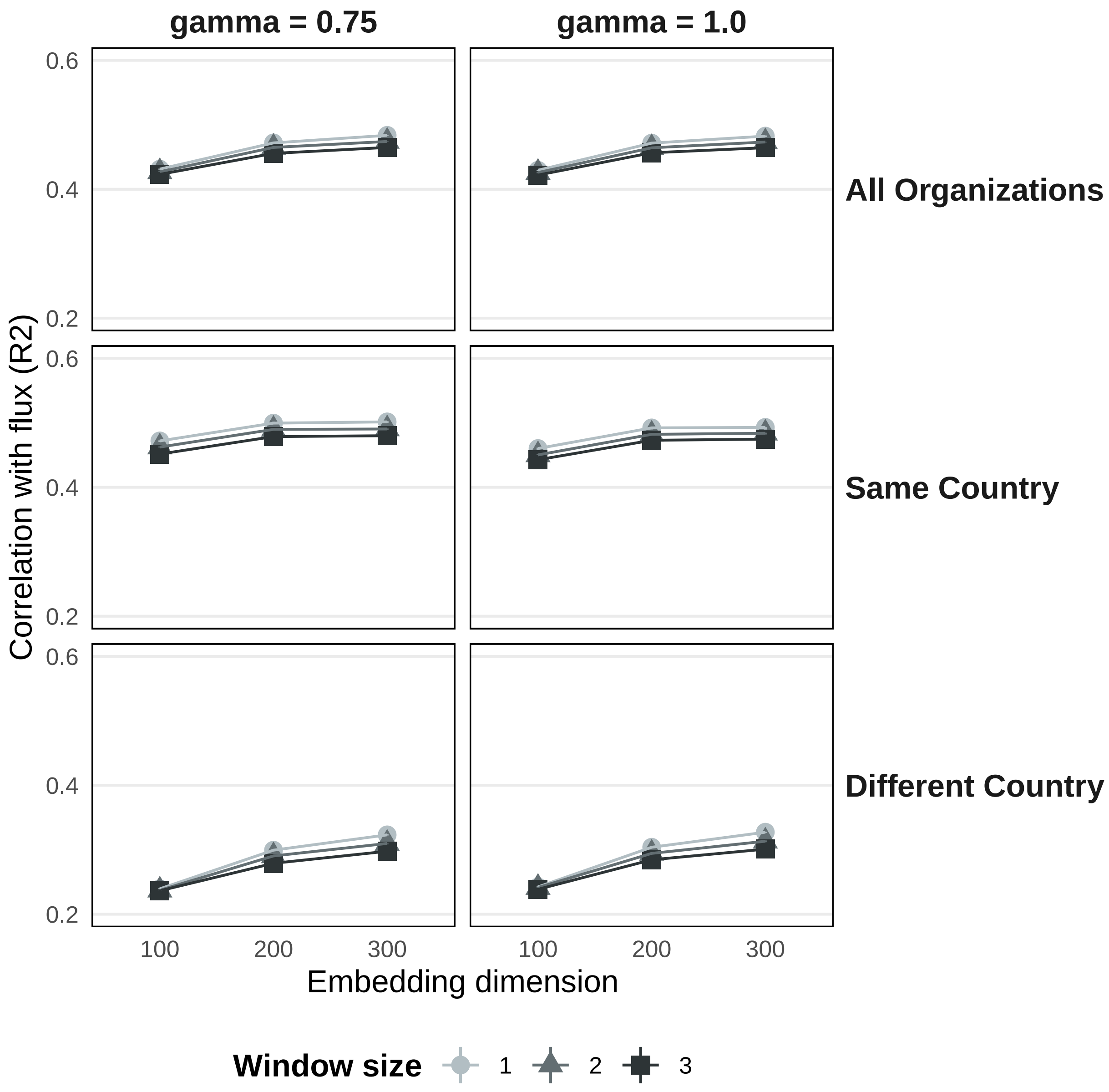

Figure S7: Larger dimensions, smaller window size improves embedding performance. The correlation, or amount of flux explained by the embedding distance with varying skip-gram negative sampling hyperparameters. Window size refers to w, the size of the context window that defines the context in a trajectory. Smaller window sizes result in an embedding that explains more flux. Embedding dimensions refer to the size of the embedding vector. Larger vectors perform better, though with little difference between 200 and 300. Gamma refers to the γ parameter in *word2vec*, which shapes the negative sampling distribution. A value of $\gamma = 0.75$ is the default for *word2vec*. There is virtually no difference in performance based on γ. All variants perform better on same-country organization pairs, and worse on different-country pairs, than on all pairs of of organizations than on all pairs. Embeddings with larger dimensions outperform mid-size embeddings for the different-country case.

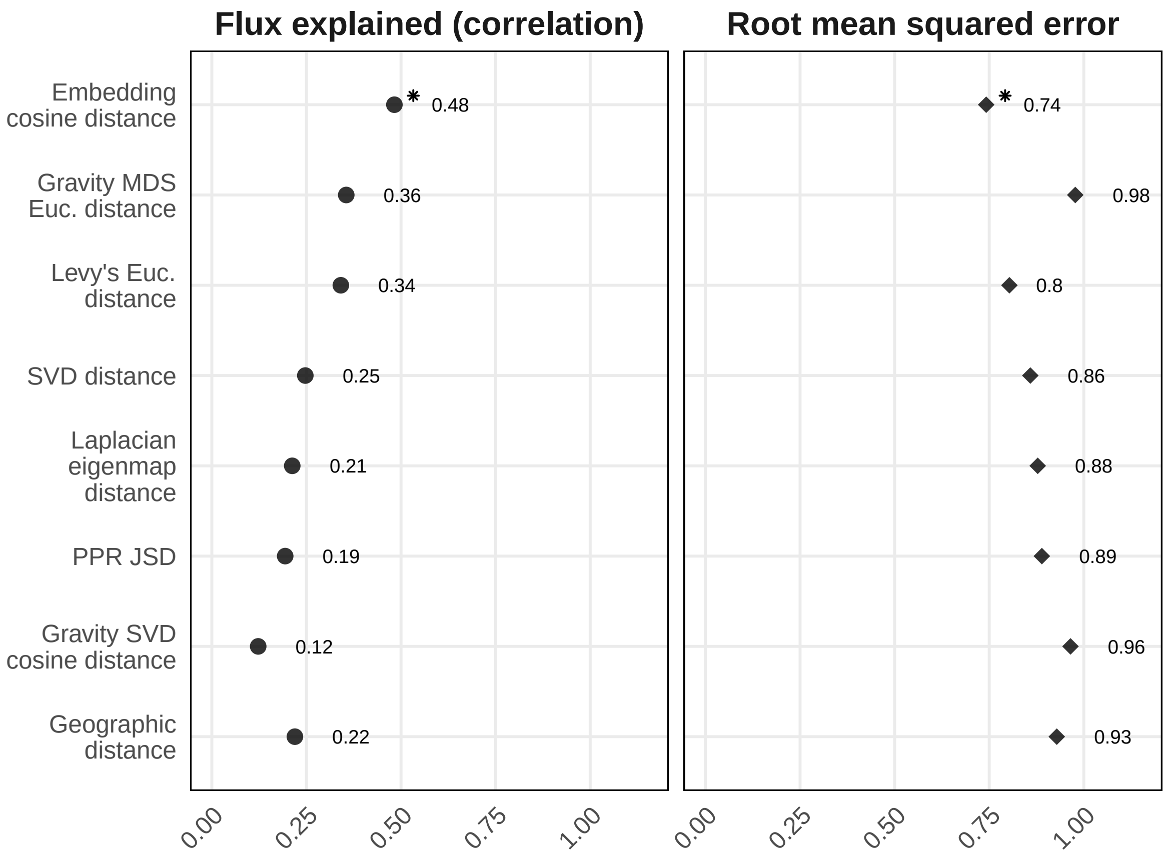

Figure S8: Neural embeddings outperform baselines for scientific mobility. Cosine distance between embedding vectors generated with *word2vec* explains more of the flux, and better predicts flux when used in the gravity model of mobility than geographic distance and other baselines. Shown is the correlation between the flux and embedding distances, measured with R^2 (left), and the prediction error when using the distance as input to the gravity model of mobility. The asterisk denotes the top-performing metric. For prediction error, we show results based on both the exponential and power-law forms of the gravity model. All embedding-based methods use dimensions of 300. Here, embedding distance is obtained from neural embeddings learned with window size of 1 and $\gamma = 1$. In all cases, organization population is defined as the mean annualized number of unique mobile and non-mobile authors, and flux is calculated for all global mobility. Baselines include the top-performing distance metrics calculated between vectors obtained by personalized-page rank (PPR), singular value decomposition (SVD), laplacian eigenmap, direct-factorization following Levy's approach[31], and direct optimization of the gravity model using SVD and multi-dimensional scaling (MDS), as well as the geographic distance between organizations. Embedding distance better explains and predicts flux than any other baseline, though there is some variation by experimental parameters (Table S3, Table S4, and Table S5).

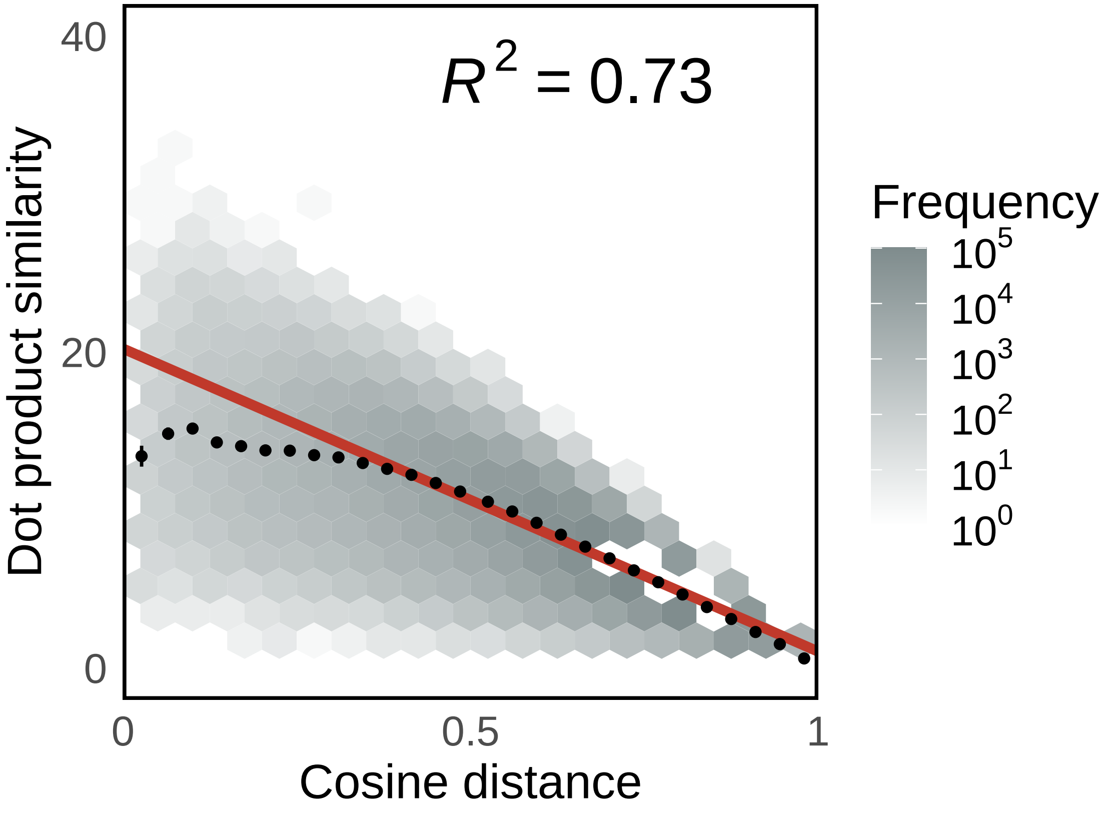

Figure S9: Cosine distance is correlated with dot product similarity. We find a relatively high correlation between the embedding distance—one minus the cosine similarity—and the dot product similarity between organization vectors ($R^2 = 0.73$). Color of each hex bin indicates the frequency of organization pairs. The red line is the line of the best fit. Black dots are mean flux across binned distances. 99% confidence intervals are plotted for the mean flux in each bin based on a normal distribution. Correlation is calculated on the data in the log-log scale ($p < 0.0001$).

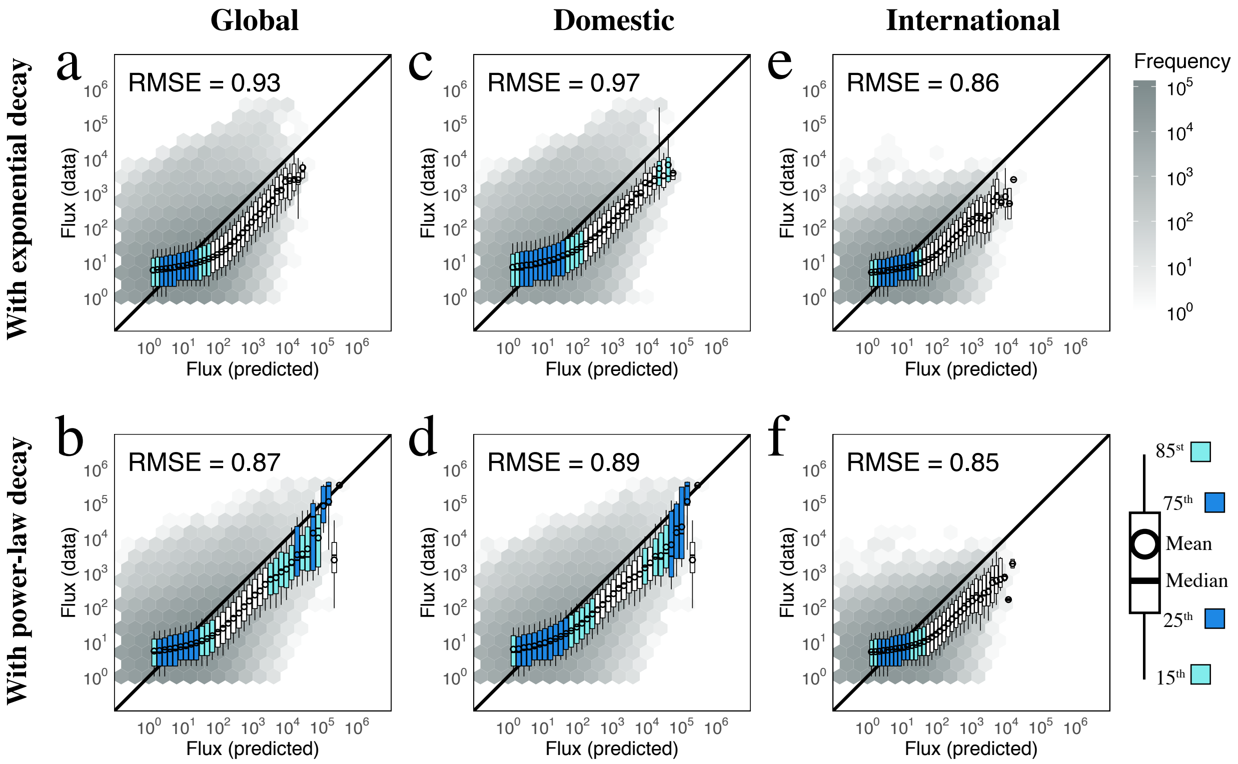

Figure S10: For geographic distance, the power-decay gravity model is better. Flux between organization pairs predicted by the gravity model with different distance decay functions, i.e., exponential decay function (**a**) and power-law decay function (**b**) using geographic distance. Boxplots show distribution of actual flux for binned values of predicted flux. Box color corresponds to the degree to which the distribution overlaps with $x = y$; a perfect prediction yields all points on the black line. "RMSE" is the root-mean-squared error between the actual and predicted values. Shown for all pairs of organization (**a-b**), domestic (**c-d**), and international only (**e-f**) mobility. The gravity model with the power-decay function outperforms that with an exponential decay function.

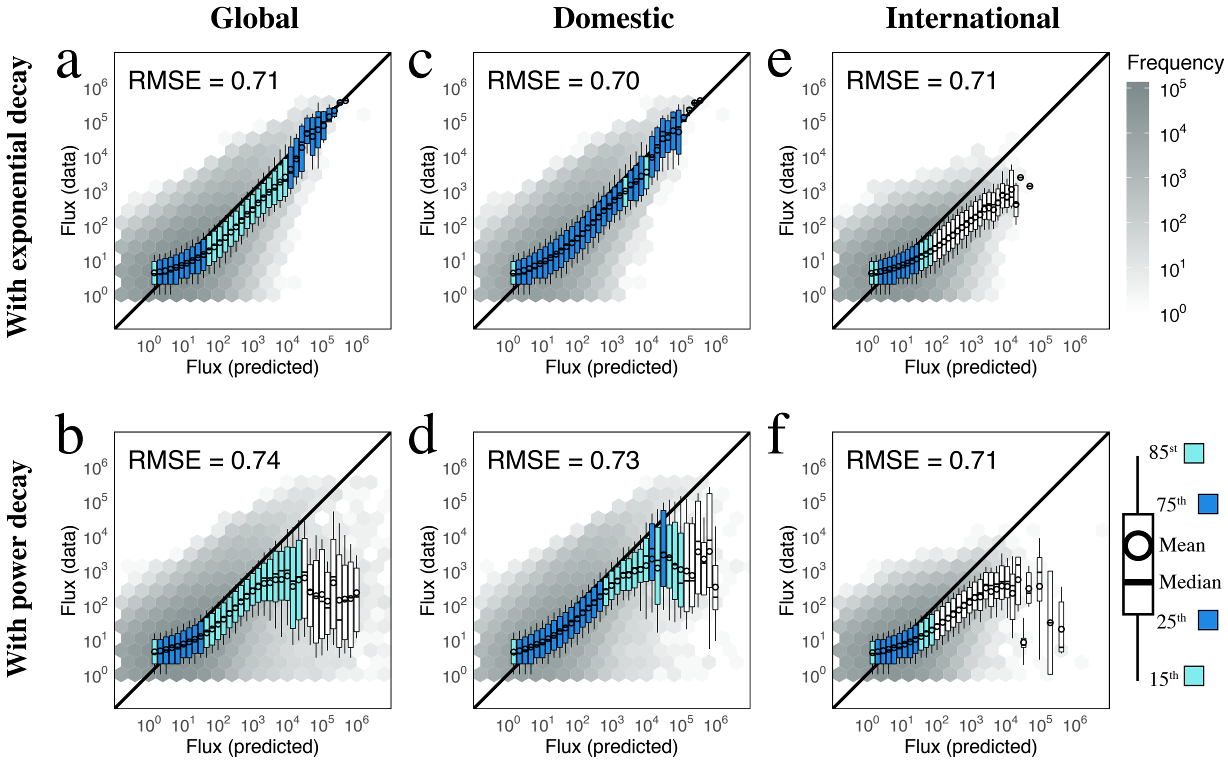

Figure S11: For embedding distance, the exponential-decay gravity model is slightly better. Flux between organization pairs predicted by the gravity model with different distance decay functions, i.e., exponential decay function (**a**) and power-law decay function (**b**) using embedding distance. Boxplots show the distribution of actual flux for binned values of predicted flux. Box color corresponds to the degree to which the distribution overlaps with $x = y$; a perfect prediction yields all points on the black line. "RMSE" is the root-mean-squared error between the actual and predicted values. Shown for all pairs of organization (**a-b**), domestic (**c-d**), and international only (**e-f**) mobility. The gravity model with the exponential decay function slightly outperforms that with a power-decay function except in the case of international-only mobility, for which power-decay performs slightly better.

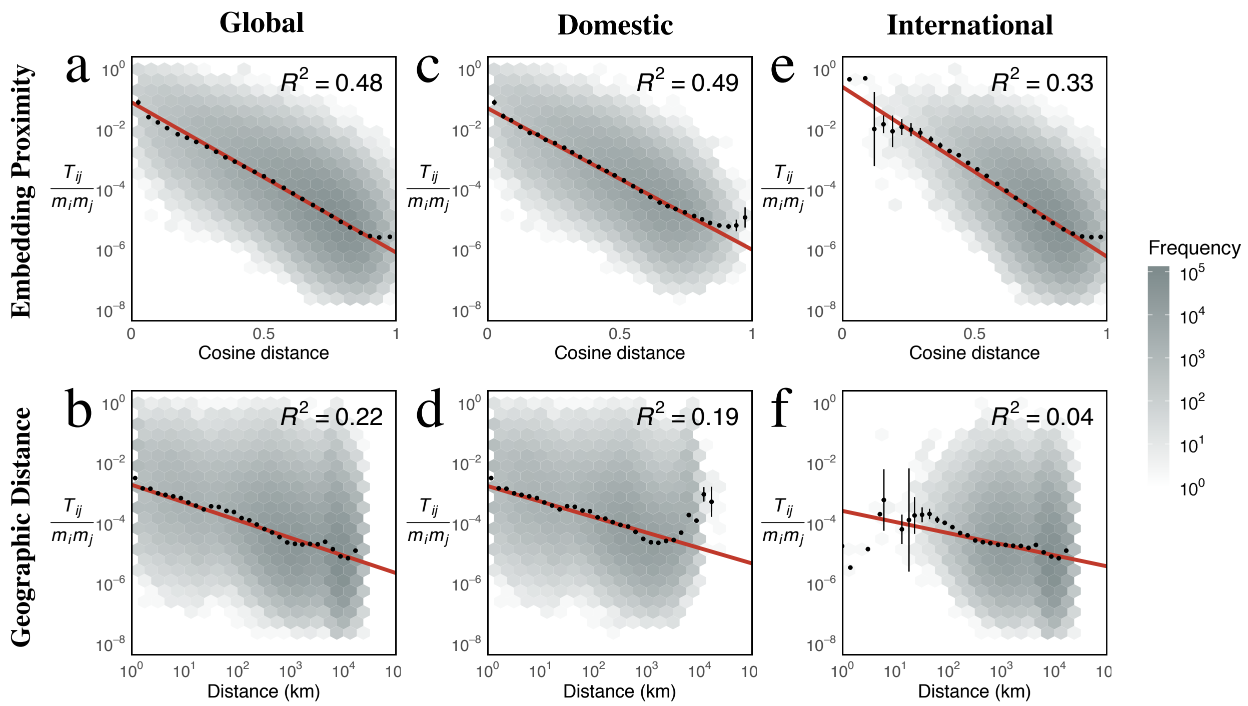

Figure S12: Embedding distance explains more variance for global, within, and across country flux than geographic distance. a. Embedding distance explains more flux than geographic distance (**b**). The red line is the line of the best fit. Black dots are mean flux across binned distances. 99% confidence intervals are plotted for the mean flux in each bin based on a normal distribution. Correlation is calculated on the data in the log-log scale (p < 0.0001 across all fits). Color of each hex bin indicates frequency of organization pairs. Results here are identical to those shown in manuscript. **c-d.** embedding distance explains more variance when considering only within-country organization pairs. **e-f.** embedding distance is more robust than geographic distance when considering only across-country organization pairs.

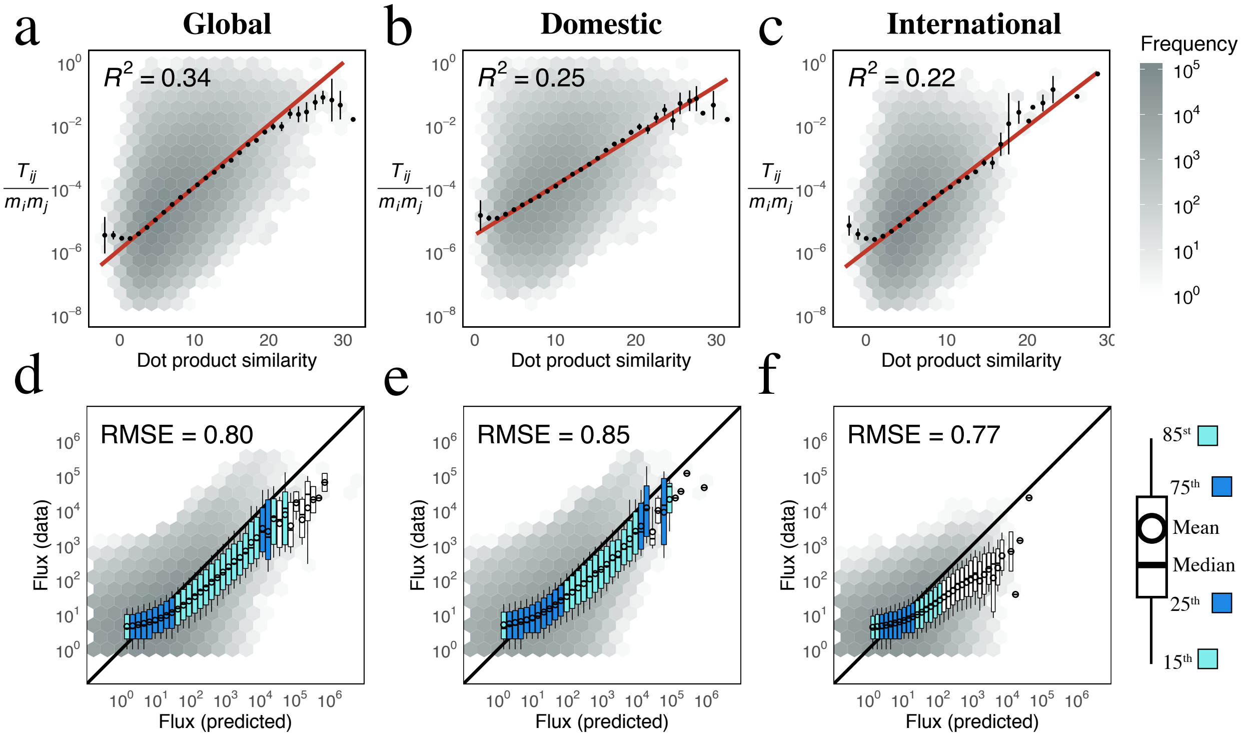

Figure S13: Examine gravity model with dot product on the embedding space. Performance of dot product similarities in explaining and predicting mobility. Similarity scores are calculated as the pairwise dot product between organizational vectors. Dot product similarity performs better than geographic distance, though worse than cosine similarity in explaining global mobility (**a**), or domestic (**b**) or international (**c**) country mobility. The red line is the line of the best fit. Black dots are mean flux across binned distances. 99% confidence intervals are plotted for the mean flux in each bin based on a normal distribution. Correlation is calculated on the data in the log-log scale ($p < 0.0001$ across all fits). Color indicates frequency of organization pairs within each hex bin. Similarly, PPR distance performs comparably to geographic distance in predicting global (**d**), domestic (*e*) and international (**f**) scientific mobility. Boxplots show distribution of actual flux for binned values of predicted flux. Box color corresponds to the degree to which the distribution overlaps $x = y$; a perfect prediction yields all points on the black line. "RMSE" is the root-mean-squared error between the actual and predicted values.

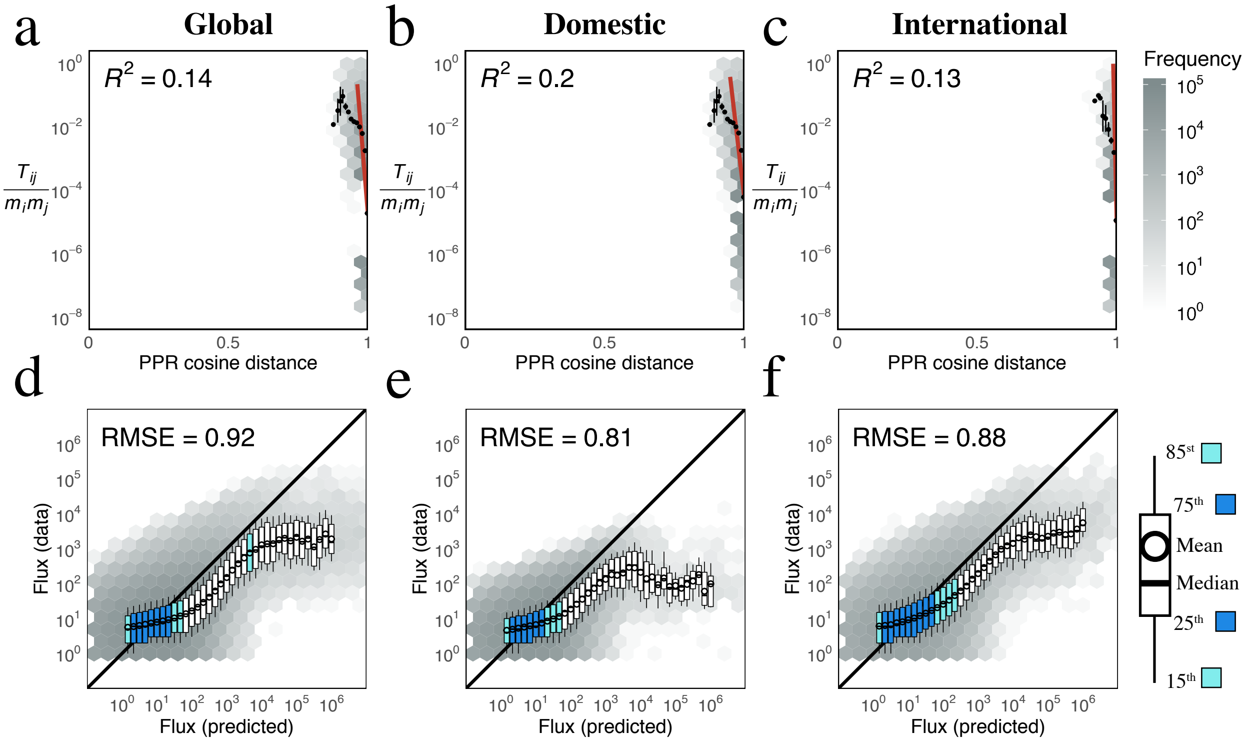

Figure S14: Personalized page rank with cosine distance. Performance of personalized page rank scores in explaining and predicting mobility. Personalized page rank is calculated for the underlying mobility network, and distance measured as the cosine distance between PPR probability distribution vectors. PPR cosine distance performs roughly similar to geographic distance in explaining global(**a**), domestic (**b**), or international (**c**) country mobility. The red line is the line of the best fit. Black dots are mean flux across binned distances. 99% confidence intervals are plotted for the mean flux in each bin based on a normal distribution. Correlation is calculated on the data in the log-log scale ($p < 0.0001$ across all fits). Color of hex bind indicates frequency of organization pairs. Similarly, PPR distance performs comparably to geographic distance in predicting global (**d**), domestic (e) and international (**f**) scientific mobility. Boxplots show distribution of actual flux for binned values of predicted flux. Box color corresponds to the degree to which the distribution overlaps $x = y$; a perfect prediction yields all points on the black line. "RMSE" is the root-mean-squared error between the actual and predicted values.

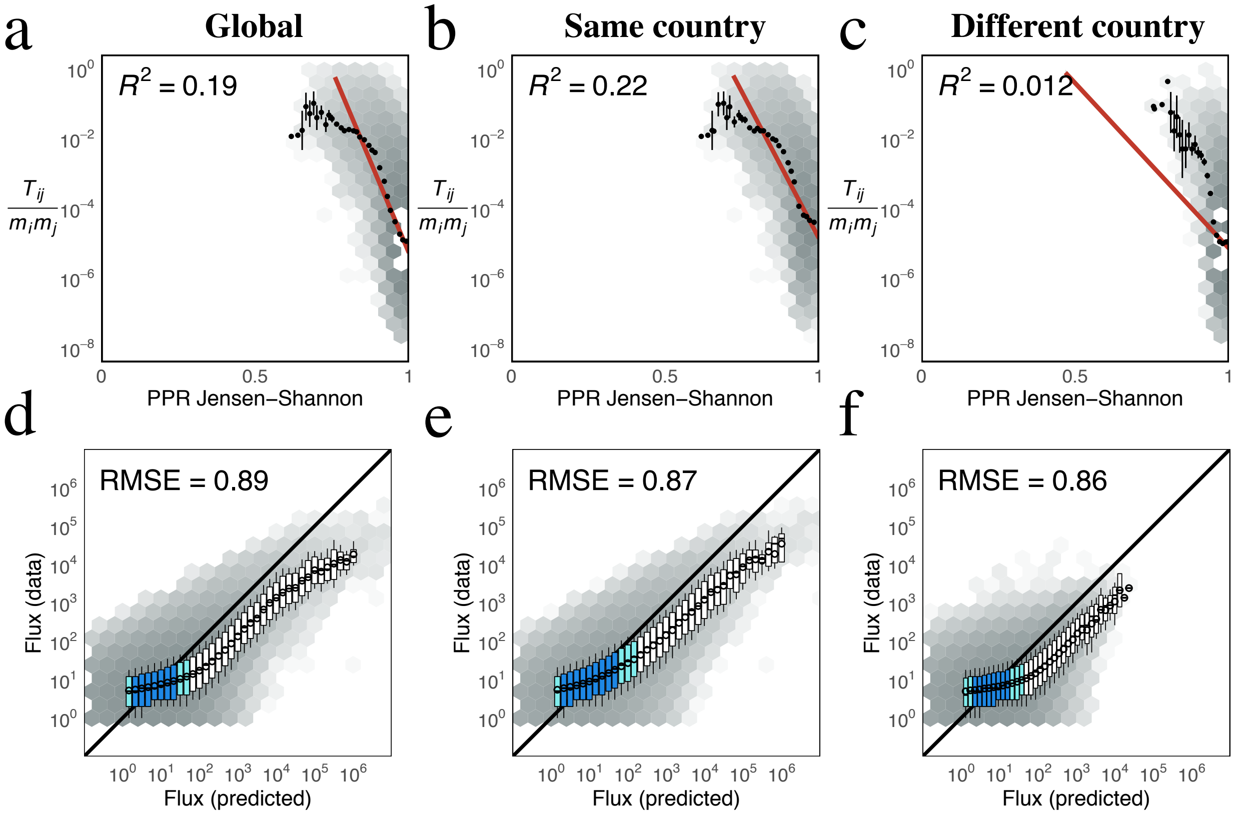

Figure S15: Personalized page rank with Jensen-Shannon Divergence. Performance of personalized page rank scores in explaining and predicting mobility. Personalized page rank is calculated for the underlying mobility network, and distance measured as the Jensen-Shannon Divergence (JSD) between PPR probability distribution vectors. PPR JSD performs roughly similar to geographic distance in explaining global mobility (**a**), or domestic (**b**) or international (**c**) country mobility. Overall, PPR JSD explains more variance in mobility than using cosine distance (Fig. S14), except for international mobility, for which cosine similarity out-performs JSD. The red line is the line of the best fit. Black dots are mean flux across binned distances. 99% confidence intervals are plotted for the mean flux in each bin based on a normal distribution. Correlation is calculated on the data in the log-log scale ($p < 0.0001$ across all fits). Color of hex bind indicates frequency of organization pairs. Similarly, PPR JSD performs comparably to geographic distance in predicting global (**d**), domestic (e) and international (**f**) scientific mobility. Boxplots show distribution of actual flux for binned values of predicted flux. Box color corresponds to the degree to which the distribution overlaps $x = y$; a perfect prediction yields all points on the black line. "RMSE" is the root-mean-squared error between the actual and predicted values.

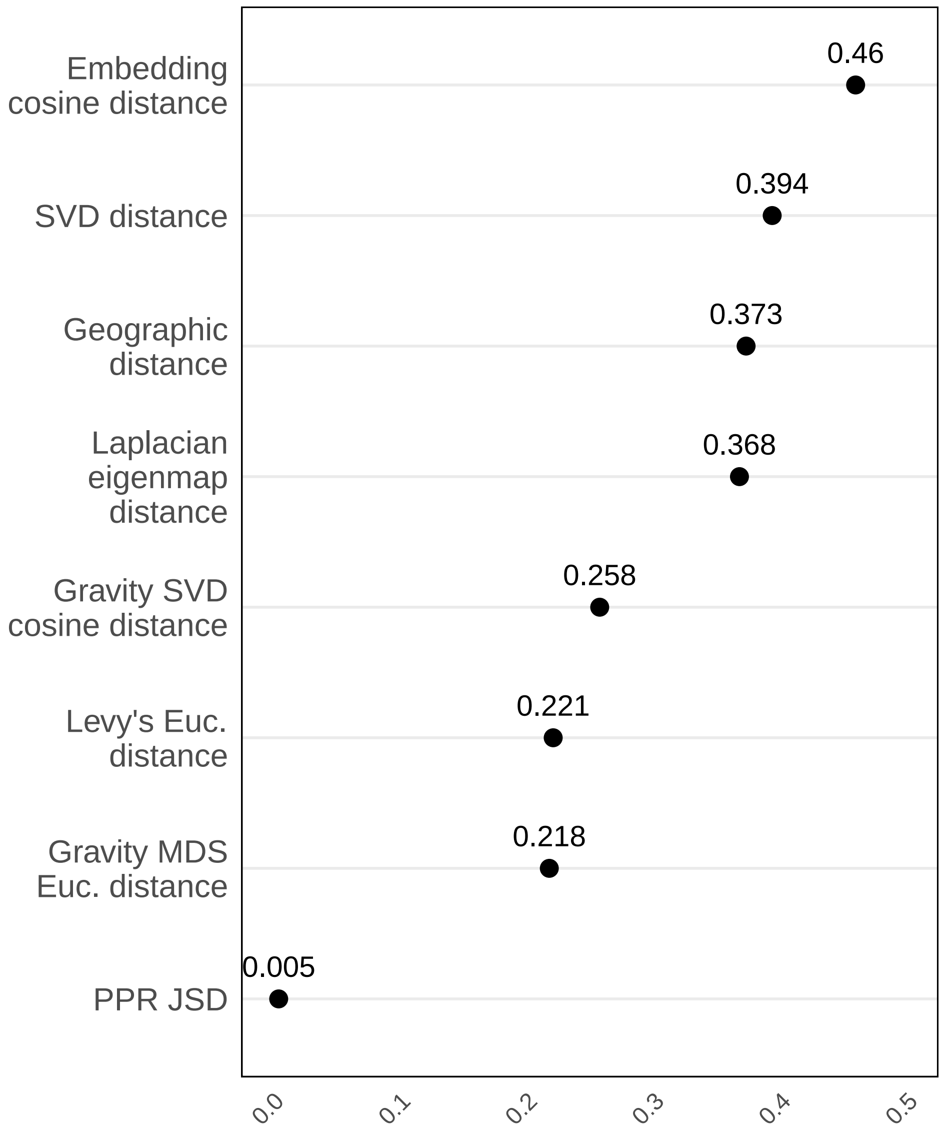

Figure S16: Embedding distance outperforms alternatives in common part of commuters (CPC). The CPC measure is calculated for both the power and exponential form of the gravity model, with the highest value reported for each distance type.

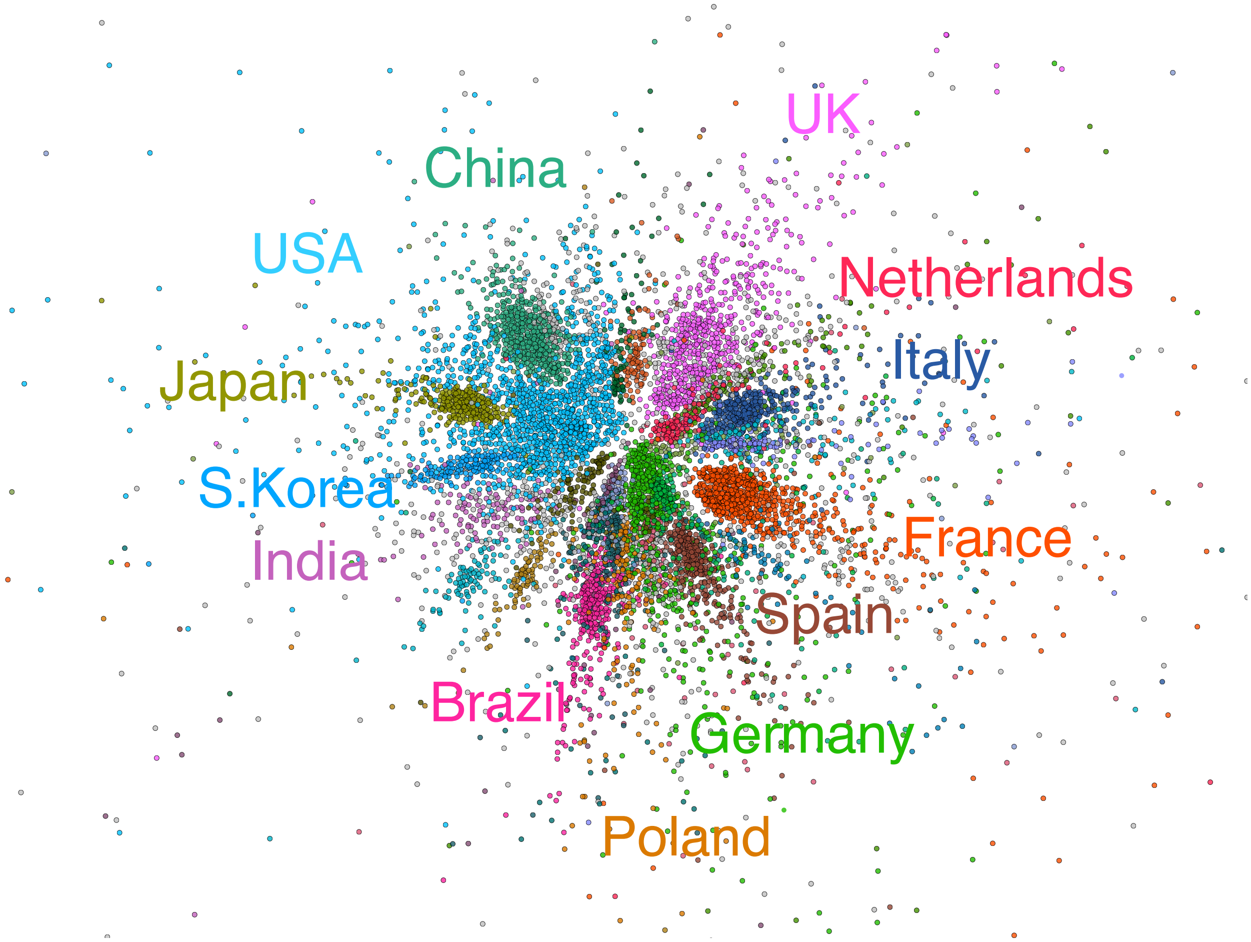

Figure S17: Visualization of global mobility network. The network demonstrates country-level structure, but not at the detail or the extent of the global UMAP projection (Fig. 2a). Each node corresponds to an organization, whereas weighted edges (excluded in this figure for visual clarity) correspond to the flow of mobile researchers between the two organization. Nodes are colored by the country of the organization. Nodes are positioned using the Force Atlas layout algorithm.

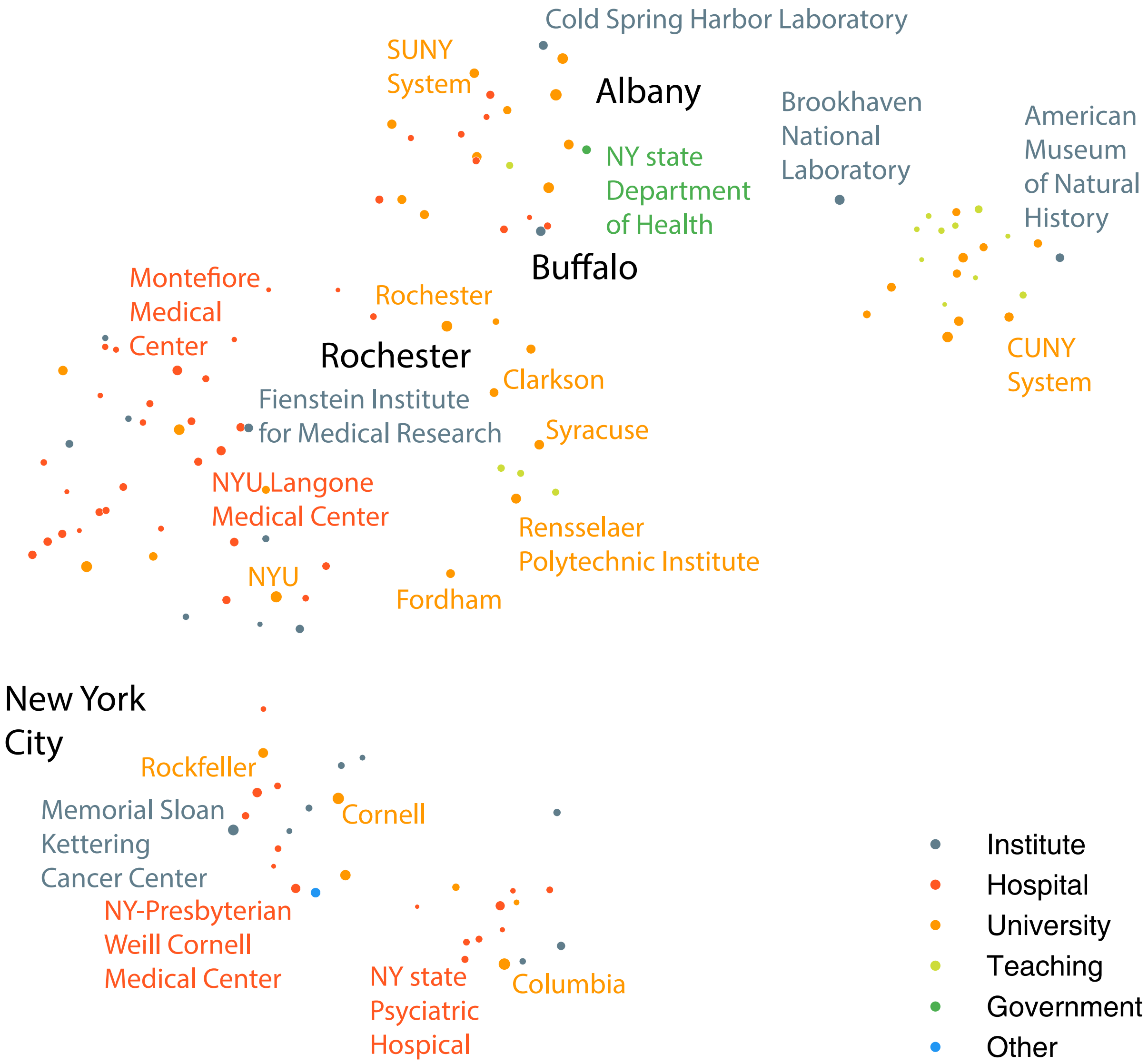

Figure S18: UMAP Projection of organizations in New York. Each point corresponds to an organization and its size indicates the average annual number of mobile and non-mobile authors affiliated with that organization from 2008 to 2019. Color indicates the sector.

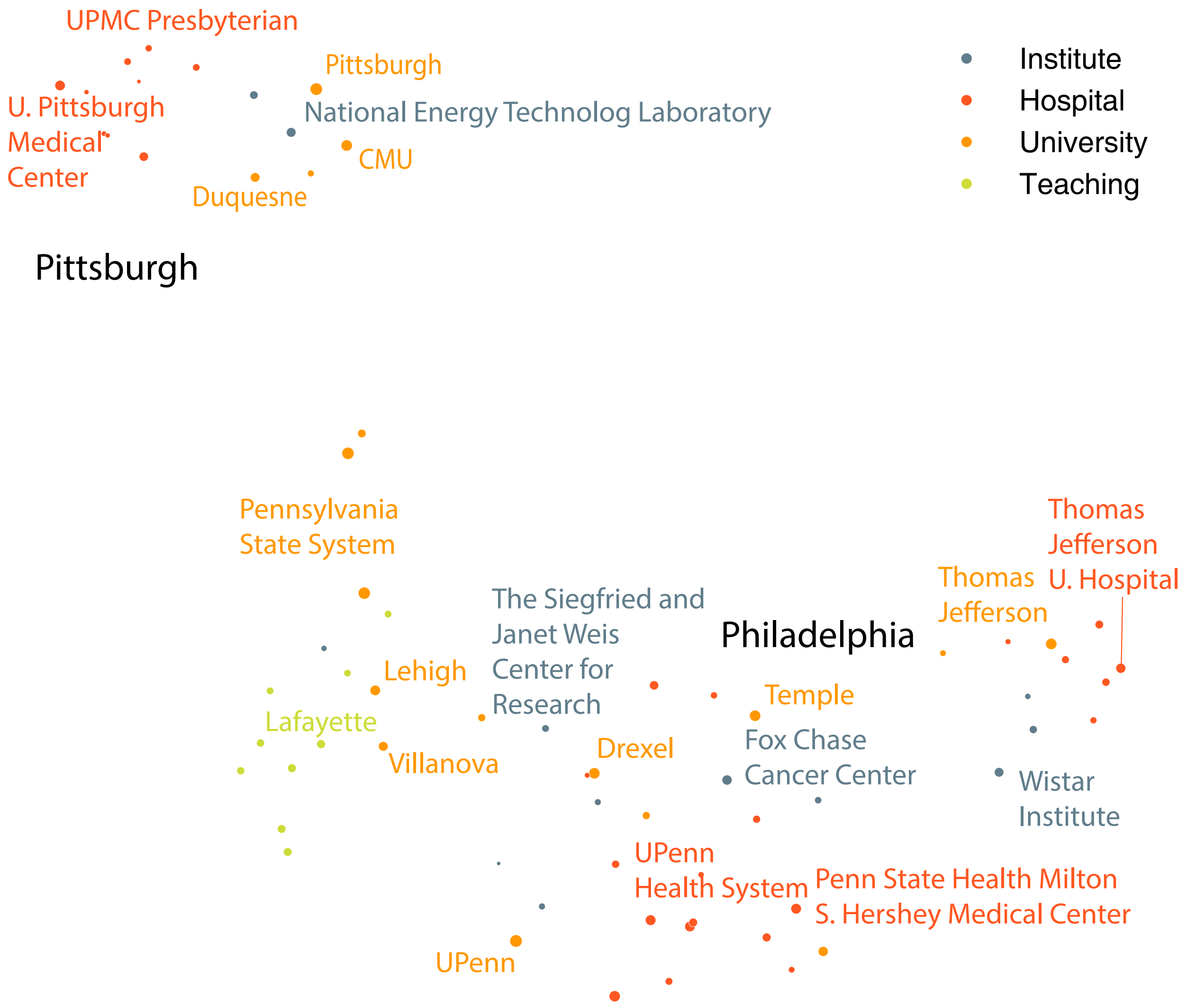

Figure S19: UMAP Projection of organizations in Pennsylvania. UMAP projection of the embedding space of organizations in Pennsylvania reveals clustering based on geography, sector, and academic prestige. Each point corresponds to an organization and its size indicates the average annual number of mobile and non-mobile authors affiliated with that organization from 2008 to 2019. Color indicates the sector.

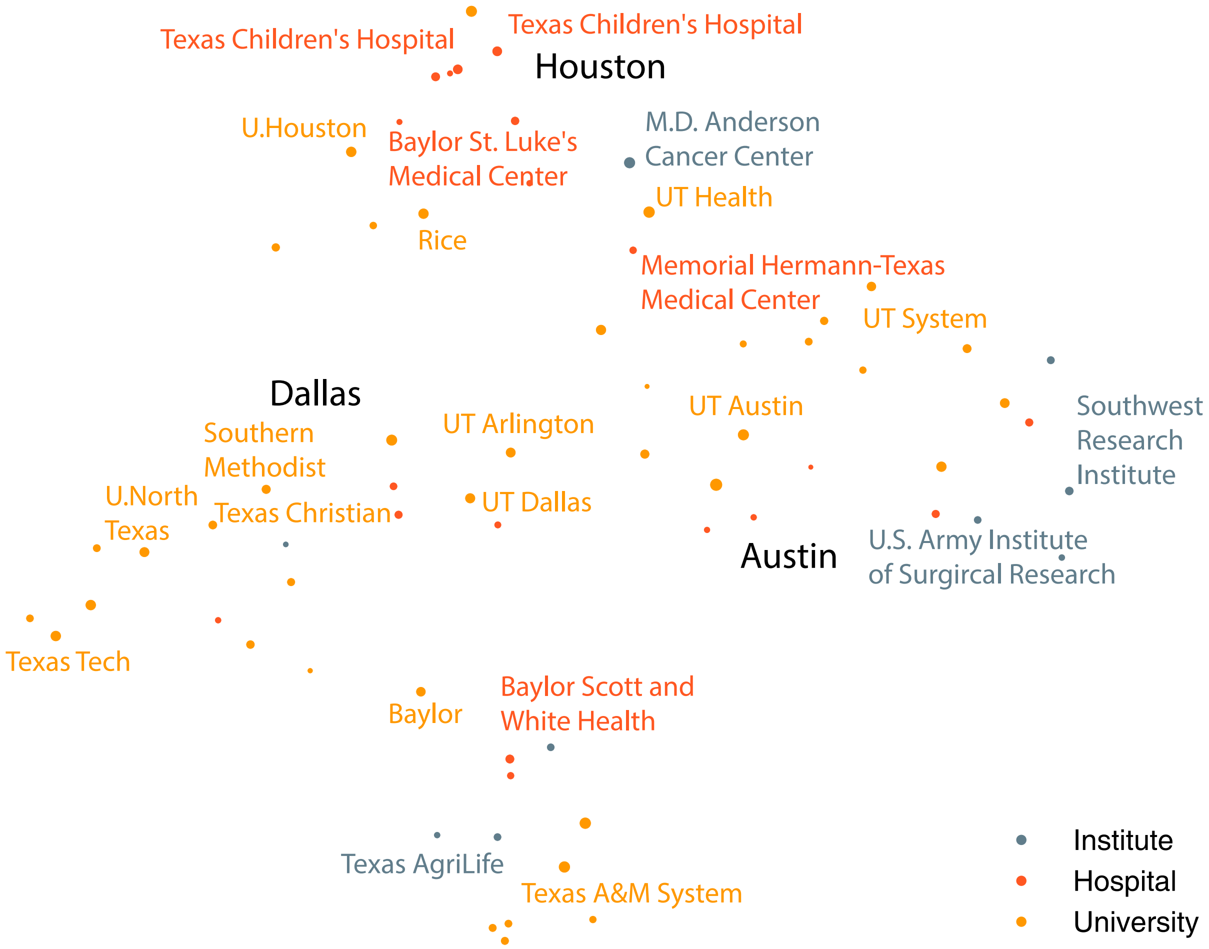

Figure S20: UMAP Projection of organizations in Texas. Each point corresponds to an organization and its size indicates the average annual number of mobile and non-mobile authors affiliated with that organization from 2008 to 2019. Color indicates the sector.

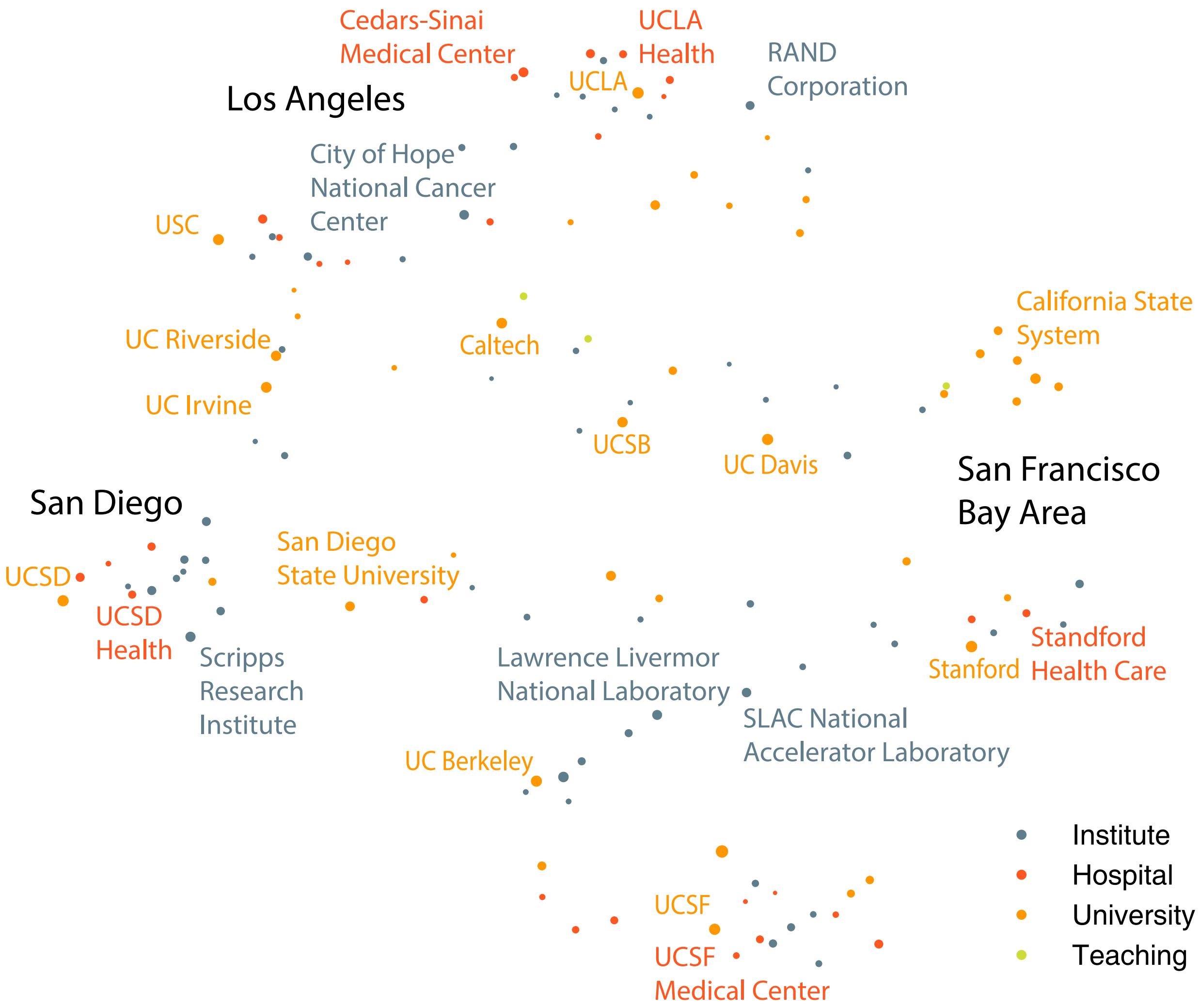

Figure S21: UMAP Projection of organizations in California. Each point corresponds to an organization and its size indicates the average annual number of mobile and non-mobile authors affiliated with that organization from 2008 to 2019. Color indicates the sector.

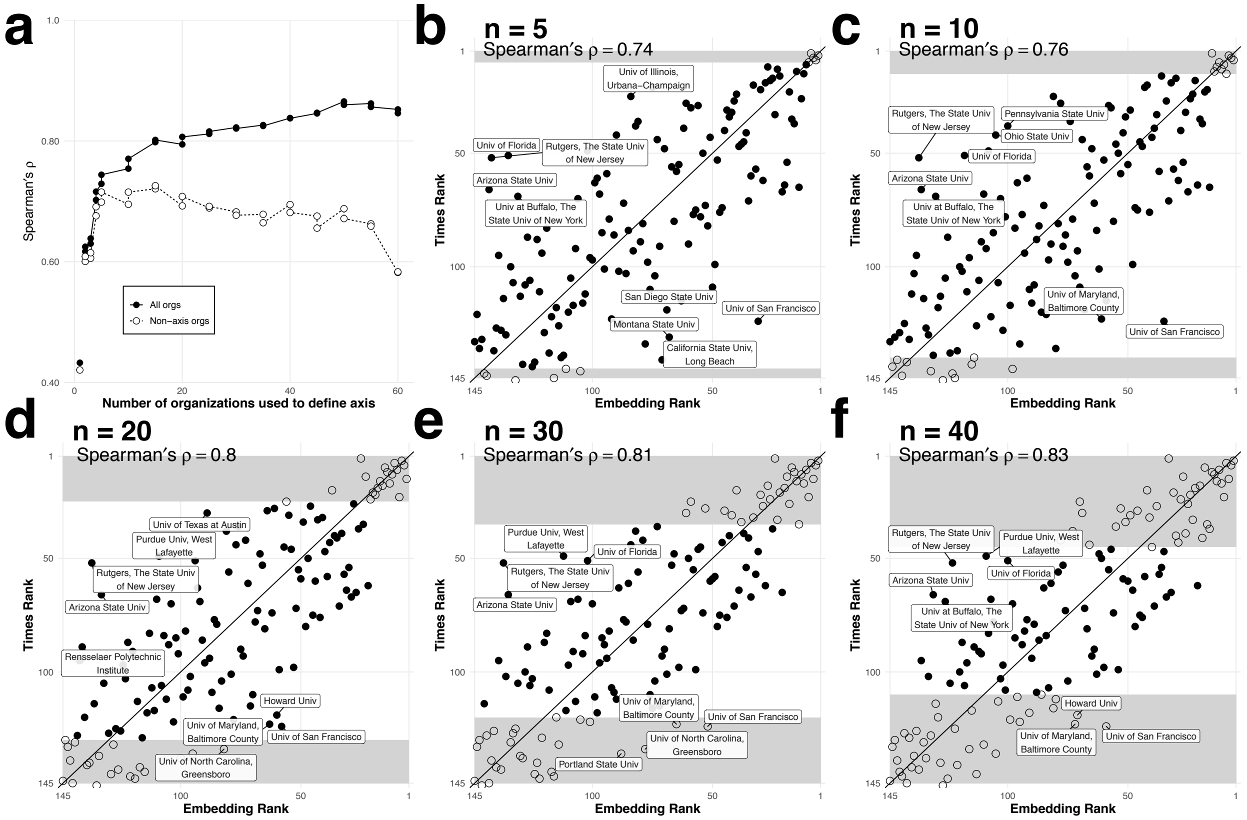

Figure S22: SemRank hierarchy is robust. a. Spearman's ρ (n = 143) between Times prestige rank and embedding rank derived using SemAxis, with poles defined using the top and bottom (geographically matched) ranked universities. Black points show spearman correlation using all organizations; white points show correlation using only universalizes not aggregated in the poles. Including more universities improves performance, but quickly saturates after around five universities. **b - f.** Comparison between the Times and SemAxis ranks of universities, by the number of universities used to define the poles (n). White points are those top and bottom 20 universities aggregated to define the ends of the axis. The grey box corresponds to the top 20 and bottom 20 ranks. Spearman's ρ details the estimate from Spearman correlation between the two rankings using all universities, including those used to define the ends of each axis. All correlations are significant with p < 0.0001.

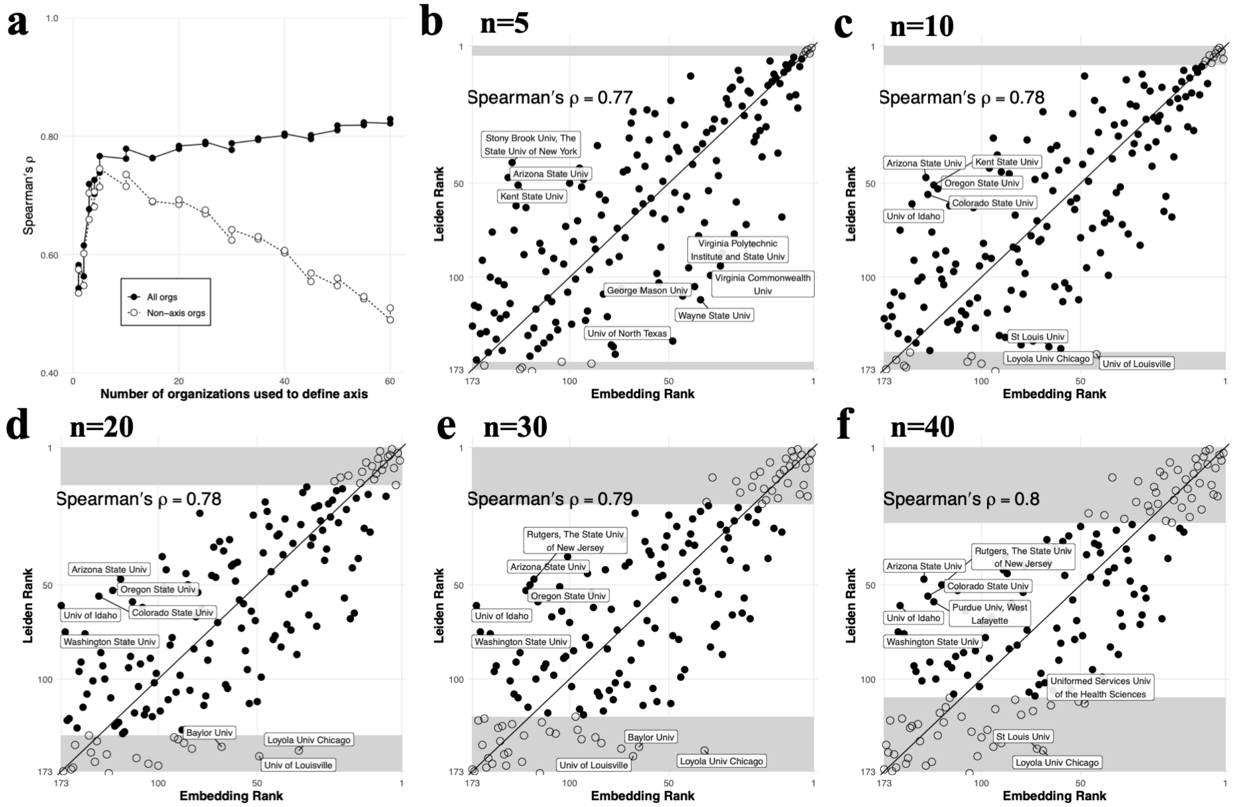

Figure S23: SemRank hierarchy holds when compared to Leiden Ranking. a. Spearman's ρ (n = 143) between Leiden ranking of U.S. universities derived using SemAxis, with poles defined using the top and bottom (geographically matched) ranked universities. Black points show Spearman correlation using all organizations; white points show correlation using only universities not aggregated in the poles. Including more universities improves performance, but quickly saturates after around five universities. **b - f.** Comparison between the Leiden and SemAxis ranks of universities, by the number of universities used to define the poles (n). White points are the top and bottom 20 universities aggregated to define the ends of the axis. The grey box corresponds to the top 20 and bottom 20 ranks. Spearman's ρ details the estimate from Spearman's rank correlation between the two rankings using all universities, including those used to define the ends of each axis. All correlations are significant with $p < 0.0001$.

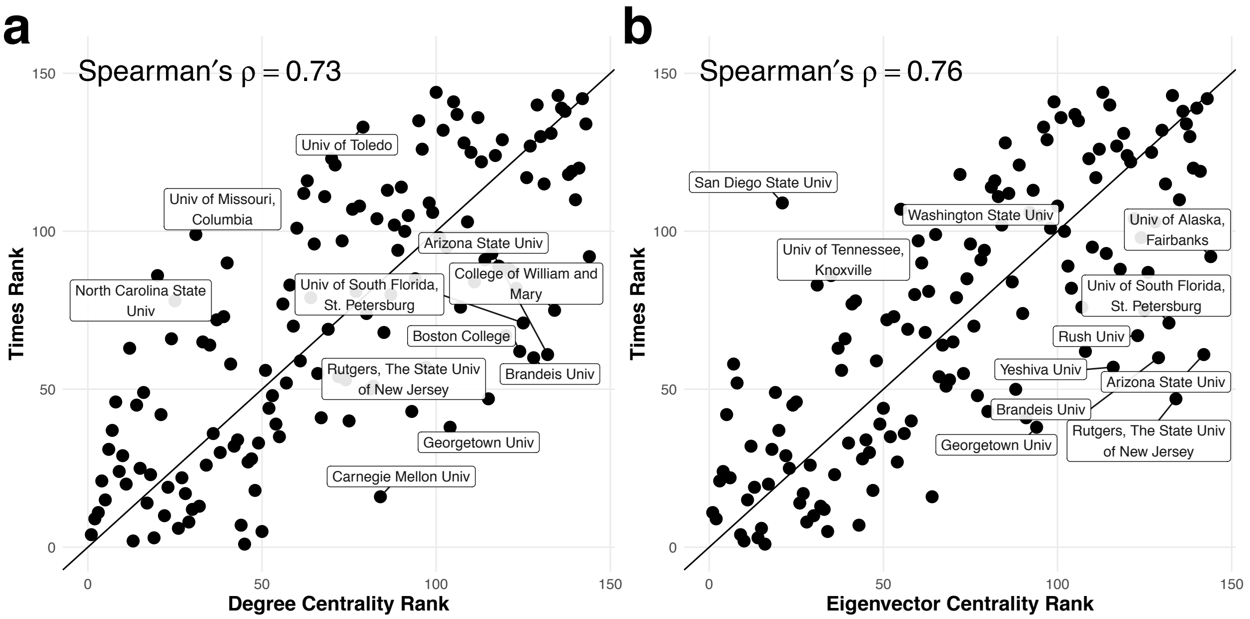

Figure S24: Network centrality is strongly correlated with Times ranking. Comparison between the ranking of organizations by their network-centrality rank and their rank in the 2018 Times Higher Education ranking of U.S. Universities . The Times rank is correlated with degree centrality rank (**a**) with Spearman's $\rho = 0.73$, and is correlated with the eigenvector centrality rank (**b**) with Spearman's $\rho = 0.76$. All correlations are significant with $p < 0.0001$.

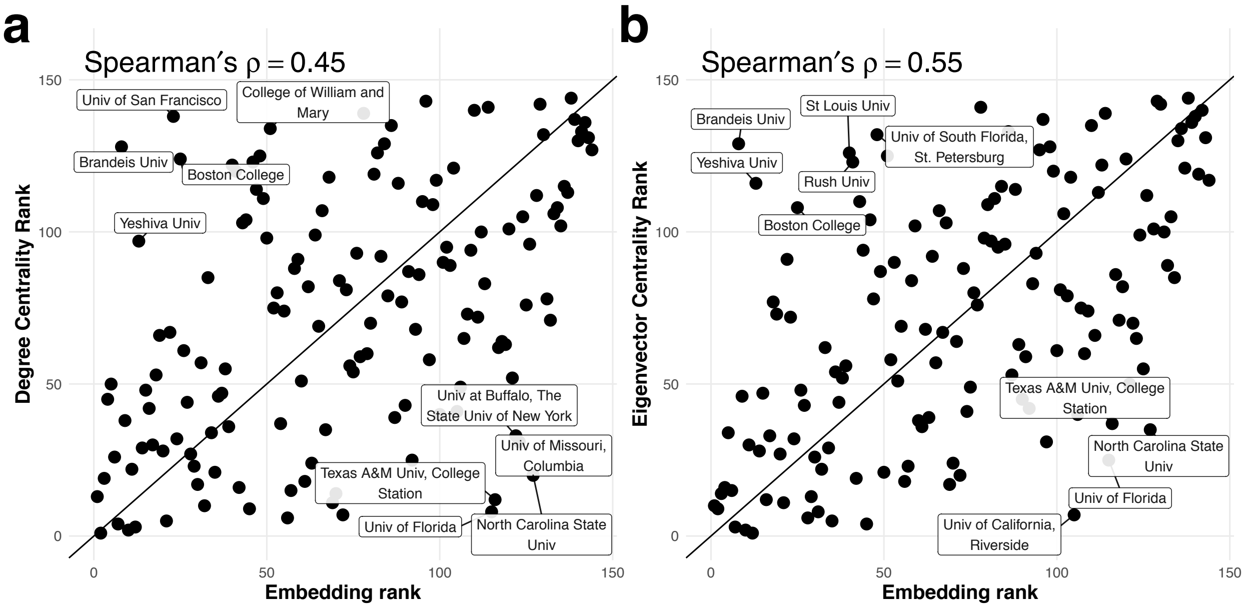

Figure S25: Network centrality less correlated with Embedding rank. Comparison between the ranking of organizations by their network-centrality rank and the embedding rank derived with SemAxis with poles defined using the top five to geographically-matched bottom five universities ranked by the 2018 Times Higher Education ranking of U.S. Universities . Embedding rank is correlated with degree centrality rank (**a**) with Spearman's ρ = 0.45, and is correlated with the eigenvector centrality rank (**b**) with Spearman's ρ = 0.55. All correlations are significant with $p < 0.0001$.

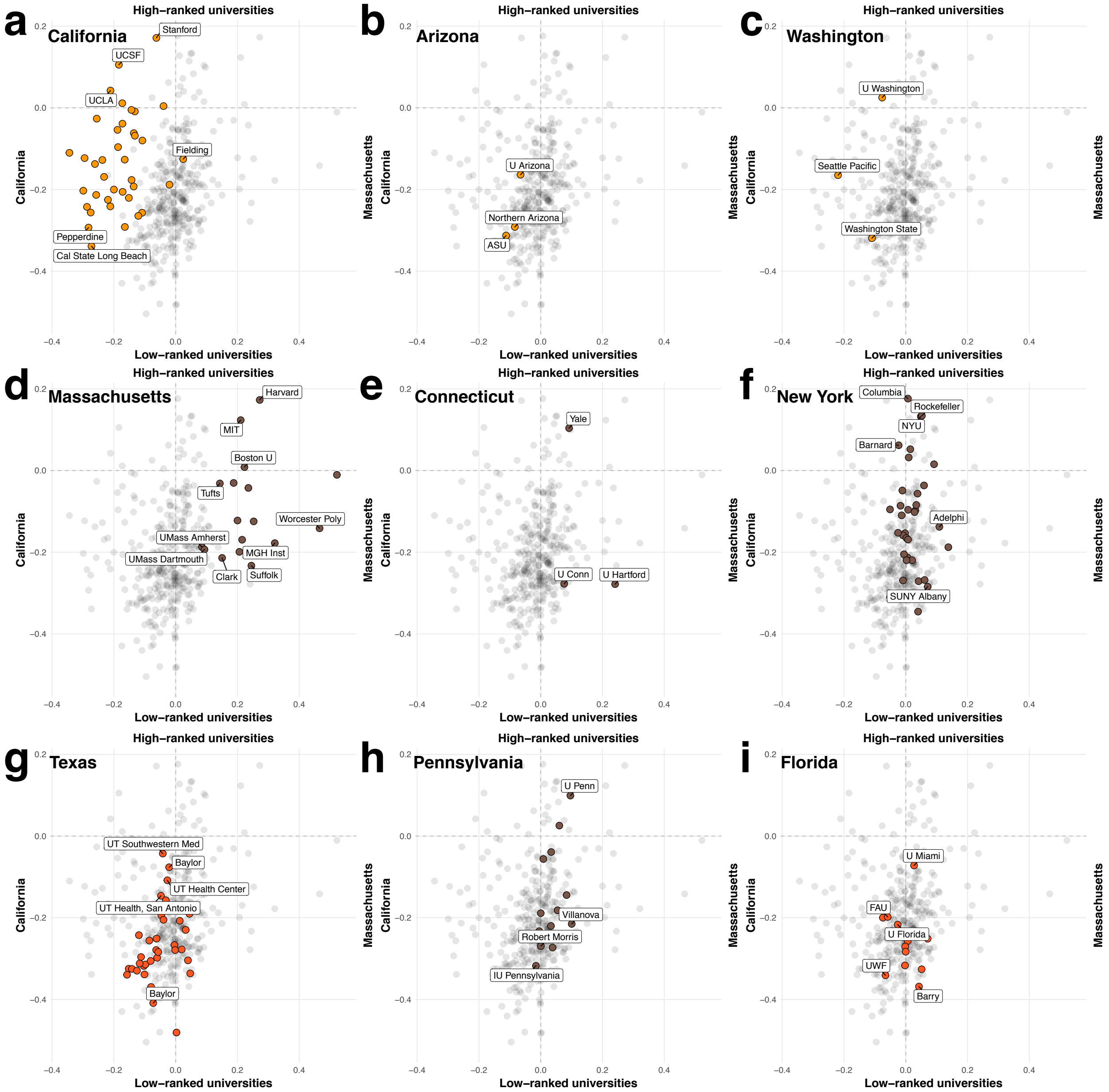

Figure S26: Geography and prestige SemAxis by U.S. state. SemAxis projection along two axes, comparing California to Massachusetts universities (left to right), and between the top 20 and geographically-matched bottom 20 universities ranked by the 2018 Times Higher Education ranking of U.S. Universities (bottom to top). Points correspond to universities shown for California (**a**), Arizona (**b**), Washington (**c**), Massachusetts (**d**), Connecticut (**e**), New York (**f**), Texas (**g**), Pennsylvania (**h**), and Florida (**i**). Grey points correspond to all other U.S. universities. Full organization names listed in Table S1.

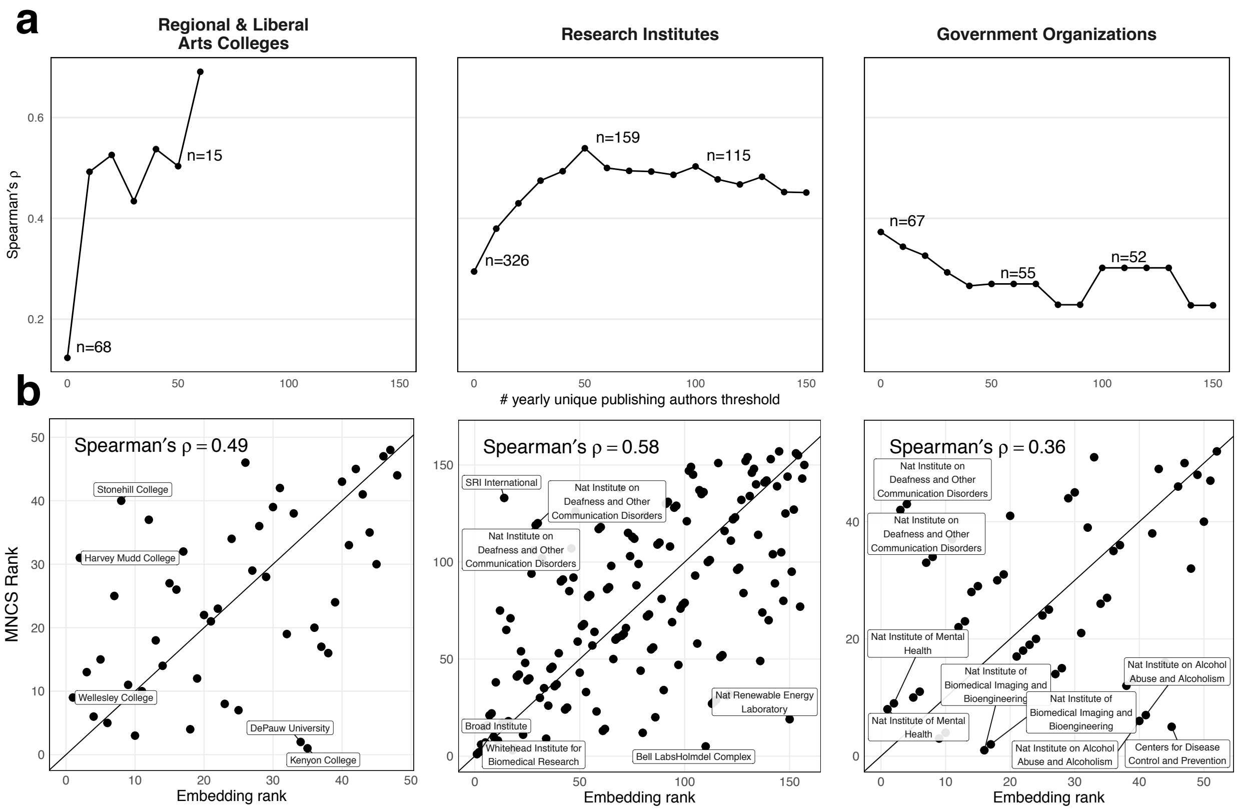

Figure S27: SemAxis reconstructs publication impact in non-university sectors. Comparison between the ranking of organizations in each non-university sector by their citation impact and the embedding rank. Citation impact is calculated as the mean-normalized citation score using papers published in the Web of Science database between 2008 and 2019. The embedding rank is derived by first projecting non-university organizations onto the SemAxis axis formed with poles defined using the top five to geographically-matched bottom five universities ranked by the 2018 Times Higher Education ranking of U.S. Universities. **a** Shows how the correlation between the citation impact and SemAxis rankings differ while varying the size threshold for including an organization. Size is calculated as the mean annualized number of unique authors publishing with that organization. Annotations show the number of organizations remaining at thresholds of 0, 50, and 100. **b**. Comparison of organizations using a size threshold of 10 for regional and liberal arts colleges, and 50 for research institutes and government organizations; these thresholds were chosen as points thresholds of stability in **a**. The impact rank is correlated with the embedding rank for regional and liberal arts colleges with Spearman's $\rho = 0.49$ ($n = 48$), research institutes with Spearman's $\rho = 0.58$ ($n = 159$), and for government organizations with Spearman's $\rho = 0.36$ ($n = 55$). All correlations are significant with $p < 0.001$.

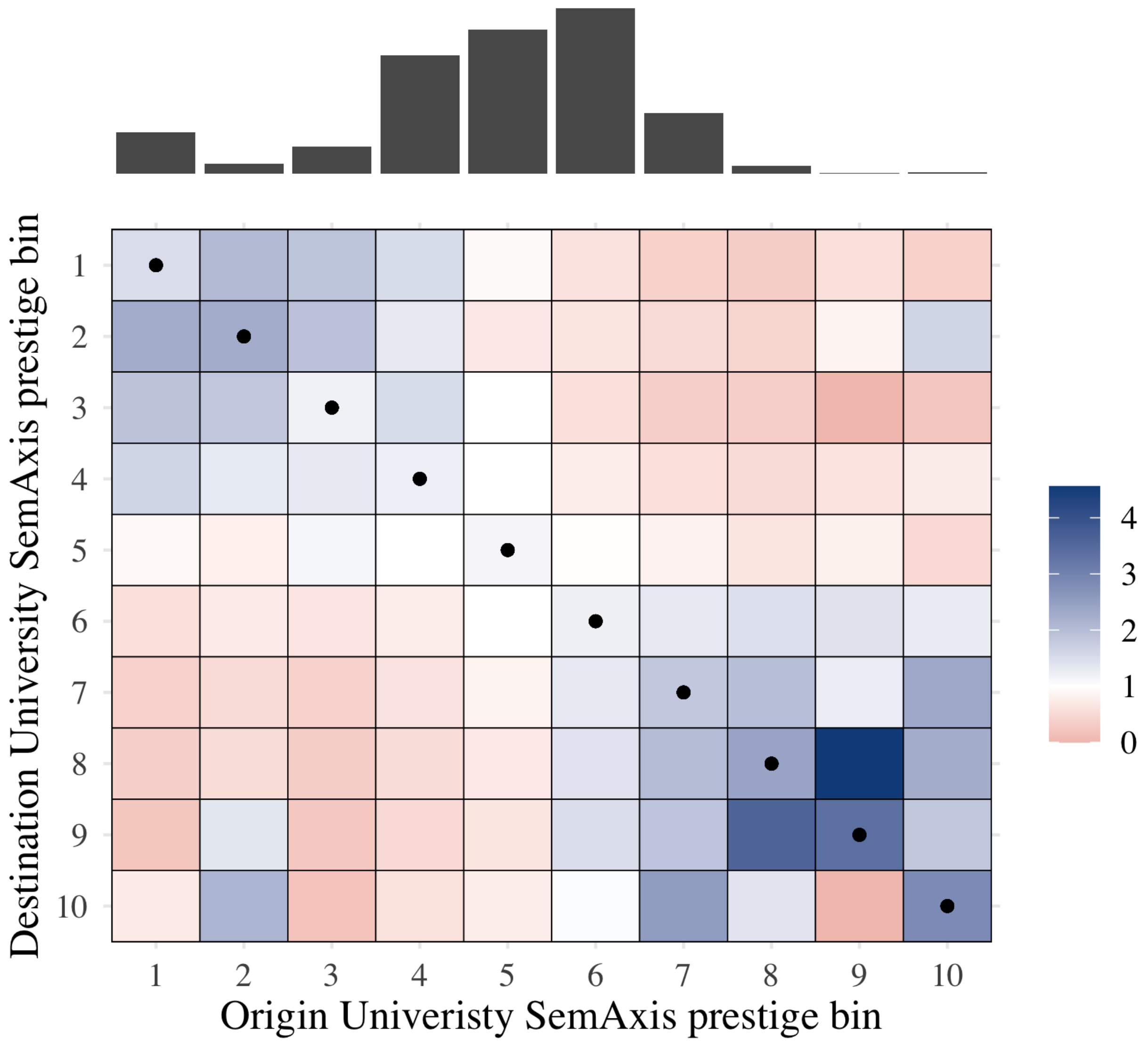

Figure S28: Transitions by SemAxis prestige. Information is sourced for over 100,000 mobile researchers in the United States for whom clear migrations could be identified. SemAxis rank is derived by setting the "elite" pole set according to the top 5 universities by the Times ranking. The continuous range of the SemAxis rank is divided into 10 equally spaced bins. Each cell shows the normalized transitions between the bins of the origin and destination university following the procedure outlined in previous research[49]. Blue indicates that the number of transitions between the bins' is higher than expected when assuming a null model based only on their sizes and total in and out flows, whereas red indicates lower than expected transitions. Dots inside a cell indicate the diagonal. The histogram at the top provides a rough estimation of the distribution of total number of researchers affiliated with universities in each bin. We observe two blocks where internal mobility is over-represented among the most elite, and the least elite universities, whereas middle-ranking universities (accounting for the majority of researchers) show weak affinity in either direction.

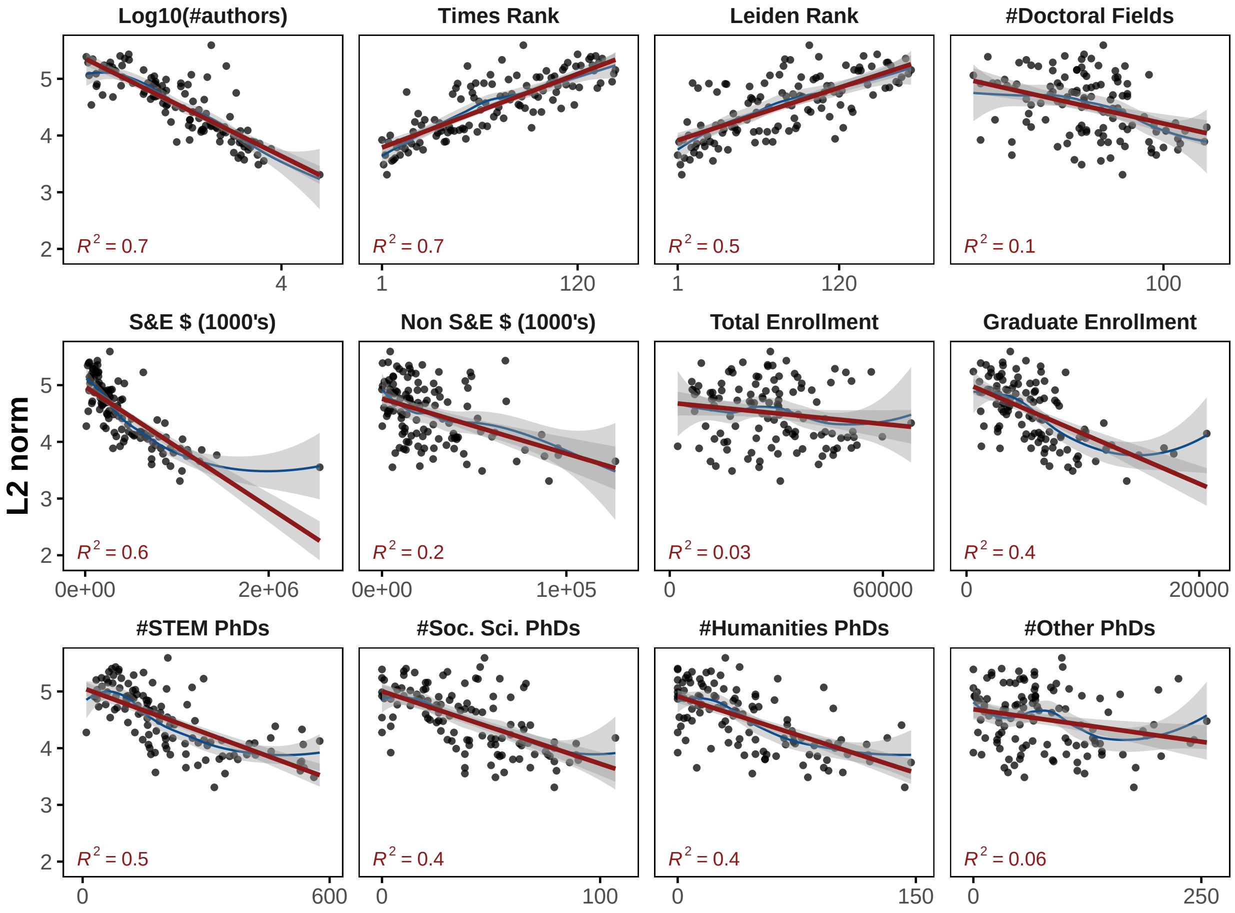

Figure S29: Factors relating to the L2 norm of vectors for U.S. universities Correlation between the L2 norm of organization embedding vectors of U.S. universities and characteristics of U.S. universities. Dots correspond to organizations. The red line is the line of the best fit with corresponding 99% confidence intervals. Red text is the regression estimate. The blue line is the loess regression line with 99% confidence intervals. Number of authors is the average annual count of unique mobile and non-mobile authors. Rankings are derived from the Times Ranking of World Universities, and the Leiden Rankings of Universities. Remaining variables come from the Carnegie Classification of Higher Education Institutions. The factors that best explain s_i are the number of authors, the rank, the amount of Science and Engineering (S&E) funding, and the number of doctorates granted.

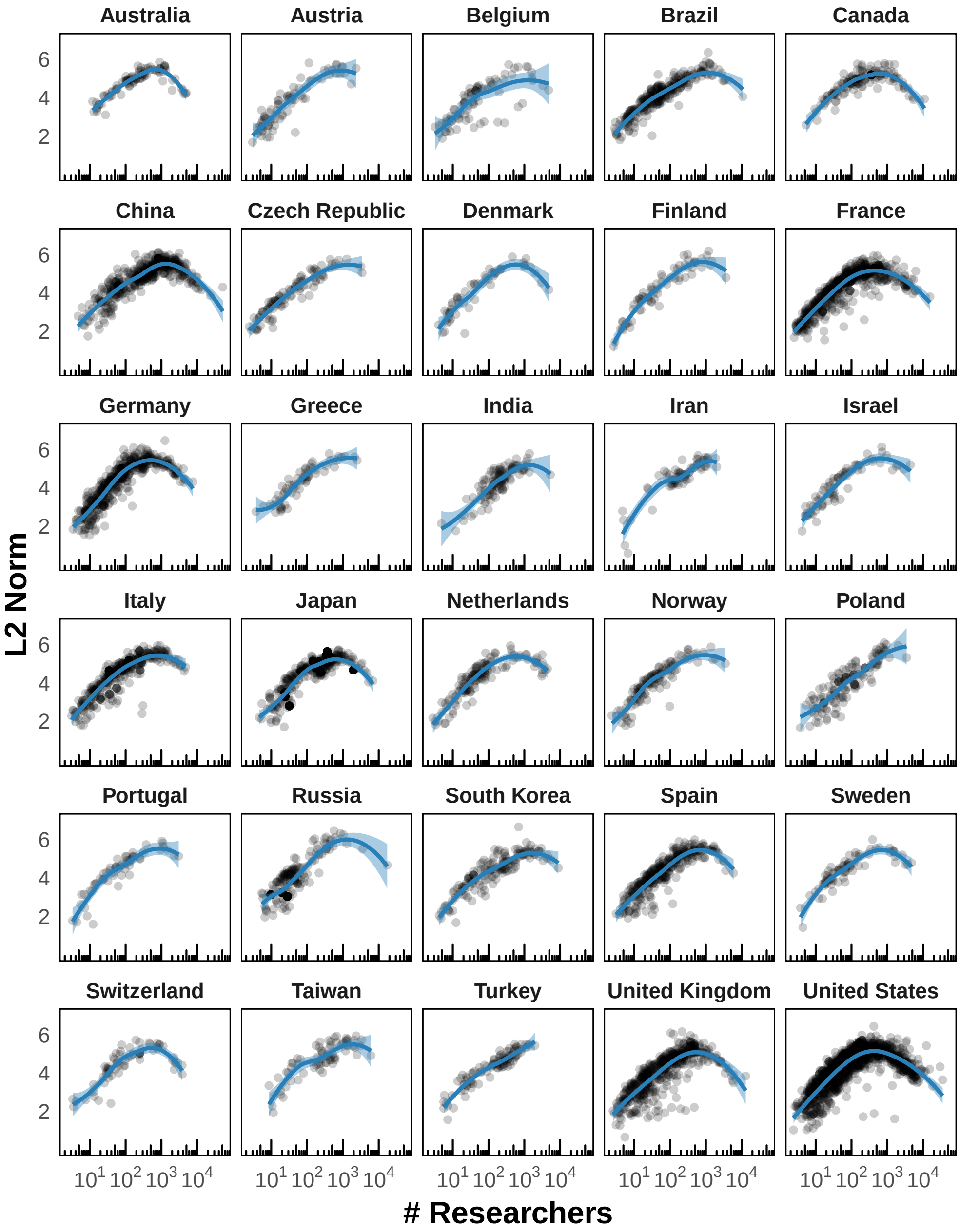

Figure S30: Concave-curve repeats across most of 30 countries with most researchers. Size (L2 norm) of organization embedding vectors compared to their number of researchers for U.S. universities. Loess regression line is shown for each country with 99% confidence intervals. Countries shown are the 30 with the largest number of total unique mobile and non-mobile researchers.

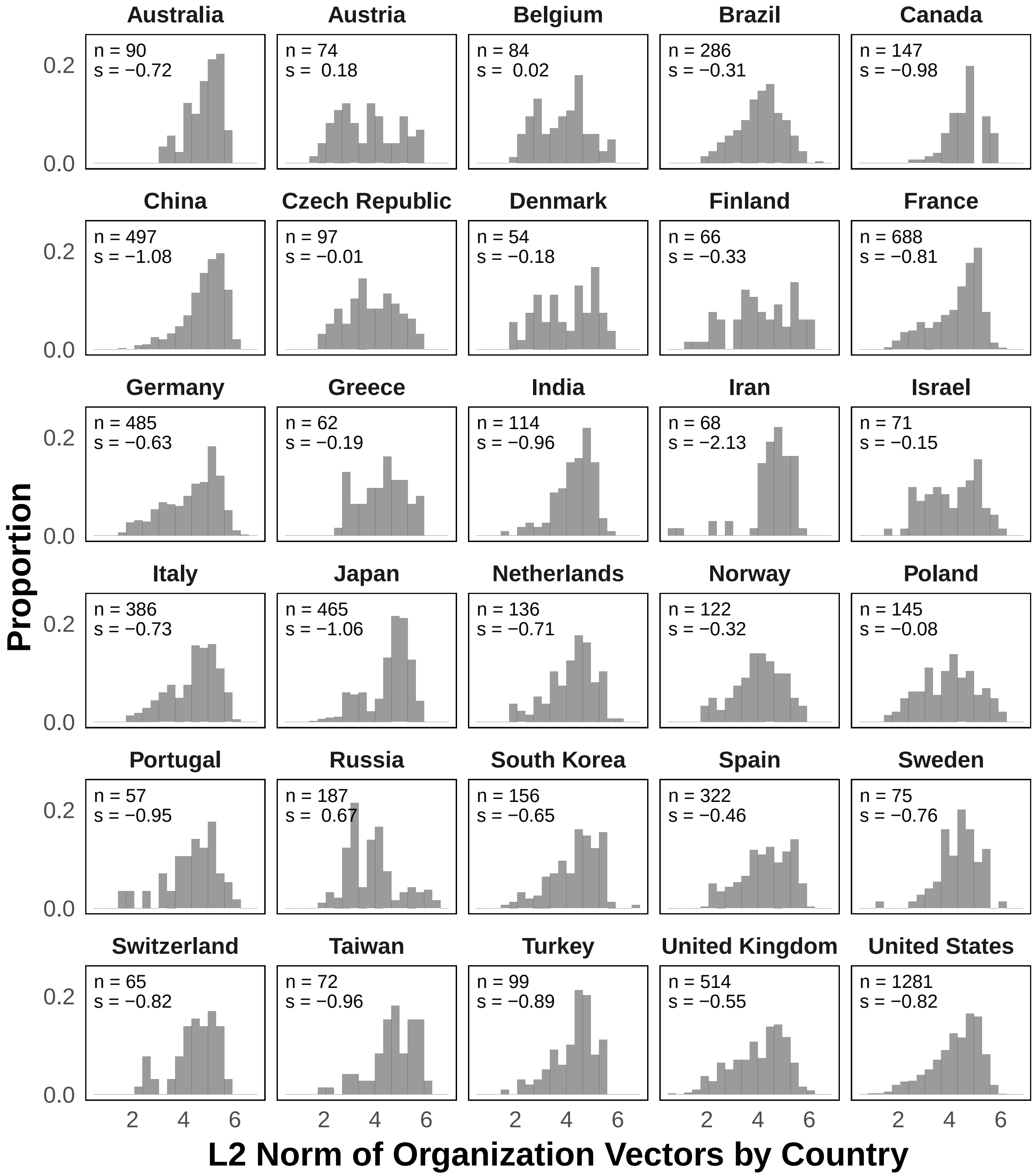

Figure S31: Distribution of organization embedding vector norms by country. Histogram showing the distribution of L2 norm values of organization embedding vectors in each of the 30 countries with the largest number of total unique mobile and non-mobile researchers. Text in each panel shows the number of organizations in the country (n) and the GINI index of inequality of the distribution (g); a small GINI index indicates that the L2 norms of organizations are more balanced, whereas a high GINI value indicates that they are more unequal.

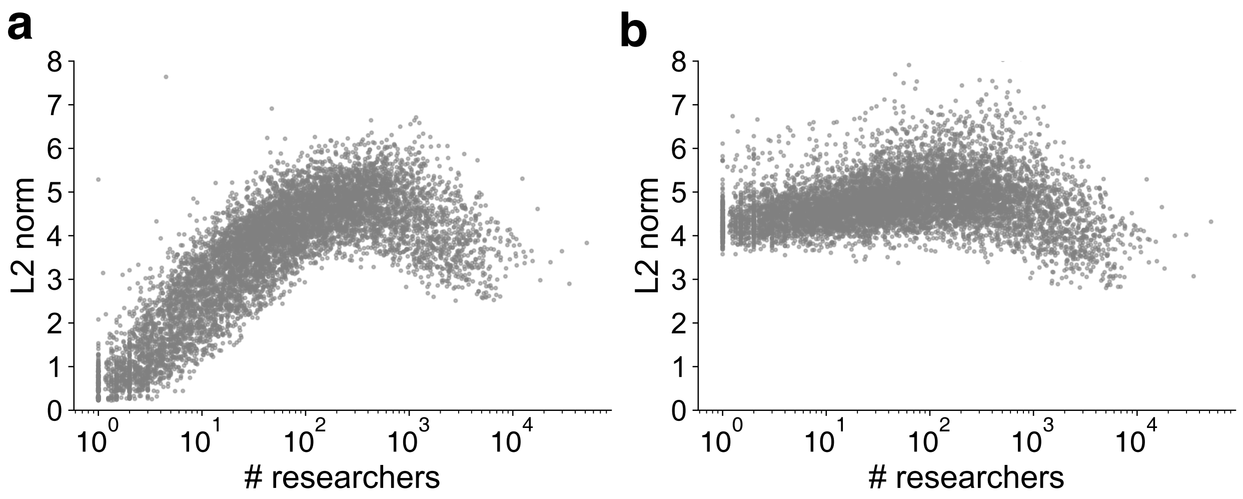

Figure S32: Relationship between organization size and vector L2-norm for synthetic trajectories The x-axis corresponds to the size of an organization, measured as the number of distinct publishing researchers, taken from the real scientific migration data. The y-axis shows the L2-norm calculated for each organization's vector embedded using *word2vec*. Each panel shows vectors generated on synthetic trajectories generated by a process of initializing random walkers (with five steps) on the real weighted organization co-occurence network. (a) random walkers are initialized at each node proportional to the size of the organization. (b) random walkers are initialized with uniform probability across all organizations.

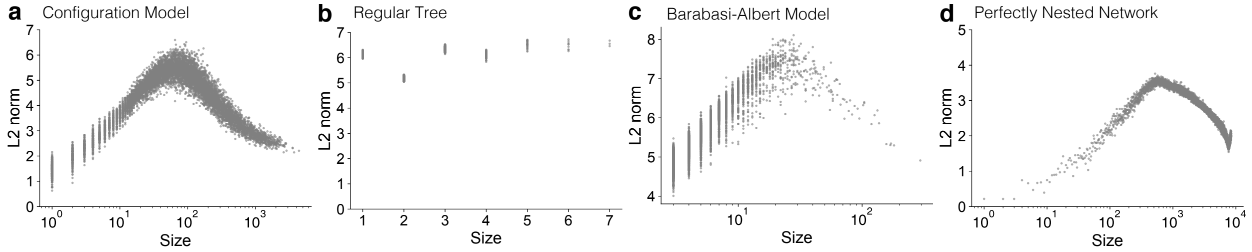

Figure S33: Experiments modelling synthetic network data. The relationship between the size of a node (its degree) and the L2-norm of its embedding vector, generated through the process outlined in the text. Shown for **a.** the configuration model, **b.** regular tree, **c.** Barabási-Albert model, and **d.** a perfectly nested network. The regular tree shows the results split by the depth of each node. The curvilinear relationship emerges in all but the regular tree model, and is particularly prominent in the perfectly nested network.

References

1. Clauset, A., Arbesman, S. & Larremore, D. B. Systematic inequality and hierarchy in faculty hiring networks. en. *Science Advances* **1,** e1400005 (2015).
2. Deville, P. *et al.* Career on the Move: Geography, Stratification and Scientific Impact. *Scientific Reports* **4,** 1–7 (2014).
3. Morgan, A. C., Economou, D. J., Way, S. F. & Clauset, A. Prestige drives epistemic inequality in the diffusion of scientific ideas. *EPJ Data Science* **7,** 40. (2020) (2018).
4. Rodrigues, M. L., Nimrichter, L. & Cordero, R. J. B. The benefits of scientific mobility and international collaboration. *FEMS Microbiology Letters* **363** (2016).
5. Braunerhjelm, P., Ding, D. & Thulin, P. Labour market mobility, knowledge diffusion and innovation. *European Economic Review* **123,** 103386 (2020).
6. Azoulay, P., Zivin, J. S. G. & Sampat, B. N. *The Diffusion of Scientific Knowledge Across Time and Space: Evidence from Professional Transitions for the Superstars of Medicine* Working Paper 16683 (National Bureau of Economic Research, 2011).
7. Kaiser, U., Kongsted, H. C., Laursen, K. & Ejsing, A.-K. Experience matters: The role of academic scientist mobility for industrial innovation. *Strategic Management Journal* **39,** 1935–1958 (2018).
8. Armano, G. & Javarone, M. A. The Beneficial Role of Mobility for the Emergence of Innovation. *Scientific Reports* **7** (2017).
9. Sugimoto, C. R. *et al.* Scientists have most impact when they're free to move. *Nature* **550,** 29–31 (2017).
10. Petersen, A. M. Multiscale impact of researcher mobility. *Journal of The Royal Society Interface* **15,** 20180580 (2018).
11. Jonkers, K. & Cruz-Castro, L. Research upon return: The effect of international mobility on scientific ties, production and impact. *Research Policy* **42,** 1366–1377 (2013).
12. Franzoni, C., Scellato, G. & Stephan, P. The mover's advantage: The superior performance of migrant scientists. *Economics Letters* **122,** 89–93 (2014).
13. Meyer, J.-B. Network Approach versus Brain Drain: Lessons from the Diaspora. *International Migration* **39.**
14. Ioannidis, J. P. A. Global estimates of high-level brain drain and deficit. *FASEB journal: official publication of the Federation of American Societies for Experimental Biology* **18,** 936–939 (2004).
15. Gaillard, A. M. & Gaillard, J. The International Circulation of Scientists and Technologists: A Win-Lose or Win-Win Situation? *Science Communication* **20,** 106–115 (1998).
16. Box, S. & Barsi, E. *The Global Competition for Talent: Mobility of the Highly Skilled* tech. rep. (OECD, 2008).
17. *Measuring Innovation: A New Perspective* tech. rep. (OECD Publishing, 2010).

18. Scellato, G., Franzoni, C. & Stephan, P. Migrant scientists and international networks. *Research Policy* **44** (2015).

19. Robinson-García, N. *et al.* Scientific mobility indicators in practice: International mobility profiles at the country level. *El Profesional de la Informacion* **27,** 511 (2018).

20. Franzoni, C., Scellato, G. & Stephan, P. Foreign-born scientists: mobility patterns for 16 countries. *Nature Biotechnology* **30,** 1250–1253 (2012).

21. Vaccario, G., Verginer, L. & Schweitzer, F. The mobility network of scientists: analyzing temporal correlations in scientific careers. *Applied Network Science* **5** (2020).

22. Albarrán, P., Carrasco, R. & Ruiz-Castillo, J. Geographic mobility and research productivity in a selection of top world economics departments. *Scientometrics* **111,** 241–265 (2017).

23. Markova, Y. V., Shmatko, N. A. & Katchanov, Y. L. Synchronous international scientific mobility in the space of affiliations: evidence from Russia. *SpringerPlus* **5** (2016).

24. Robinson-García, N. *et al.* The many faces of mobility: Using bibliometric data to measure the movement of scientists. *Journal of Informetrics* **13,** 50–63 (2019).

25. Woolley, R. & Turpin, T. CV analysis as a complementary methodological approach : investigating the mobility of Australian scientists. *Research Evaluation* **18,** 143–151 (2009).

26. Sandström, U. Combining curriculum vitae and bibliometric analysis: mobility, gender and research performance. *Research Evaluation* **18,** 135–142 (2009).

27. Cañibano, C., Otamendi, F. J. & Solas, F. International temporary mobility of researchers: a cross-discipline study. *Scientometrics* **89,** 653 (2011).

28. Clauset, A., Shalizi, C. R. & Newman, M. E. Power-law distributions in empirical data. *SIAM review* **51,** 661–703 (2009).

29. Jeh, G. & Widom, J. *Scaling personalized web search* in *Proceedings of the 12th international conference on World Wide Web* (ACM, Budapest, Hungary, 2003), 271–279.

30. Belkin, M. & Niyogi, P. Laplacian eigenmaps for dimensionality reduction and data representation. *Neural computation* **15,** 1373–1396 (2003).

31. Levy, O. & Goldberg, Y. *Neural word embedding as implicit matrix factorization* in *Advances in Neural Information Processing Systems 27* (Curran Associates, Inc., Montreal, Canada, 2014), 2177–2185.

32. Qiu, J. *et al. Network Embedding as Matrix Factorization: Unifying DeepWalk, LINE, PTE, and Node2vec* in *Proceedings of the Eleventh ACM International Conference on Web Search and Data Mining* (Association for Computing Machinery, Marina Del Rey, CA, USA, 2018), 459–467. ISBN: 9781450355810.

33. Xu, H., Caramanis, C. & Sanghavi, S. Robust PCA via outlier pursuit. *IEEE transactions on information theory* **58,** 3047–3064 (2012).

34. Huber, P. J. & Ronchetti, E. M. Robust statistics john wiley & sons. *New York* **1** (1981).

35. Xu, L. & Yuille, A. L. Robust principal component analysis by self-organizing rules based on statistical physics approach. *IEEE Transactions on Neural Networks* **6,** 131–143 (1995).

36. Chandrasekaran, V., Sanghavi, S., Parrilo, P. A. & Willsky, A. S. Rank-sparsity incoherence for matrix decomposition. *SIAM Journal on Optimization* **21,** 572–596 (2011).

37. Candès, E. J., Li, X., Ma, Y. & Wright, J. Robust principal component analysis? *Journal of the ACM (JACM)* **58,** 1–37 (2011).

38. Ma, S., Bassily, R. & Belkin, M. *The power of interpolation: Understanding the effectiveness of SGD in modern over-parametrized learning* in *International Conference on Machine Learning* (2018), 3325–3334.

39. Smith, S., Elsen, E. & De, S. *On the Generalization Benefit of Noise in Stochastic Gradient Descent* in *International Conference on Machine Learning* (2020), 9058–9067.

40. Zhang, G. *et al.* Which algorithmic choices matter at which batch sizes? insights from a noisy quadratic model. *arXiv preprint arXiv:1907.04164* (2019).

41. Waltman, L. *et al.* The Leiden ranking 2011/2012: Data collection, indicators, and interpretation. *Journal of the American Society for Information Science and Technology* **63,** 2419–2432 (2012).

42. D'Angelo, C. A. & van Eck, N. J. Collecting large-scale publication data at the level of individual researchers: a practical proposal for author name disambiguation. *Scientometrics* **123** (2020).

43. Caron, E. & van Eck, N. J. *Large scale author name disambiguation using rule-based scoring and clustering* in *Proceedings of the 14th Science and Technology Indicators Conference* (Leiden University, Leiden, Netherlands, 2014), 79–86.

44. Chinchilla-Rodríguez, Z. *et al.* A Global Comparison of Scientific Mobility and Collaboration According to National Scientific Capacities. *Frontiers in Research Metrics and Analytics* **3** (2018).

45. Chinchilla-Rodríguez, Z., Bu, Y., Robinson-García, N., Costas, R. & Sugimoto, C. R. Travel bans and scientific mobility: utility of asymmetry and affinity indexes to inform science policy. *Scientometrics* **116,** 569–590 (2018).

46. Gutmann, M. & Hyvärinen, A. *Noise-contrastive estimation: A new estimation principle for unnormalized statistical models* in *Proceedings of the Thirteenth International Conference on Artificial Intelligence and Statistics* **9** (PMLR, Chia Laguna Resort, Sardinia, Italy, 2010), 297–304.

47. Dyer, C. *Notes on Noise Contrastive Estimation and Negative Sampling* 2014. arXiv: `1410.8251 [cs.LG]`.

48. Schakel, A. M. J. & Wilson, B. J. Measuring Word Significance using Distributed Representations of Words. *CoRR* **abs/1508.02297** (2015).

49. Park, J. *et al.* Global labor flow network reveals the hierarchical organization and dynamics of geo-industrial clusters. *Nature Communications* **10** (2019).
